# Exploring the effect of case management in homelessness per components: A systematic review of effectiveness and implementation, with meta‐analysis and thematic synthesis

**DOI:** 10.1002/cl2.1329

**Published:** 2023-05-17

**Authors:** Alison L. Weightman, Mark J. Kelson, Ian Thomas, Mala K. Mann, Lydia Searchfield, Simone Willis, Ben Hannigan, Robin J. Smith, Rhiannon Cordiner

**Affiliations:** ^1^ Specialist Unit for Review Evidence (SURE) Cardiff University Cardiff UK; ^2^ Department of Mathematics and Statistics, Faculty of Environment, Science and Economy University of Exeter Exeter UK; ^3^ Wales Institute of Social and Economic Research and Data (WISERD) Cardiff University Cardiff UK; ^4^ Mental Health Nursing, School of Healthcare Sciences Cardiff University Cardiff UK; ^5^ School of Social Sciences Cardiff University Cardiff UK

## Abstract

**Background:**

Adequate housing is a basic human right. The many millions of people experiencing homelessness (PEH) have a lower life expectancy and more physical and mental health problems. Practical and effective interventions to provide appropriate housing are a public health priority.

**Objectives:**

To summarise the best available evidence relating to the components of case‐management interventions for PEH via a mixed methods review that explored both the effectiveness of interventions and factors that may influence its impact.

**Search Methods:**

We searched 10 bibliographic databases from 1990 to March 2021. We also included studies from Campbell Collaboration Evidence and Gap Maps and searched 28 web sites. Reference lists of included papers and systematic reviews were examined and experts contacted for additional studies.

**Selection Criteria:**

We included all randomised and non‐randomised study designs exploring case management interventions where a comparison group was used. The primary outcome of interest was homelessness. Secondary outcomes included health, wellbeing, employment and costs. We also included all studies where data were collected on views and experiences that may impact on implementation.

**Data Collection and Analysis:**

We assessed risk of bias using tools developed by the Campbell Collaboration. We conducted meta‐analyses of the intervention studies where possible and carried out a framework synthesis of a set of implementation studies identified by purposive sampling to represent the most ‘rich’ and ‘thick’ data.

**Main Results:**

We included 64 intervention studies and 41 implementation studies. The evidence base was dominated by studies from the USA and Canada. Participants were largely (though not exclusively) people who were literally homeless, that is, living on the streets or in shelters, and who had additional support needs. Many studies were assessed as having a medium or high risk of bias. However, there was some consistency in outcomes across studies that improved confidence in the main findings.

**Case Management and Housing Outcomes:**

Case management of any description was superior to usual care for homelessness outcomes (standardised mean difference [SMD] = −0.51 [95% confidence interval [CI]: −0.71, −0.30]; *p* < 0.01). For studies included in the meta‐analyses, Housing First had the largest observed impact, followed by Assertive Community Treatment, Critical Time Intervention and Intensive Case Management. The only statistically significant difference was between Housing First and Intensive Case Management (SMD = −0.6 [–1.1, −0.1]; *p* = 0.03) at ≥12 months. There was not enough evidence to compare the above approaches with standard case management within the meta‐analyses. A narrative comparison across all studies was inconclusive, though suggestive of a trend in favour of more intensive approaches.

**Case Management and Mental Health Outcomes:**

The overall evidence suggested that case management of any description was not more or less effective compared to usual care for an individual's mental health (SMD = 0.02 [−0.15, 0.18]; *p* = 0.817).

**Case Management and Other Outcomes:**

Based on meta‐analyses, case management was superior to usual care for capability and wellbeing outcomes up to 1 year (an improvement of around one‐third of an SMD; *p* < 0.01) but was not statistically significantly different for substance use outcomes, physical health, and employment.

**Case Management Components:**

For homelessness outcomes, there was a non‐significant trend for benefits to be greater in the medium term (≤3 years) compared to long term (>3 years) (SMD = −0.64 [−1.04, −0.24] vs. −0.27 [−0.53, 0]; *p* = 0.16) and for in‐person meetings in comparison to mixed (in‐person and remote) approaches (SMD = −0.73 [−1.25,−0.21]) versus −0.26 [−0.5,−0.02]; *p* = 0.13). There was no evidence from meta‐analyses to suggest that an individual case manager led to better outcomes then a team, and interventions with no dedicated case manager may have better outcomes than those with a named case manager (SMD = −0.36 [−0.55, −0.18] vs. −1.00 [−2.00, 0.00]; *p* = 0.02). There was not enough evidence from meta‐analysis to assess whether the case manager should have a professional qualification, or if frequency of contact, case manager availability or conditionality (barriers due to conditions attached to service provision) influenced outcomes. However, the main theme from implementation studies concerned barriers where conditions were attached to services.

**Characteristics of Persons Experiencing Homelessness:**

No conclusions could be drawn from meta‐analysis other than a trend for greater reductions in homelessness for persons with high complexity of need (two or more support needs in addition to homelessness) as compared to those with medium complexity of need (one additional support need); effect sizes were SMD = −0.61 [−0.91, −0.31] versus −0.36 [−0.68, −0.05]; *p* = 0.3.

**The Broader Context of Delivery of Case Management Programmes:**

Other major themes from the implementation studies included the importance of interagency partnership; provision for non‐housing support and training needs of PEH (such as independent living skills), intensive community support following the move to new housing; emotional support and training needs of case managers; and an emphasis on housing safety, security and choice.

**Cost Effectiveness:**

The 12 studies with cost data provided contrasting results and no clear conclusions. Some case management costs may be largely off‐set by reductions in the use of other services. Cost estimates from three North American studies were $45–52 for each additional day housed.

**Authors' Conclusions:**

Case management interventions improve housing outcomes for PEH with one or more additional support needs, with more intense interventions leading to greater benefits. Those with greater support needs may gain greater benefit. There is also evidence for improvements to capabilities and wellbeing. Current approaches do not appear to lead to mental health benefits. In terms of case management components, there is evidence in support of a team approach and in‐person meetings and, from the implementation evidence, that conditions associated with service provision should be minimised. The approach within Housing First could explain the finding that overall benefits may be greater than for other types of case management. Four of its principles were identified as key themes within the implementation studies: No conditionality, offer choice, provide an individualised approach and support community building. Recommendations for further research include an expansion of the research base outside North America and further exploration of case management components and intervention cost‐effectiveness.

## 1 PLAIN LANGUAGE SUMMARY

### 1.1 Case management interventions improve housing stability for people experiencing homelessness and the effects may be increased with intensive support

Homelessness is an important problem and case management support may provide part of the solution. This review includes some guidance for current practice and policy and recommendations for future research, including an expansion of the research base outside North America.

### 1.2 What is this review about?

Many millions of people experience homelessness, potentially leading to poorer health and wellbeing outcomes, and a lower life expectancy. We present evidence on a wide range of interventions that include a case manager to help the individual client to find stable housing.

**What is the aim of this review?**

This Campbell systematic review examines studies of case management programmes for people experiencing homelessness to help identify the components most likely to increase the chances of improvements in housing, health and wider outcomes.

### 1.3 What studies are included?

We looked, specifically, for any findings to help identify the individual components of case management such as the period over which support is provided, the number of clients per case manager, and whether there are conditions attached to this support from the client's point of view.

We explored the effects of these interventions on homelessness and other outcomes such as mental health, substance use, physical heath, wellbeing and employment.

### 1.4 What are the main findings of this review?

#### 1.4.1 Case management effectiveness overall

Any type of case management clearly improves homelessness outcomes for people with additional support needs, and this may be more effective for people who also have greater levels of additional need for support. Case management also increases wellbeing for the population in the included studies, at least in the short term.

Across the full body of evidence, it does not appear that the included interventions improve mental health, and there is no evidence of improvement in employment, physical health or substance use.

Time spent in stable housing may be increased when case management is more intensive. The multi‐component Housing First approach may be more effective than other types of intensive case management.

#### 1.4.2 Case management components

In terms of housing outcomes, support for up to three years leads to improvements in stable housing. These benefits may be reduced over the longer term but still persist, suggesting that very long‐term support should be provided.

In‐person meetings with the case manager appear to be beneficial when compared with remote or mixed (remote/in‐person) meetings but many studies did not describe meeting location(s).

Although there was not enough evidence from the intervention studies, there is consistent evidence from the implementation studies that any barriers attached to case management support (i.e. conditions that must be met to receive that support) should be minimised.

#### 1.4.3 Supporting case managers

A number of themes arise from the implementation studies that are relevant to the components of a case management programme. These include the importance of a close working relationship across agencies; provision for the non‐housing support and training needs of clients experiencing homelessness; community support and development for the newly‐housed; providing for the emotional support and training needs of case managers; and giving clients choice in relation to the type of housing provided.

#### 1.4.4 Cost effectiveness

The available studies vary in their findings. It is likely that case management is more costly than usual care but may be cost‐effective if society is ‘willing to pay’ a certain amount to support people experiencing homelessness into stable housing.

### 1.5 What do the findings of this review mean?

Case management helps people experiencing homelessness who have additional support needs to obtain stable accommodation, and is even more helpful for those with higher levels of support needs.

High intensity multicomponent approaches such as Housing First may lead to greater benefits. There is also some evidence for improvements to capabilities and wellbeing but, notably, they do not appear to impact mental health outcomes any more than usual care.

There is some evidence that case management support should be long term, that meetings in person with clients are beneficial, and any conditions associated with provision of the service should be minimised.

### 1.6 How up to date is this review?

The review authors searched for studies up to March 2021.

## 2 BACKGROUND

### 2.1 The problem, condition or issue

Homelessness, broadly defined as the lack of minimally adequate housing (Busch‐Geertsema, 2016), is a human rights issue of global concern. Experienced by an estimated 150 million people worldwide, homelessness can lead to a range of social and health inequalities, including increased mortality. There is an extensive evidence base on what works in preventing and alleviating homelessness. Through meta‐analyses of these disparate studies, researchers can develop a more robust estimate of the overall effects of interventions. Case management, as a form of care coordination (Hannigan, 2018; Lukersmith, 2016), has shown itself to be one form of intervention with some promise in leading to more stable housing after experiencing homelessness, and in reducing its negative impacts (Munthe‐Kaas, 2018; Ponka, 2020). However, there is a lack of clarity around which form and components of case management make this intervention effective—this is the problem the current review sought to address. By deconstructing case management, this review aims to improve the design of services to allow them to focus on elements that maximise impact, whilst working within increasingly stretched housing‐related support services budgets—something of particular importance in the current climate of government austerity and global economic stagnation worsened by the 2022 Russian invasion of Ukraine.

### 2.2 Description of the condition

Individuals or households who are currently experiencing, or are at risk of experiencing homelessness.

### 2.3 Description of the intervention

A case manager or team of people assess, plan, and facilitate access to a range of services for a participant (Ponka, 2020). The broad principles of case management are that it is participant driven, pragmatic, flexible, anticipatory, culturally sensitive and offers a single point of contact (Vanderplasschen, 2004). Case management often includes practical support, help with the development of independent living skills, acute support during crises, and support for healthcare and contacts in social and professional support systems (de Vet, 2013).

To a certain extent, all homelessness services adopt some form of case management as they assess, plan, and coordinate help (Homeless Link, 2019). There are however formalised models of case management structured to fit specific care contexts and the issues faced by people.

Case‐management models and usual care vary across countries but, from the literature on case management for people experiencing homelessness (PEH) (de Vet, 2013; Homeless Link, 2019; Munthe‐Kaas, 2018; Ponka, 2020), there are five main models:

- Broker Case Management (BCM)—Case managers assess people and their needs and purchase or coordinate appropriate services. Being mainly used with people facing less complex issues, such as those with mainly housing related issues, there is very little service provision by the case worker, who may have a large case load.
- Standard Case Management (SCM)—Similar to the brokerage model in terms of the low intensity of work and the target group, the SCM model is less aligned to the purchase of services for the participant. There is also some level of relationship between case manager and participant, unlike the broker model where this relationship is not important.
- Intensive Case Management (ICM)—The case manager provides a high level of support to the participant to access other services and/or resolve issues of relevance. As ICM involves ongoing comprehensive support, caseloads are kept intentionally small.
- Assertive Community Treatment (ACT)—Rather than a single case manager, ACT draws on a multidisciplinary team or network to support participants within a service.
- Critical Time Intervention (CTI)—Offers time‐limited and structured support during periods of transition, for example moving into permanent accommodation. The aim of CTI is to provide continuity of care during periods of change.

Each of the case management models identified above is structured in distinct ways. Munthe‐Kaas 2018 describe the different case management models in terms of eight characteristics: (1) focus of services, (2) duration of services, (3) average caseload, (4) whether the service involves outreach, (5) whether the service involves coordination or service provision, (6) who is responsible for the participant's care, (7) the importance of the participant‐case manager relationship, and (8) intensity of service. de Vet et al. (de Vet, 2013) and Ponka et al. (Ponka, 2020) also include the target population when describing the use of case management for PEH.

The specific model of Housing First should be mentioned in this context given its increasing use within the sector (Woodhall‐Melnik, 2015). Although the model includes case management (either ACT or ICM) it is a multicomponent intervention that offers more than case management alone. ACT itself is a multicomponent model based on fidelity to a range of provisions often measured via the Dartmouth Assertive Community Treatment Scale based on 28 fidelity criteria (Teague, 1998).^1^ The fidelity criteria for ICM are much less clear and Dieterich et al. (Dieterich, 2017) describe ICM as evolving from ACT and case management, where ICM emphasises the importance of a small case load and high‐intensity input. Criteria for selection of PEH for either ICM or ACT within Housing First does not seem to be clear other than for the At Home/Chez Soi study where these are described as high and moderate needs (Goering, 2014).

**Figure 1:**
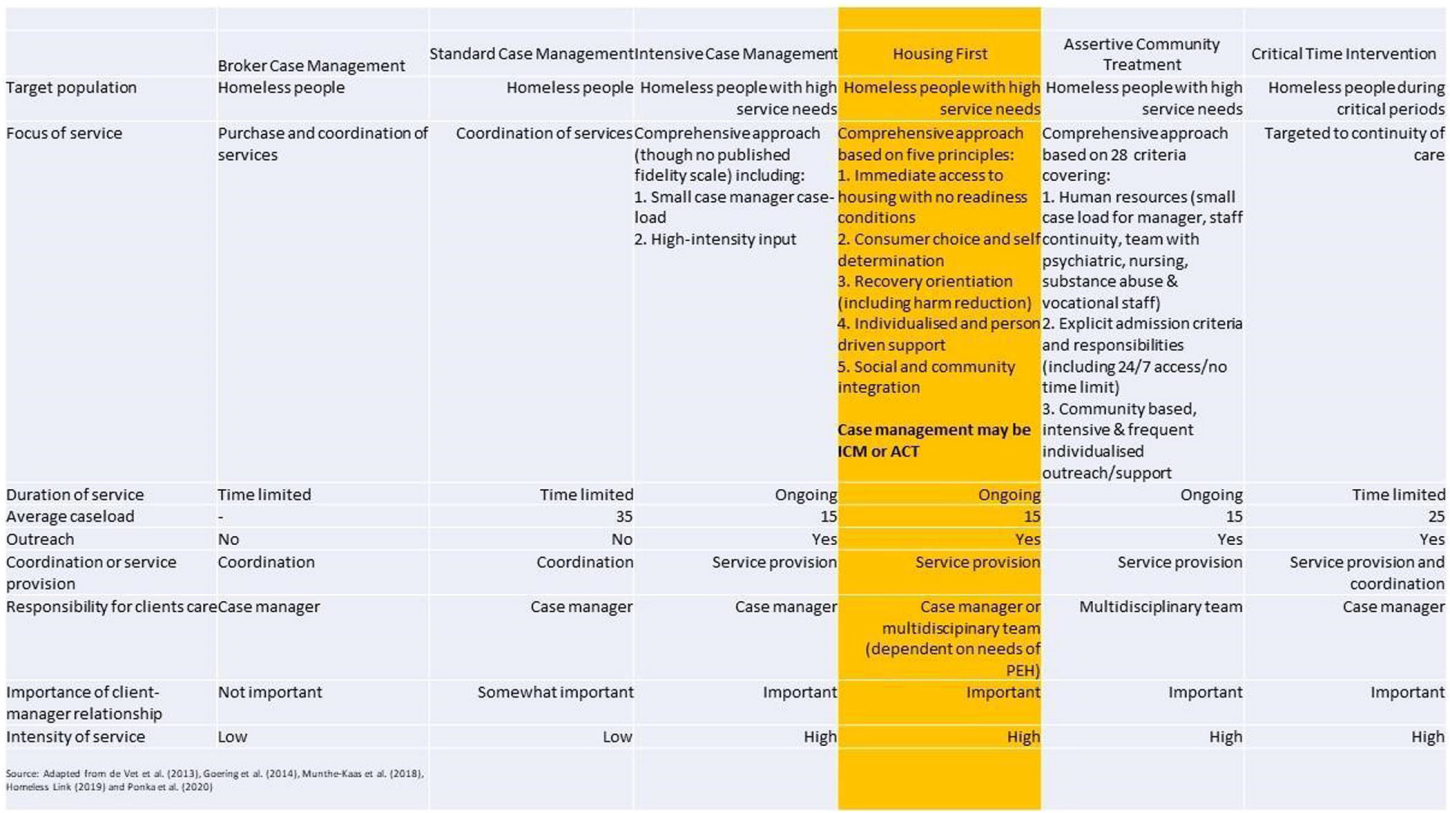
Characteristics of case management models.

Brief descriptions of each of the case management models are provided in Figure 1 with Housing First highlighted in orange, as a multi‐component intervention that includes either ICM or ACT.

### 2.4 How the intervention might work

PEH can experience barriers to accessing services. Having a case manager act in a position of authority when interacting with services could potentially overcome or lessen these barriers. However, this view assumes that PEH lack self‐determination (Thomas, 2012). Case management can therefore be structured to empower PEH to set and realise their own goals (a strengths‐based approach).

Where PEH experience multiple forms of exclusion and have multiple support issues, this may require them to engage with multiple services, for example housing and mental health services. However, a lack of co‐ordination amongst services may prevent PEH from receiving the holistic assistance they need in a timely manner—particularly when combined with barriers in accessing services on their own. As a form of service co‐ordination, case management offers a centralised point of contact in referring and brokering access or acting on behalf of the person, and in some models by providing ongoing support with services.

### 2.5 Why it is important to do this review

Systematic reviews and meta‐analyses of case management with PEH have found that this intervention can lead to improvement in people's outcomes (Coldwell, 2007; de Vet, 2013; Munthe‐Kaas, 2018). The most recent published review by Ponka (Ponka, 2020) found that standard case management had both limited and short‐term effects on substance use and housing outcomes and showed potential to increase hostility and depression. Intensive case management substantially reduced the number of days spent homeless (standardised mean difference [SMD] = −0.22; 95% confidence interval [CI]: −0.40 to −0.03), as well as substance and alcohol use. Critical time interventions and assertive community treatment were found to have a protective effect in terms of re‐hospitalisations and a promising effect on housing stability. Assertive community treatment was found to be cost‐effective compared to standard case management.

However, there is only limited evidence of the relative roles of the different types and components of case management in influencing outcomes amongst PEH. Furthermore, PEH are largely treated as a homogenous group in previous reviews, when homelessness can cover a range of different experiences (Amore, 2011; Edgar, 2009) and have different causal factors. There are therefore important differences in people's experience of homelessness, for example along the lines of gender (Bretherton, 2017), that may impact which components of case management are more appropriate and effective with different groups of PEH.

This review adds value to the reviews described above by taking a mixed methods approach, including interventional and observational research. The review team has attempted to disentangle the components of the case management models explored in the research literature, using statistical analysis where feasible. The findings from narrative and any meta‐analytical syntheses have been supported by an analysis of the themes identified from implementation/qualitative research with respect to possible factors that may impact on implementation success.

## 3 OBJECTIVES

To carry out a mixed methods review to summarise current evidence relating to the components of case‐management interventions for PEH.

To summarise what is known about:

1. Component effectiveness/cost‐effectiveness.
2. Case management effectiveness, and its components, in relation to the characteristics of the recipients of this intervention.
3. Implementation and process factors that may impact on intervention delivery in terms of case management approach, intervention components and recipient characteristics.

See Data Extraction and Management for details.

## 4 METHODS

### 4.1 Criteria for considering studies for this review

#### 4.1.1 Types of studies

This is a mixed methods review including both quantitative (effectiveness) and qualitative (implementation) studies. Effectiveness studies were synthesised with a meta‐analysis where feasible; while a framework synthesis was used to synthesise the factors that may impact on implementation.

Methods followed those in the published protocol (Weightman, 2022) and findings are reported in line with the Campbell MECCIR reporting standards.^2^

*Quantitative studies*: We included all quantitative study designs where a comparison group was used. This includes randomised controlled trials, quasi‐experimental designs, matched comparisons and other study designs that attempt to isolate the impact of the intervention on homelessness using appropriate statistical modelling techniques. These designs were chosen as the use of a control group helps ensure that changes observed in treatment group participants are due to effects of the intervention, and not attributable to other factors.

As randomised controlled trials are accepted as more equipped to infer causality than non‐randomised studies, the potential impact of non‐randomised study designs on effect sizes was explored as part of the analysis of heterogeneity. Where feasible, for the primary outcomes, sensitivity analyses were carried out on the basis of study design and risk of bias assessment.

Studies had to include an alternative case‐management approach or an inactive comparison condition that could include:

- No treatment.
- Usual care.
- Waiting list where service providers or service users are randomly assigned to receive the intervention at a later date. Details of what happens to waitlisted participants will be extracted.
- Attention control, where participants receive some contact from researchers but both participants and researchers are aware that this is not an active intervention.
- Placebo where participants perceive that they are receiving an active intervention but the researchers regard the treatment as inactive.

Studies with no control or comparison group (e.g., pretest/posttest), unmatched controls or national comparisons with no attempt to control for relevant covariates were not included. Case studies, opinion pieces or editorials were not included.

*Implementation studies*: We included all research designs where data were collected on the views and experiences of service users or providers that have some bearing on factors that may impact on the effectiveness of the case management approach. In addition to specific qualitative study designs (such as focus groups and interviews), mixed methods studies, process evaluations, surveys, observational studies (e.g., ethnographic) and secondary data analyses were included.

We searched for data that enabled a deeper understanding of why an intervention does (or does not) work as intended, for whom and under what circumstances.

As per protocol (Weightman, 2022) it was agreed that, if a very large number of studies were identified, the review team would use a ‘Best Fit Approach’ based on a sample of studies using formal qualitative methods, and which are deemed most relevant (see Assessment of Findings).

#### 4.1.2 Types of participants

This review used a broad definition of PEH to systematically search for studies, as: (1) people without accommodation, such as those living on the streets, (2) people accessing housing that is either temporary or tied to institutional care, such as hostels, shelters, and other temporary accommodation, or people about to be released from prison without accommodation to return to, (3) people in severely inadequate and/or insecure housing, such as people in overcrowded conditions and ‘sofa surfers’ (Busch‐Geertsema, 2016).

Studies of case management interventions that included the above groups of PEH were retained irrespective of age, gender, or household type. Studies included populations from the Global North, given that the social and economic contexts of homelessness are likely to be vastly different to those faced in the Global South (Busch‐Geertsema, 2016).

However, the evidence base accumulated related mainly, though not exclusively, to groups 1 and 2, that is, people who were living on the streets, in hostels, or who had left prison/the armed forces. Furthermore, as indicated in the overview of studies, participants almost exclusively had medium to high levels of additional support needs—being defined as one or more issues in addition to their homelessness. Many of the studies relate to Housing First interventions combining forms of case management with other housing interventions, and which are routinely targeted at people with high support needs and/or entrenched experiences of homelessness (O'Sullivan, 2020). As such, it should be recognised that the participants in this study reflect a sub‐group of the population of PEH.

#### 4.1.3 Types of interventions

Interventions included within this systematic review were those with an explicit description of a case‐management approach whereby a designated case manager supports the person experiencing homelessness by facilitating integrated access to health and social services and accommodation support.

There are five established case‐management approaches as well as the multi‐component Housing First approach (Woodhall‐Melnik, 2015) (see Description of the Intervention; Figure 1).

All interventions were included that claimed to adopt a case‐management approach.

Comparison conditions included usual care or an alternative service/intervention. Usual care (sometimes called treatment as usual or usual services) is not often clearly defined (Ponka, 2020). As in de Vet (2013) the components of usual care were summarised where specified. The assumption made was that usual care comprised the services that clients had been receiving before study enrolment, including access to housing and support services offered within the community (Munthe‐Kaas, 2018) while noting that these vary hugely across countries, locations and interventions and are diverse (de Vet, 2013).

#### 4.1.4 Types of outcome measures

The review explored a range of housing, health and wellbeing outcomes within the effectiveness studies and these are summarised below.

Regarding implementation studies, outcomes related to views and experiences of service users and providers of case management interventions. Such studies were summarised via a Framework synthesis (see Treatment of Implementation Studies).

##### 4.1.4.1 Primary outcomes

**Figure 2:**
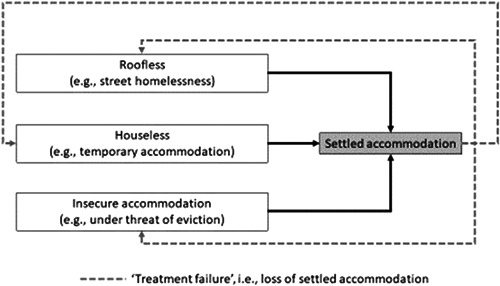
Primary outcome—Settled accommodation.

In keeping with Keenan (Keenan, 2020) this review primarily addressed how interventions can reduce homelessness and/or increase housing stability. Where case management interventions lead to settled accommodation, for households that lose that settled accommodation and return to *any* state of homelessness, this was considered ‘treatment failure’ (Figure 2). Measures in the reviewed literature included outcomes such as days housed, proportion of time on the streets, stability of housing.

Where feasible, the *primary outcomes* were explored in relation to the characteristics of case management and the individuals receiving it (objectives 1 and 2).

##### 4.1.4.2 Secondary outcomes

If studies reported any of the outcomes listed below in addition to the primary outcome, we extracted data on those outcomes. These secondary outcomes were not part of the inclusion criteria.

- Access to health and social care services
- Physical health
- Mental health
- Substance use
- Crime/criminalisation
- Employment and income
- Capabilities and wellbeing
- Cost/cost effectiveness of intervention

Mental health outcomes included psychiatric inpatient and outpatient treatment, mental health scores (e.g., Brief Psychiatric Rating Scale, Psychiatric Index Score) and mental illness diagnoses. We also paid attention to implementation and acceptability of interventions and included information on attrition rates or ‘dropout’ from interventions.

Physical health outcomes included physical mobility, the physical component score of the SF‐36, SF‐8 and SF‐12, the physical health symptoms checklist, a medical index score, and a physical health symptoms checklist.

Substance use outcomes included number of days when alcohol was consumed (over times frames of 30 days, 6 months and 18 months), days of substance use in the past 90 days, percentage days of substance use in the past 90 days, average number of drinks consumed daily over the past year.

Capabilities and wellbeing outcomes included quality of life (QOLI‐20), Lehman's Brief Quality of Life Interview, SF‐36, Schizophrenia quality of life (S‐QoL‐18), and the self‐efficacy scale (SES), community function (the Multnomah Community Ability Scale), and a community integration scale.

Employment and income outcomes included days employed in the past 30 days, and monthly income.

There was not enough data for analysis for access to health and social care services or crime/criminalisation outcomes.

Where possible, results were synthesised within meta‐analyses. Where there was little information on an outcome, or study designs and outcomes varied hugely (e.g., for the cost outcomes), the findings are presented as a narrative only.

Inclusion/exclusion criteria for the review are summarised in Supporting Information: Appendix 1 and are in keeping with the PICO (population, intervention, control, outcomes) criteria outlined above.

### 4.2 Search methods for identification of studies

This systematic review was based on evidence identified from a specific search for all types of research study published since 1990 to March 2021 exploring case management in homelessness.

This topic‐specific search supplemented the large set of studies already identified from existing evidence and gap maps (EGMs) relating to homelessness (White, 2018a; White, 2018b; White, 2020) and a recent systematic review looking at case‐management in homelessness (Ponka, 2020). At the time of the searching for this review, the most recent search for intervention studies for the EGM was completed in March 2020 and the most recent search for qualitative and other studies for the implementation EGM was completed in January 2021. The earliest study identified from these searches was published in 1992.

#### 4.2.1 Electronic searches

Electronic searches were carried out in 10 databases and completed on 1 March 2021 (Supporting Information: Appendix 2).

#### 4.2.2 Searching other resources

The web sites explored by White (2018b), White (2020) for the EGM in March 2020 were browsed to 29 March 2021 for any publications in 2020 and 2021 (see Supporting Information: Appendix 2) and relevant studies were sought from the experts supporting the protocol development. Reference lists of any systematic reviews identified and all included studies were scanned for other relevant studies. There were no language restrictions.

### 4.3 Data collection and analysis

#### 4.3.1 Description of methods used in primary research

For evidence on effectiveness, interventions including RCTs and other designs with a comparison group measuring the effectiveness of the case management approach where the comparison group may be usual practice or an alternative intervention.

For evidence on implementation, qualitative and other research gathering views, opinions and experiences of relevance to factors that may impact on the effectiveness of the case management intervention.

#### 4.3.2 Criteria for determination of independent findings

It is important to ensure that the effects of an individual intervention are only counted once and the following conventions therefore applied.

Where the same outcome construct was measured but across multiple time domains, such as through the collection of both post‐test and further follow‐up data, the analysis was conducted and reported separately for different time points. We split outcome timings into categories of ‘less than a year post‐intervention’ and ‘a year or longer post‐intervention’.

Separate meta‐analyses were conducted for each outcome and no study contributed twice to the same forest plot (except where the control group could be split for multi‐arm trials).

#### 4.3.3 Selection of studies

We included all studies that were identified as case management studies in the EGMs (White, 2018b; White, 2020a). Studies identified in the search that were not already included in the EGMs were screened against the inclusion criteria for eligibility by two independent screeners using EndNote at both title and abstract, and full text stage, with recourse to a third reviewer if there were any discrepancies.

#### 4.3.4 Data extraction and management

For all studies, we undertook dual data extraction, where two authors completed data extraction independently for each study. Coding was carried out by trained researchers. Any discrepancies in screening or coding were discussed between authors until a consensus was reached.

*Intervention studies*: An evidence table was designed, and piloted, for data extraction of intervention studies based on the coding framework developed by Keenan (Keenan, 2020).

We extracted the following data: publication details, intervention details including type of case‐management approach, design and type of research study, population characteristics (including age, gender, household type [individual/family]), any health information, sample sizes, attrition rates, data required for any meta‐analyses, time to follow up, descriptions of the outcomes of interest including instruments used to measure, quality assessment. See Supporting Information: Appendix 3 for summary data and https://github.com/MarkKelson/CHI_analysis for detailed information.

Specifically, we summarised:

1. The type of case management approach (Figure 1) and its components according to the following categories and (preliminary) measures of intensity:
  - Case manager continuity (Named case manager vs. No dedicated case manager)
  - Caseload of the case manager (defined as high ≥21; medium 8–20; light ≤7)
  - Frequency of contact with PEH (defined as very frequent ≥8 times/month; frequent 4–7 times/month; medium 2–3 times/month; occasional ≤once/month)
  - Availability of the support (defined as high 24/7; office hours (guaranteed response) or low < office hours)
  - Level of input PEH have in goal setting and care planning (case manager led or person led)
  - Time‐limit of provision of the support (defined as long term ≥3 years, medium >6 months to < 3 years, short term 3–6 months; very short term <3 months)
  - Location of appointments (institution, community, independent accommodation, remotely)
  - Degree of arranging service provision versus referral/coordinating arrangements to others
  - Team versus individual approach to case management
  - Types of case manager (non‐professional, with lived experience, professional)
  - Whether there are conditions attached to the support provided (Not conditional vs. conditional)
  - Knowledge regarding case management effectiveness in relation to the characteristics of the recipients of this intervention, which may include:

1. Type of case management approach
2. Complexity of needs
3. Age
4. Household type
5. Gender
6. Type of homelessness experienced
7. UK national versus non‐UK national
8. Ethnicity
9. Care or prison leaver
10. LGBTQI+
11. Whether first time or multiple homeless

Additional descriptive information for each of the studies was extracted and coded to allow for sensitivity and subgroup analysis. This included information regarding:

- Setting, which type of institutional setting(s) are study participants transitioning from?
- Demographic variables relating to the participants including age, complexity of needs,^3^ dependent children, and other relevant population characteristics.

Quantitative data was extracted to allow for calculation of effect sizes (such as mean change scores (analysed according to the Cochrane handbook section 9.4.5.2) and standard error or pre and post means and standard deviations or binary 2 × 2 tables). Data was extracted for the intervention and control group on the relevant outcomes measured to assess the intervention effects.

Multi‐arm trials with arms that are not comparing case management against usual care had just the relevant information extracted.

Where data were available, sensitivity/subgroup analyses were carried out for the primary outcome (homelessness) and the key secondary outcome (mental health) with regard to the intervention components.

Quantitative data extraction relied primarily on information reported in the eligible papers. Protocol or sister publications were explored to add additional information on relevant components. Information was taken from tables or figures. Where means and p‐values/t‐tests were reported, standard deviations were recovered assuming equal standard deviations between arms. The RevMan calculator was used to calculate standard deviations from standard errors. Multi‐arm studies had control groups split (uneven total sample sizes were rounded down). No standard deviations needed to be imputed.

*Implementation studies*: An evidence table was designed and piloted for the data extraction of the implementation (qualitative) studies. We extracted the following data: publication details, type of case‐management approach, design and type of research study, research question, theoretical approach adopted (if any), setting, participants, recruitment process, method of analysis, themes identified in relation to any of the case management components and recipient characteristics as outlined above, quality assessment. See Supporting Information: Appendix 4 for summary data.

As with the quantitative studies, data extraction from the implementation studies relied primarily on information reported in the eligible papers. Protocol or sister publications were explored to add additional information on relevant components.

#### 4.3.5 Assessment of risk of bias in included studies

Where studies had not already been assessed for risk of bias for inclusion in the Evidence and Gap Map (EGM), assessment of methodological quality and potential for bias was conducted using the methods used by the Campbell Collaboration for the EGM. Intervention papers were assessed with the tool published in White, 2019). Qualitative, process and implementation studies were assessed using a tool published in White (2018a). Assessments of risk of bias was carried out by two reviewers independently with discussion to resolve any differences.

We did not exclude studies based on our assessment of methodological limitations. We recorded this information in Summary of Findings Tables (Supporting Information: Appendix 3) to use it to assess our confidence in the review findings.

For studies that were not included in the EGM, data were shared with the Campbell Collaboration for inclusion in the Centre for Homelessness Impact's Homelessness Effectiveness Map: https://centreforhomelessnessimpact.github.io/egm/

#### 4.3.6 Measures of treatment effect

We expected most of outcome data to be binary (i.e., outcomes with only two possible values. An example would be assessing whether participants were homeless: yes/no). This turned out not to be the case, with more continuous outcome data available. Continuous variables are meaningful numbers (such as number of days spent homeless in the previous year).

Binary information for each group (treatment and comparator) at follow‐up was extracted directly from papers, or derived from reported percentages or sometimes extracted from a published graphic.

Continuous outcomes for each group (treatment and comparator) at follow‐up were directly extracted from papers, derived from reported standard errors or standard deviations, or extracted from published graphics.

Binary and continuous measures were combined by calculating a treatment effect and associated standard error for all studies and combining these using a generic inverse variable approach (metagen from the meta package for R).

#### 4.3.7 Unit of analysis issues

Where possible for studies involve group‐level allocation, data was included that had been adjusted to account for the effects of clustering, typically through the use of multilevel modelling or adjusting estimates using the intra‐cluster correlation coefficient (ICC). Where the effects of clustering had not been taken into account, estimates of effect size were adjusted following guidance in the Cochrane Handbook (section 23.1.4 using the effective sample size approach). Studies that employed cluster randomised had their standard errors inflated assuming an ICC of 0.05.

Some guiding principles were used in the data extraction. If self‐report and externally assessed measures were used for an outcome we used self‐report measures to better capture participants lived experience. For drug misuse outcomes if alcohol and other drug outcomes were reported we used the alcohol ones as alcohol seemed more prevalent. If multiple mental health outcomes were reported, then the most common conditions (such as anxiety or depression) were selected. When multiple time points were reported we selected the longest follow‐up strictly less than 1 year and the longest overall.

#### 4.3.8 Dealing with missing data

If study reports did not contain sufficient data to allow calculation of effect size estimates we employed standard methods to calculate a SMD from reported statistics or graphics in the paper (Rosnow, 1996; Rosnow, 2000). We used R and RevMan to do this (Balduzzi, 2019). Where no information was forthcoming the study was not included in meta‐analysis but in a narrative synthesis.

#### 4.3.9 Assessment of heterogeneity

Heterogeneity was assessed through visual inspection of the forest plot and checking for overlap of CIs and second through the *I* ^2^ and *τ* ^2^ statistics.

#### 4.3.10 Assessment of reporting biases

A funnel plot and Egger's linear regression test were included to check for publication bias across included studies (Sterne, 2006).

Funnel plots explore publication bias where more than ten studies are included. A funnel plot presents the effect sizes for individual studies on the *x*‐axis (standardised for comparability), against an increasing measure of study precision on the *y*‐axis. As precision increases for a study, we expect that the observed treatment effect will converge towards the overall average (i.e., that is, more precise studies are expected to get closer to the true underlying treatment effect).

This creates an inverted cone on the funnel plot, where we expect most of the studies to lie. Asymmetry in this plot indicates that some subgroup of studies is missing (e.g., perhaps small studies with small effects are systematically failing to reach the publication record). A formal test of asymmetry in this setting involves running a linear regression and testing whether the intercept goes through the origin (Egger, 1997).

#### 4.3.11 Data synthesis

All statistical analyses were conducted using the R program using the ‘meta’ library (Balduzzi, 2019). A random‐effects analysis (REM) with independent effects (with single effect sizes per study) was used. This decision to employ a REM is made for two reasons. First, we expect studies to vary substantially in terms of population served, training of case managers, outcomes assessed, and study designs.

The random effects approach assumes that the true treatment effects associated with each study are drawn from a distribution of possible effects. They are similar, but not identical. In contrast, the fixed effects approach assumes that all of the studies are imperfect estimates of the exact same fixed effect estimate (i.e., the same number). Random effects models are more appropriate given the heterogeneity of studies (they all implement different interventions, have different inclusion criteria, durations, outcomes, etc.), but fixed effects estimates are also provided for information.

Meta‐analysis was conducted to test effectiveness of interventions to improve case‐management approaches across various domains relating to homelessness. The outcomes related to homelessness are both binary and continuous and so the effect size metrics chosen were relative risks and SMDs.

Continuous outcomes were meta‐analysed in two ways: first, when summary information on the separate randomised groups was available, it was analysed using random effects continuous meta‐analysis (Balduzzi, 2019). Second, a small number of studies reported between group effect estimates only. Equivalent between group effect estimates were calculated for the studies that reported information on the separate randomised groups and all these effect estimates were meta‐analysed using generic inverse variance methods.

Binary outcomes were analysed using the Mantel‐Haenszel method implemented in the metabin function in the meta package (Balduzzi, 2019). This was a pre‐specified analysis approach and is supported by the Cochrane Handbook section 10.4.1.

#### 4.3.12 Subgroup analysis and investigation of heterogeneity

We conducted a number of subgroup analyses, where sufficient data were available, to explore whether study, intervention or sample characteristics influenced the overall effect of the intervention on each outcome. Forest plots with separate summaries for groups of studies are presented. Formal tests between groups were obtained using the metareg function from the meta package (using the Hartung‐Knapp method). The moderating variables included:

- the methodological quality of the study (study design/risk of bias assessment),
- the age of participants,
- the gender of participants,
- the ethnicity of participants
- type of homelessness (according to the FEANTSA classification; FEANTSA 2017),
- whether the intervention was aimed at single people or families,
- setting of the intervention,
- how the intervention was classified (according to the framework discussed earlier) as aiming to increase access to services through improving the availability, acceptability or affordability of the programme,
- the intervention components (see Analysis of Findings).

We were particularly interested in teasing apart the contributions of different intervention components to outcomes. Where sufficient studies were identified (at least 10) we included intervention component information (either continuously or categorically measured) for the intervention components listed in the Analysis of Findings in a meta‐regression. Bubble plots and regression coefficients and their 95% CIs were used to summarise the results.

#### 4.3.13 Sensitivity analysis

Where feasible, for the primary outcomes, sensitivity analyses were carried out on the basis of study design and risk of bias assessment.

##### Summary of findings and assessment of the certainty of the evidence

Formal subgroup analysis within the meta‐analyses was planned in relation to the quality of individual studies but relatively few high‐quality studies were identified (see Risk of Bias in Included Studies). No formal subgroup analysis was done based on risk of bias and no formal summary of findings tables were completed. The overall strength of evidence was not high and conclusions, where drawn from the intervention and implementation research, were based on the consistency of findings across a body of evidence.

###### Data synthesis of implementation studies

We described the characteristics of included studies in terms of the methods used to capture data on the factors that may impact on intervention implementation and success; the number of interviews/focus groups/observations that have taken place, who participated and the nature of qualitative data collection (type and time taken).

As with Keenan (2020) the framework synthesis approach was adopted, supported by the use of NVivo.

This Framework synthesis comprised five overlapping methodological stages:

1. Familiarisation—With issues and ideas around the topic by an initial screening of relevant studies identified in the search.
2. Framework Selection—To agree the conceptual framework or logic model to provide a potential set of themes or concepts that may affect implementation success.
3. Indexing—To data extract information from each study in relation to their main characteristics and findings.
4. Charting—To group the study findings in relation to the themes in the Framework and any new themes/sub‐themes derived directly from the inductive data‐driven process.
5. Mapping and Interpretation—The derived themes were considered in light of the interventional research and its components.

At the familiarisation and framework selection stages, authors considered using the framework of factors influencing implementation adopted within the relevant Campbell Evidence and Gap Map (White, 2018a). However, this did not encompass all the themes identified within this body of implementation research. Thus the synthesis was driven by the evidence and new themes were added into the framework as appropriate.

At the charting stage, given the very large amount of relevant evidence (191 papers), purposive sampling (Booth, 2016) was employed to include research spanning geography, targeted populations and types of intervention to exhibit an accurate representation of the case management programmes available with the prioritisation of high quality studies (i.e., those with a formal qualitative methodology). The selected process evaluations should present the most ‘rich’ (in theory) and/or ‘thick’ (in context) data (Booth, 2016) from the studies included.

Through discussion within the review team and with subject experts, the following process was agreed for the purposive sample:

- All qualitative studies associated with the included intervention studies—ACCESS (ICM), HOP‐C (CTI), HPACT (ACT), Pathways to Independence (SCM), Skid Row (SCM).
- A sample of qualitative studies representing the range of case management interventions from Housing First/Chez Soi/HUD‐VASH (including any comparisons of the ICM and ACT approaches within Housing First) in which the study was exploring one or more of the case management components.
- A sample of studies spanning the four case management types—ICM, ACT, SCM, CTI—where the paper was exploring one or more of the components of the case management approach. No study of Brokered Case Management was identified.

This resulted in a final data set of 41 included studies, providing extensive data for analysis. The majority of studies (*N* = 37) adopted a formal qualitative approach though, to ensure even coverage across the case management types and components, three analysed survey data and one was a secondary analysis of quasi‐experimental data (see Supporting Information: Appendix 4 for details). Overall 12 of the studies explored Housing First (including the Chez Soi and HUD‐VASH iterations of Housing First), 6 explored other types of ICM, 5 explored other types of ACT, 6 explored SCM, 4 explored CTI and, for the remaining 8 studies, the case management type was mixed or unclear.

*Reflexivity*: Review author reflexivity, that is, the potential for pre‐existing views to influence review conclusions was considered at all stages of the review. Subject experts in the review team considered any views and positions that might influence the review's conclusions to mitigate any potential for bias. The majority of the data extraction, critical appraisal and analysis was carried out by review methodologists rather than subject experts.

#### 4.3.14 Synthesis of findings across the intervention and implementation studies

The overall synthesis of findings across this mixed methods review was guided by the method proposed by Harden (Harden, 2018) for integrating contextual features from the qualitative research with findings from the effectiveness studies.

At the final mapping and interpretation stage, the team, with its subject experts, collaborated closely with CHI as well as the panel of experts they convened who considered these themes in light of the available empirical literature. For the interventions available for meta‐analysis, implementation evidence directly linked to these interventions, and any evidence in relation to component interventions, was considered in light of specific adjustments to the interventions that might be considered.

Overall, the aim of this synthesis was to guide practice, to help policy makers design interventions, and researchers to prioritise parameters that should be tested more rigorously.

## 5 RESULTS

### 5.1 Description of studies

#### 5.1.1 Results of the search

After deduplication, 4157 records were identified from the database searching and Campbell Evidence and Gap Maps and a further 22 records were assessed as potentially relevant from the web site searching and reference list checking of included papers and any systematic reviews identified.

**Figure 3:**
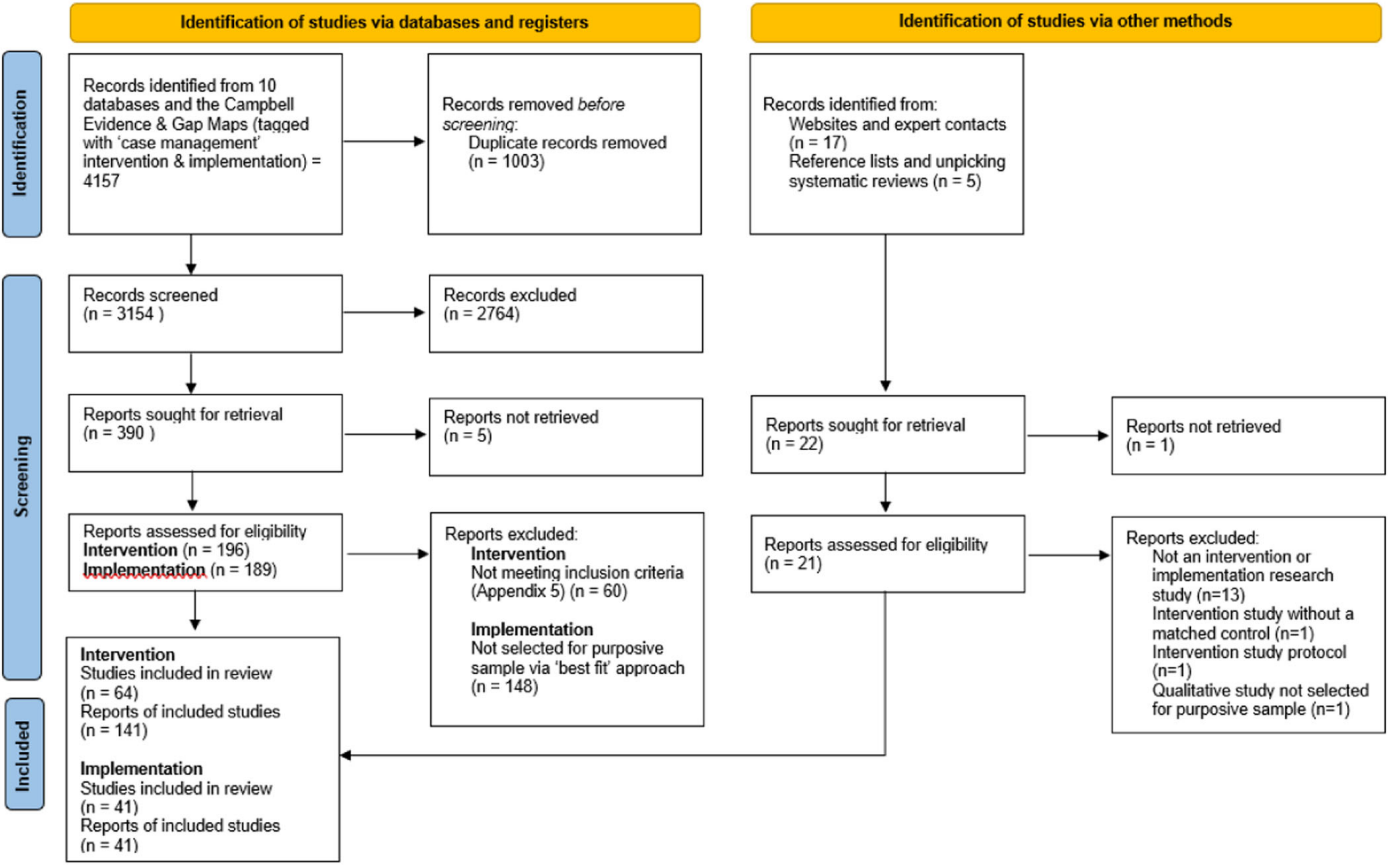
Flow diagram.

A flow diagram to describe inclusions and exclusions at each stage of the search and screening process is provided in Figure 3.

#### 5.1.2 Included studies

A total of 105 studies met the inclusion criteria for the review. These comprised 64 studies looking at case management interventions for PEH (from 141 papers, with many papers covering a single study; eg. At Home/Chez Soi and Vancouver At Home). A purposive sample of 41 studies (41 papers) of qualitative and other ‘views’ studies was also analysed to explore factors that might affect the implementation (likely success) of a case management intervention. See Figure 3.

Multiple reports of many of the intervention studies was noted and checking of their reference lists did not identify any additional studies for inclusion. All the detailed information regarding the data extraction of the intervention (quantitative) papers is available at https://github.com/MarkKelson/CHI_analysis.

Of the 64 intervention studies, 48 were randomised controlled trials and 16 were non‐randomised controlled trials where adjustment was used to control for any baseline differences. The vast majority (53) of the studies took place in the USA, 3 in Canada (including the very large At Home/Chez Soi study), 3 in the UK, 2 in the Netherlands and one each in Australia, Spain and France.

Of the purposive sample of 41 implementation studies, 31 were qualitative in design, 4 were quantitative (e.g., cross‐sectional survey) and 6 were mixed methods with qualitative and quantitative components. As with the interventional research, the majority of studies (30) took place in the USA, 8 in Canada and one each in Australia, the Netherlands and the UK.

A summary of all included intervention studies is provided in Supporting Information: Appendix 3 and a summary of the implementation studies is provided in Supporting Information: Appendix 4.

#### 5.1.3 Excluded studies

Inclusion/exclusion criteria for the review are summarised in Supporting Information: Appendix 1.

The 59 excluded intervention studies are summarised in Supporting Information: Appendix 5. Details of the 148 implementation papers not selected for the purposive sample are available from the study authors.

### 5.2 Risk of bias in included studies

Intervention studies:

Findings from the critical appraisal of the 64 studies are provided in Figure 4. Looking at quality on a study‐by‐study basis, 27% of the studies (17/64) were assessed as high quality, 17% (11/64) as medium quality and 56% (36/64) as low quality.

Figure 4Critical appraisal of the intervention studies.

**Figure:**
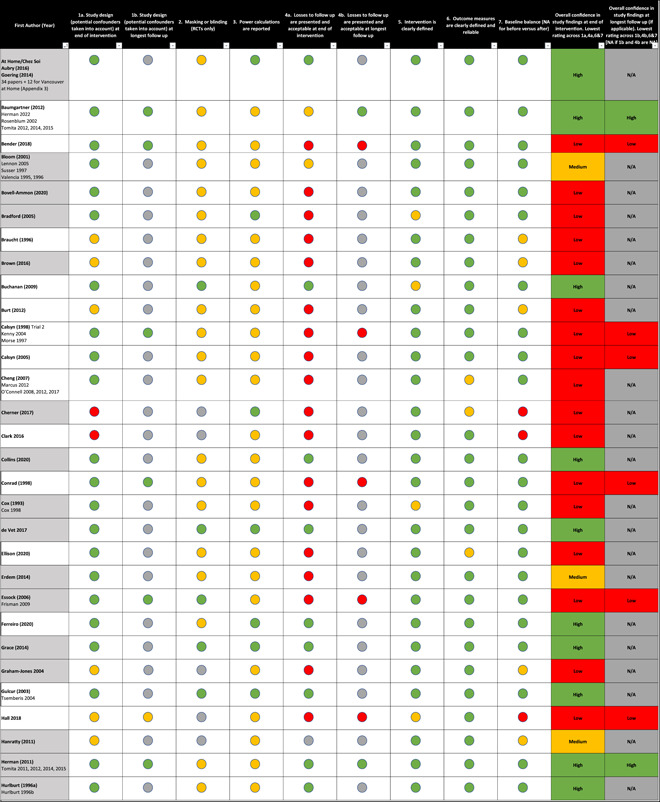

**Figure:**
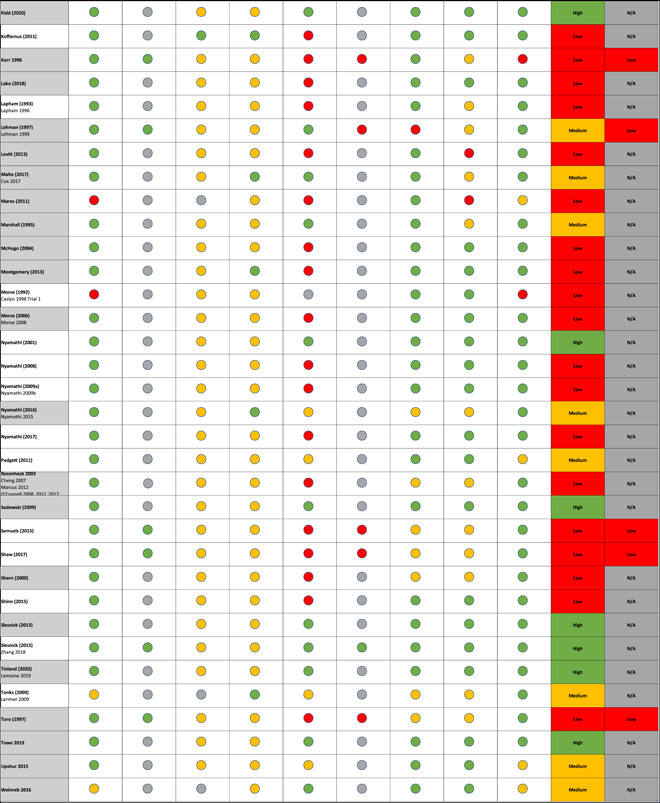

As can be seen from the data in Figure 4, many studies of otherwise high quality did not provide information supporting the contention that losses to follow up were acceptable at the end of the intervention leading to a risk of attrition bias, due to loss of outcome data. This resulted in an assessment of a low‐quality study based on the critical appraisal tool guidance (White, 2019).

**Figure 5:**
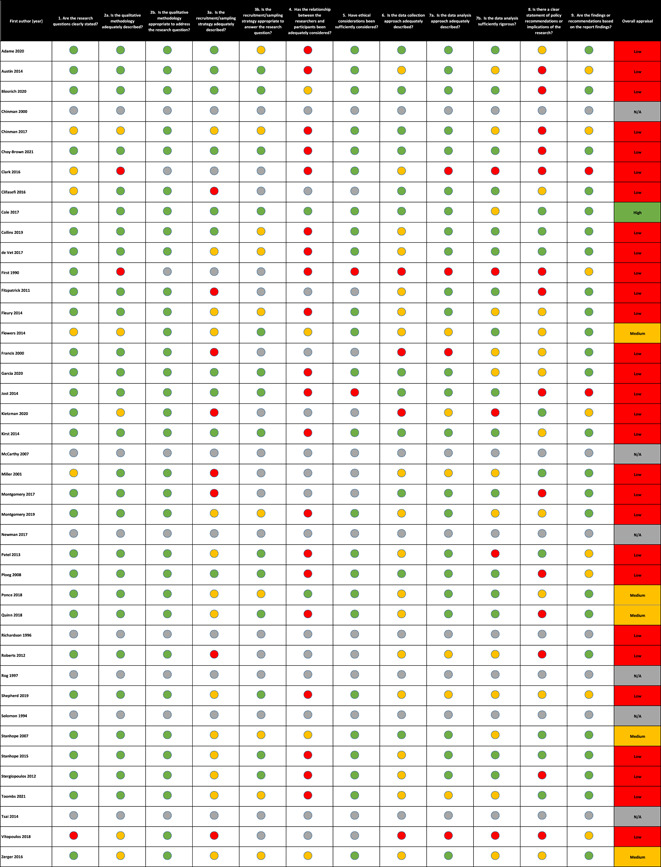
Critical appraisal of the implementation studies.

Implementation studies:

Data from the critical appraisal of the 41 implementation studies are provided in Figure 5. 12% (5/41) studies were judged as high quality, 2% (1/41) as medium quality and 71% (29/41) as low quality. As seen in Figure 5, many studies of otherwise high quality did not describe the relationship between the researchers and participants (reflexivity) and any steps taken to mitigate any potential for researcher bias. The absence of this discussion led to studies being assessed as low quality based on the critical appraisal tool guidance. The remaining six studies (15%) were not of a qualitative research design and thus it was not appropriate to appraise them according to the Campbell critical appraisal tool (White, 2018a).

**Figure 6:**
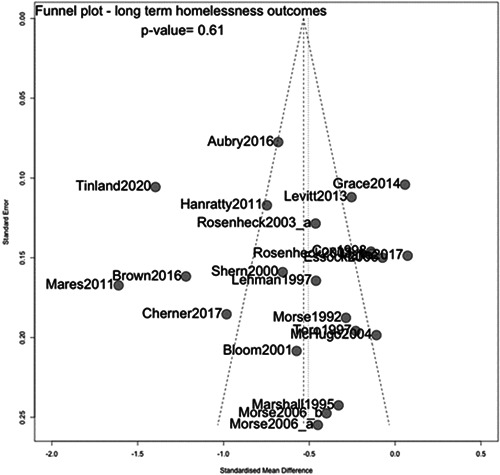
Funnel plot for homelessness outcomes.

Based on the Funnel plot (see assessment of reporting biases) there is no evidence of publication bias for the homelessness outcome, with most of the studies symmetrically distributed within the funnel. The formal test for asymmetry in this plot indicated a *p* value of 0.61 (Figure 6).

### 5.3 Effects of interventions

#### 5.3.1 Homelessness outcomes

**Figure 7:**
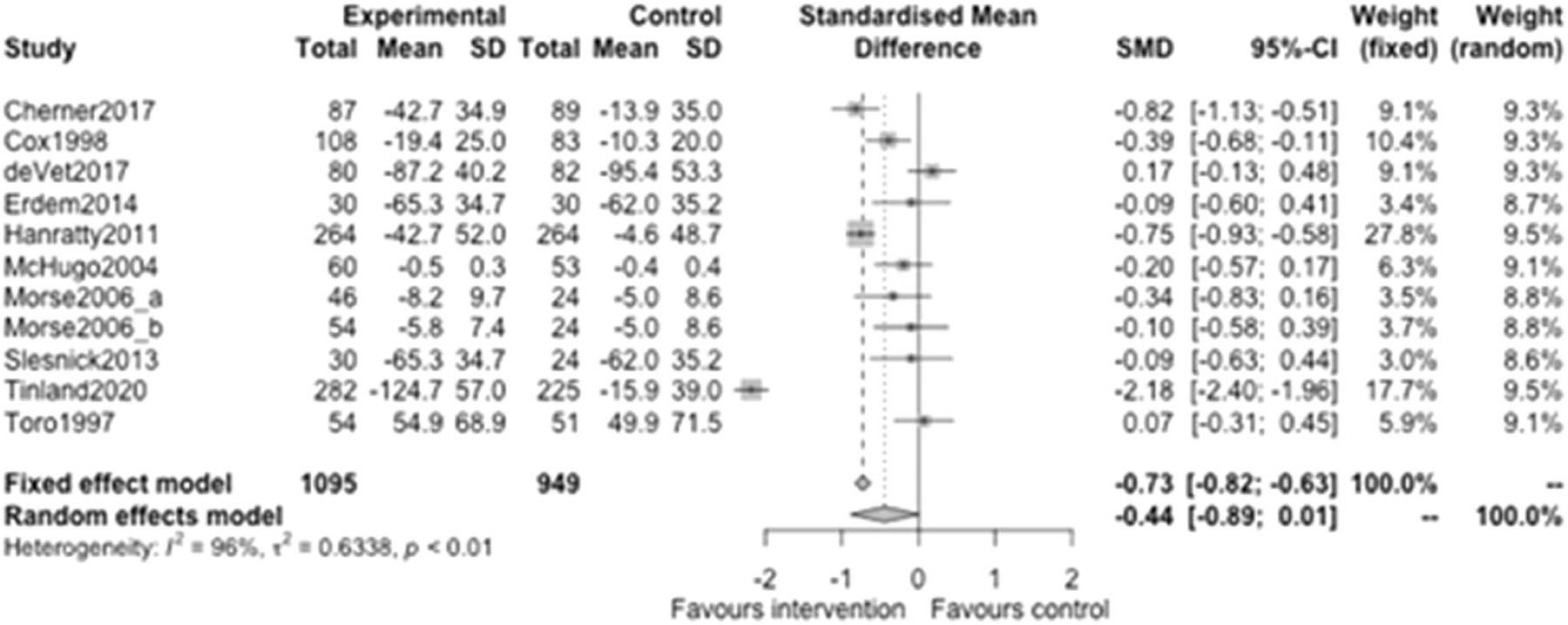
Homelessness outcomes less than 1 year.

Figure 7 shows that the overall estimate of effectiveness was −0.44, favouring case management, but was not statistically significant (*p* value = 0.054). This means that case management of any type improves short term (<12 months) homelessness outcomes by about half of a standard deviation (estimate: −0.44) (while this was not statistically significant, the CI excludes estimates of substantial detrimental effects on homelessness (95% CI: [−0.89, 0.01]). Variation between outcomes in the included studies was high as indicated by the high *I* ^2^ statistic (=0.96). Despite this high heterogeneity, most of the individual study treatment effects favour intervention providing some confidence in this finding shows that the overall estimate of effectiveness was −0.44, favouring case management, but was not statistically significant (*p* value = 0.054). This means that case management of any type improves short term (<12 months) homelessness outcomes by about half of a standard deviation (estimate: −0.44) (while this was not statistically significant, the CI excludes estimates of substantial detrimental effects on homelessness (95% CI: [−0.89, 0.01]). Variation between outcomes in the included studies was high as indicated by the high *I* ^2^ statistic (=0.96). Despite this high heterogeneity, most of the individual study treatment effects favour intervention providing some confidence in this finding.

**Figure 8:**
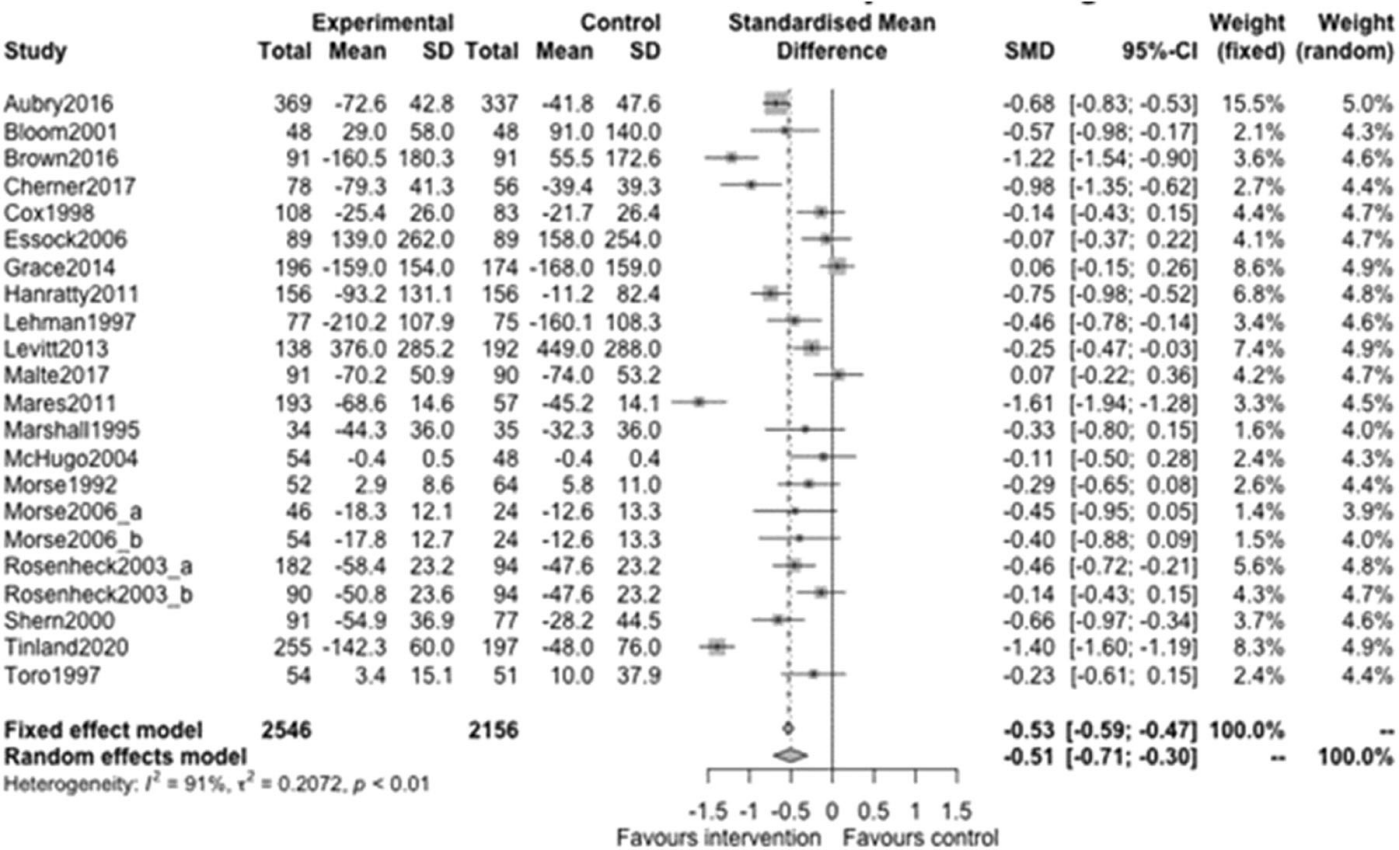
Homelessness outcomes 1 year or longer.

Figure 8 shows that the overall estimate of effectiveness was −0.51, favouring case management, and was statistically significant (*p* value: <0.01). This means that case management of any type improves long term (12 months or longer) homelessness outcomes by about half of a standard deviation (estimate: −0.51, 95% CI: [−0.71, −0.3]). Heterogeneity was high (=0.91). Again, despite this high heterogeneity, the studies consistently favour intervention.

**Figure 9:**
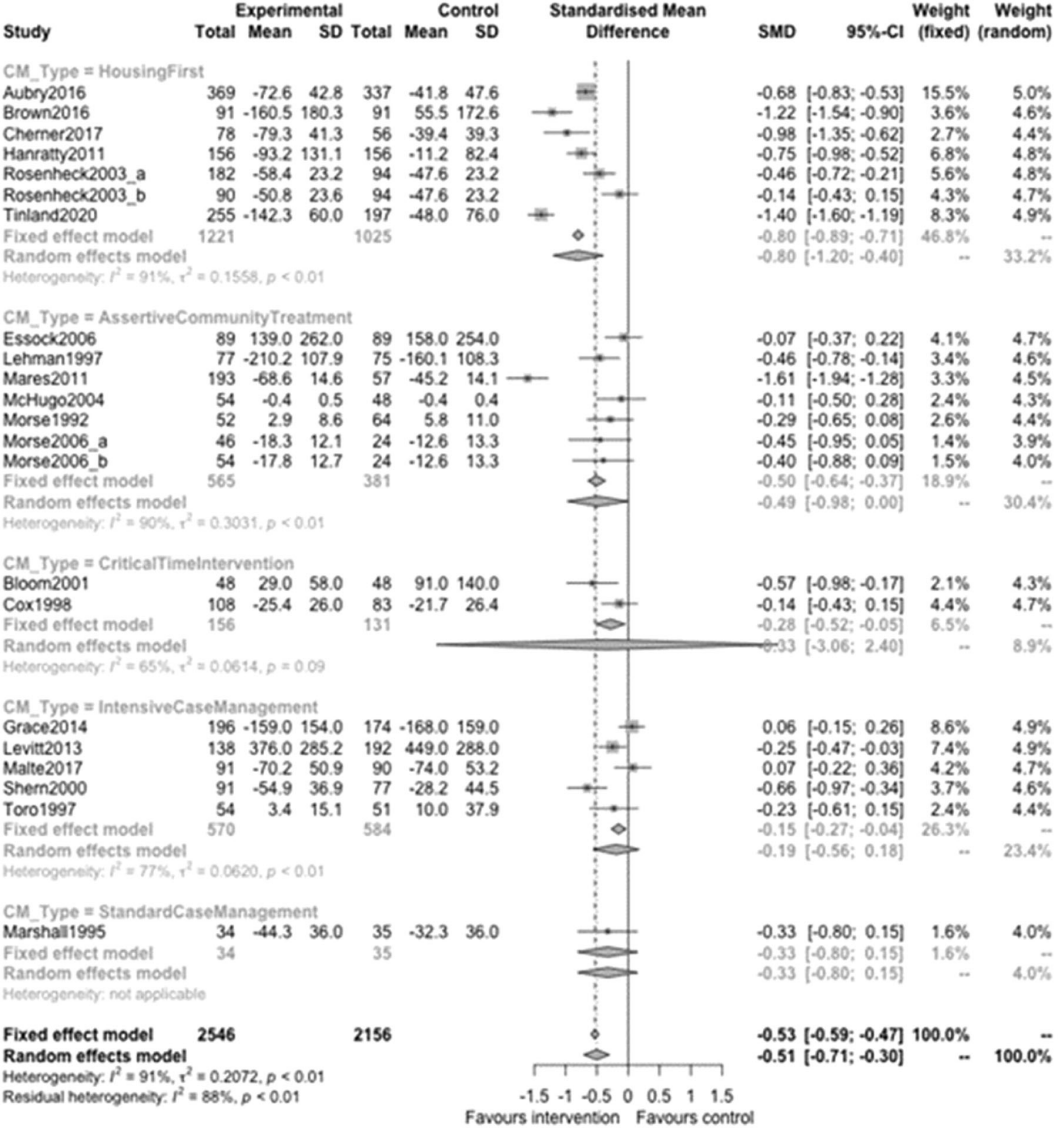
Homelessness outcomes 1 year or longer split by case management type.

Continuous outcomes with longer follow‐up had enough studies included to allow us to explore types and components of case management interventions. Figure 9 shows that case management subgroup effectiveness was −0.8 (95% CI: [−1.2,−0.4]) for Housing First, −0.49 (95% CI: [−0.98, 0]) for Assertive Community Treatment, −0.33 (95% CI: [−3.06, 2.4]) for Critical Time Intervention and −0.19 (95% CI: [−0.56, 0.18]) for Intensive Case management. These effects are all expressed in standard deviations of the chosen outcome variable. The outcome for Aubry (2016) for example was the percentage of time housed in the previous 3 months and both arms had observed standard deviations of about 45%. For such an outcome, the average effect size (for the Housing First subgroup) was 80% of 45% (equating to a reduction of 36% in time spent homeless). The only statistically significant difference was between the largest and smallest of these effects (Housing First vs. Intensive Case Management, *p* value = 0.03).

**Figure 10:**
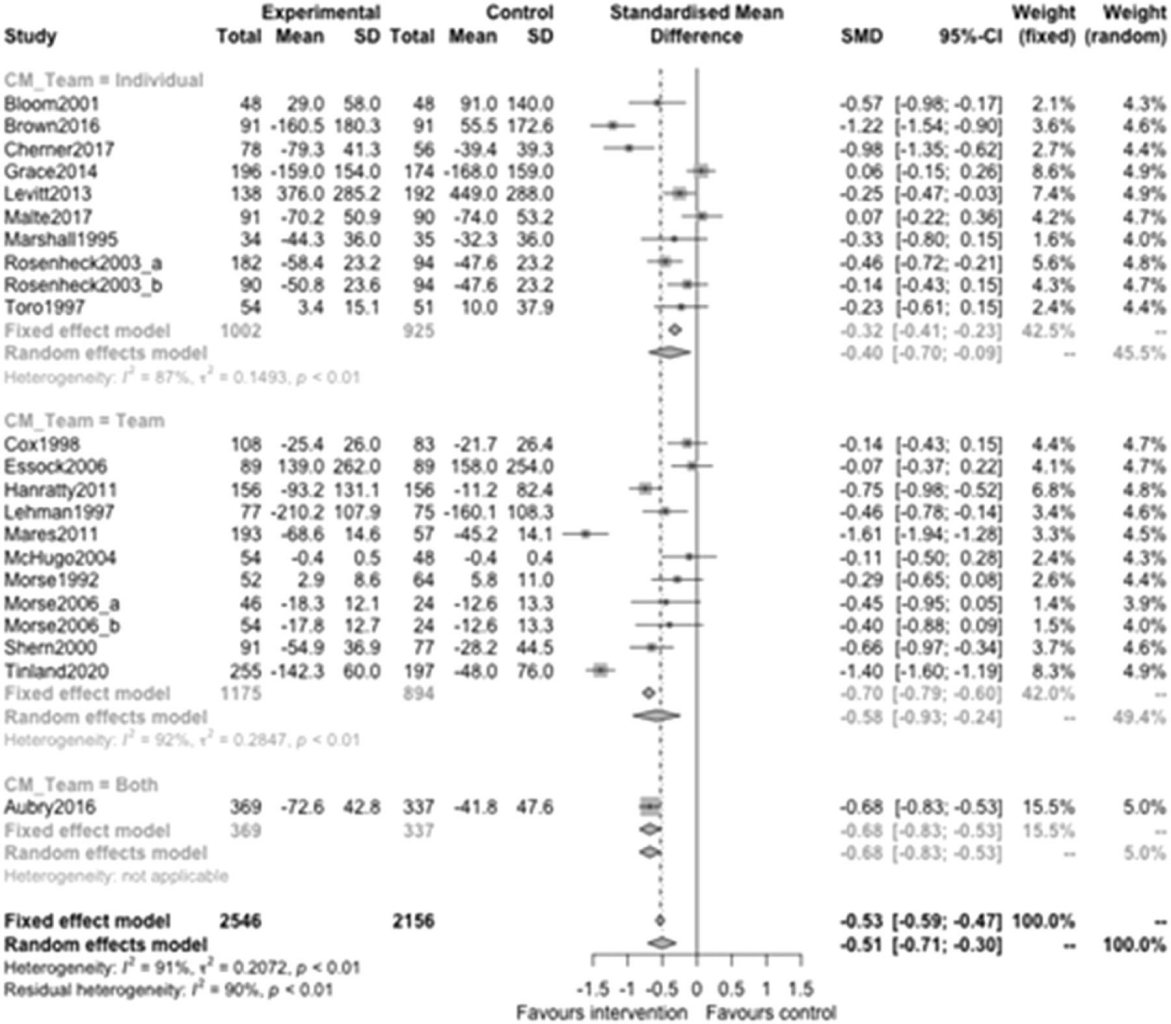
Homelessness outcomes 1 year or longer split by team‐individual.

Figure 10 shows that team or individual provision subgroup effectiveness was −0.58 (95% CI: [−0.93, −0.24]) for Individual, −0.68 (95% CI: [−0.83, −0.53]) for Team. There is no evidence of a difference between these effects (estimate: −0.19, 95% CI: [−0.62, 0.25], *p* value = 0.38). There was a large subgroup of studies which did not report this information (this was consistently observed for most of the components we explored).

**Figure 11:**
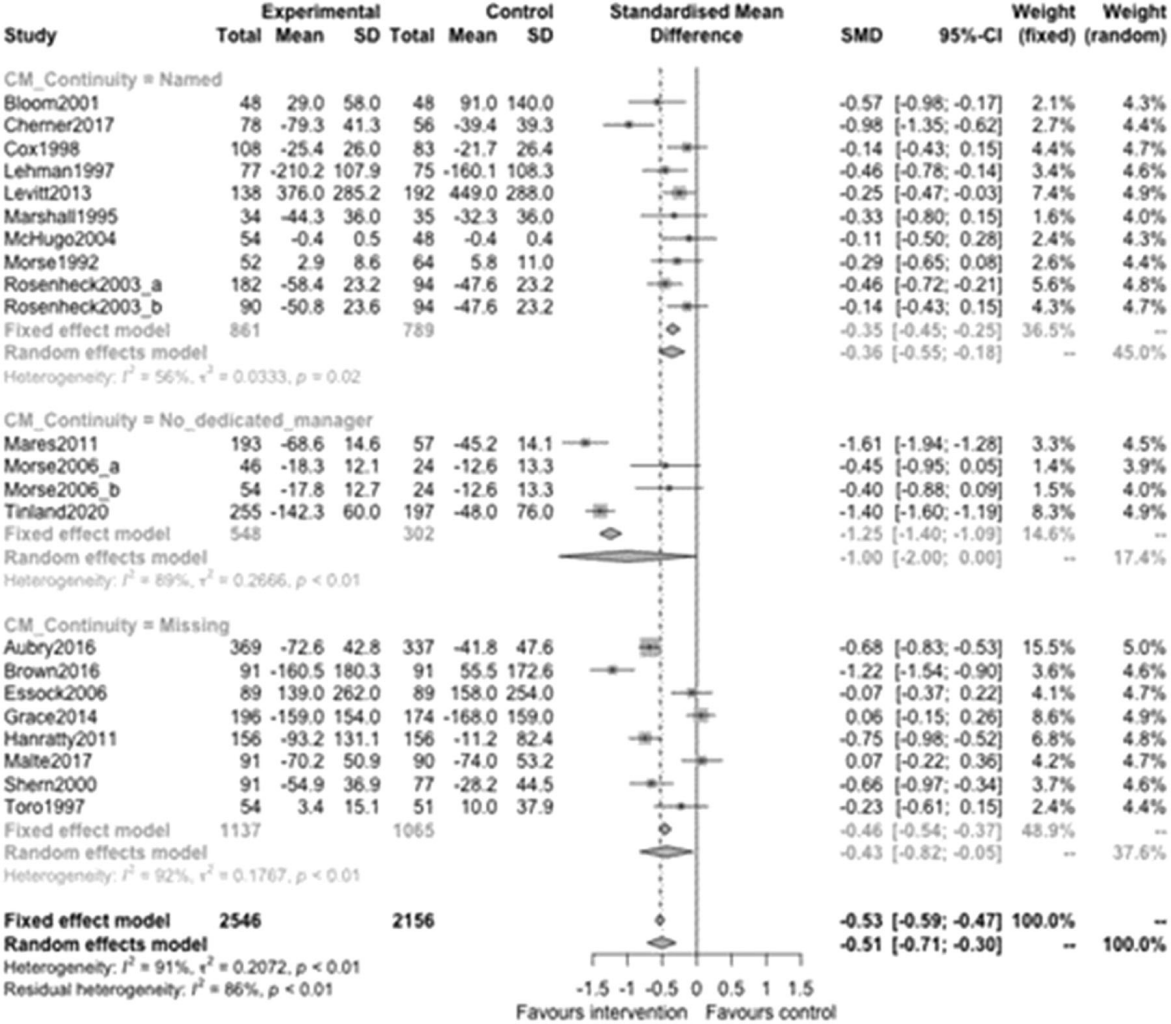
Homelessness outcomes 1 year or longer split by continuity.

Figure 11 shows that continuity of care subgroup effectiveness was −0.36 (95% CI: [−0.55, −0.18]) for a named case manager, and −1.00 (95% CI: [−2.00, 0.00]) for when there was no dedicated case manager. The CIs are largely overlapping for these subgroups however there is a statistically significant difference between them (estimate: −0.66, *p* value: 0.02, 95% CI: [−1.19, −0.13]).

**Figure 12:**
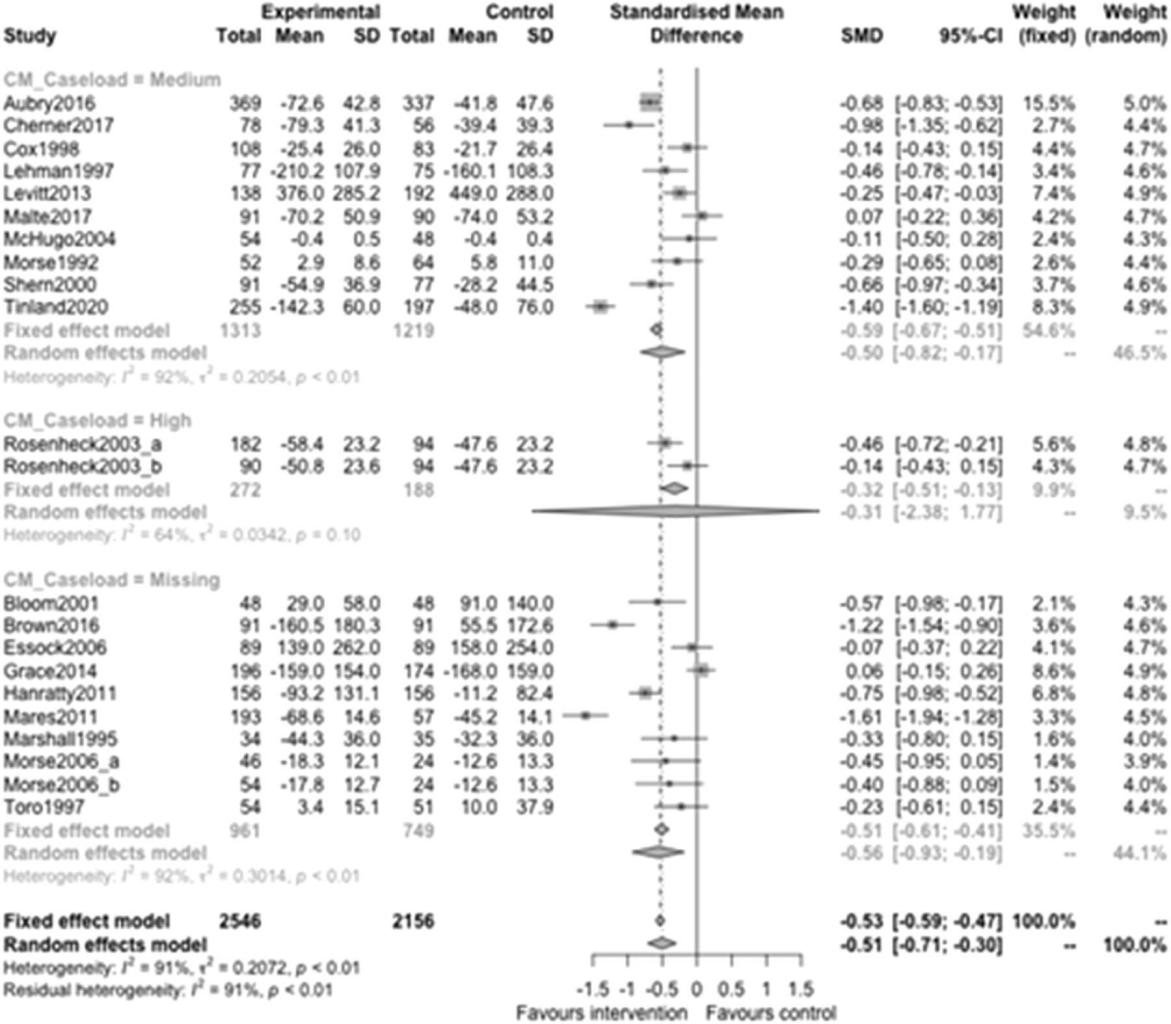
Homelessness outcomes 1 year or longer split by caseload.

Figure 12 shows that caseload subgroup effectiveness was −0.5 (95% CI: [−0.82, −0.17]) for medium caseloads (8–20 PEH per case manager), and −0.31 (95% CI: [−2.38, 1.77]) for high case loads (21 or more PEH per case manager). The CIs are wide and overlapping indicating that there is little evidence of a difference between these subgroups (estimate: 0.19, *p* value: 0.6, 95% CI:[−0.57, 0.96]).

**Figure 13:**
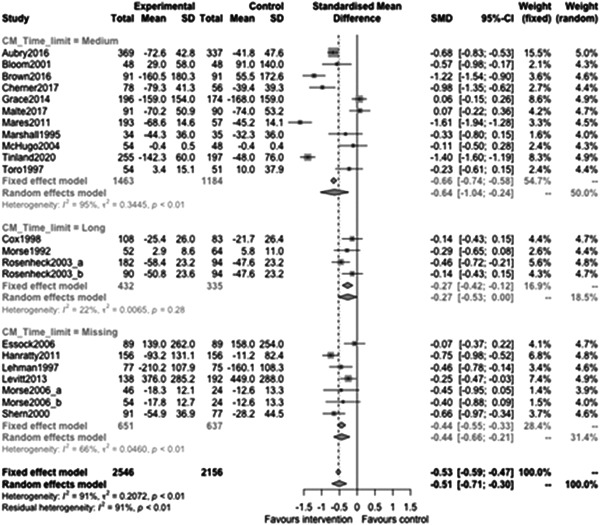
Homelessness outcomes 1 year or longer split by time limit.

Figure 13 shows that time limit of provision subgroup effectiveness was −0.64 (95% CI: [−1.04, −0.24]) for medium time limits (more than 6 months but less than 3 years), and −0.27 (95% CI: [−0.53, 0]) for long time limits (3 years or longer). This was not a statistically significant difference (*p* value = 0.16).

**Figure 14:**
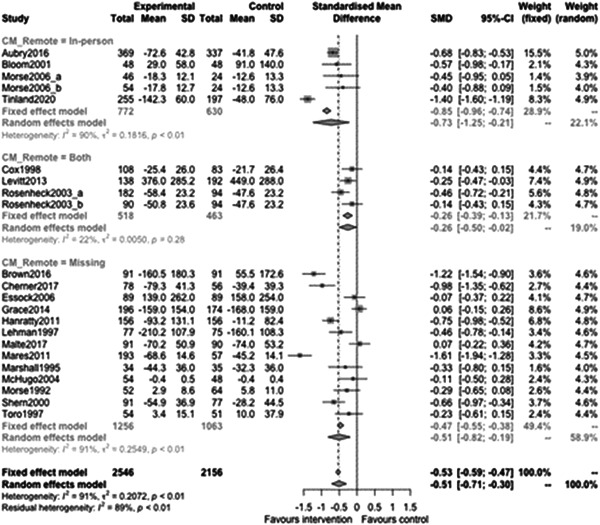
Homelessness outcomes 1 year or longer split by remote or in‐person.

Figure 14 shows that remote/in‐person subgroup effectiveness was −0.73 (95% CI: [−1.25, −0.21]) for in‐person provision, and −0.26 (95% CI: [−0.5, −0.02]) for when both in‐person and remote were both used. Both of these subgroups demonstrated statistically significant differences from zero (indicating both models were effective at reducing homelessness) however the formal comparison between remote and mixed (‘Both’) provision was not statistically significant (estimate: 0.48, *p* value: 0.13, 95% CI: [−0.16, 1.12]).

**Figure 15:**
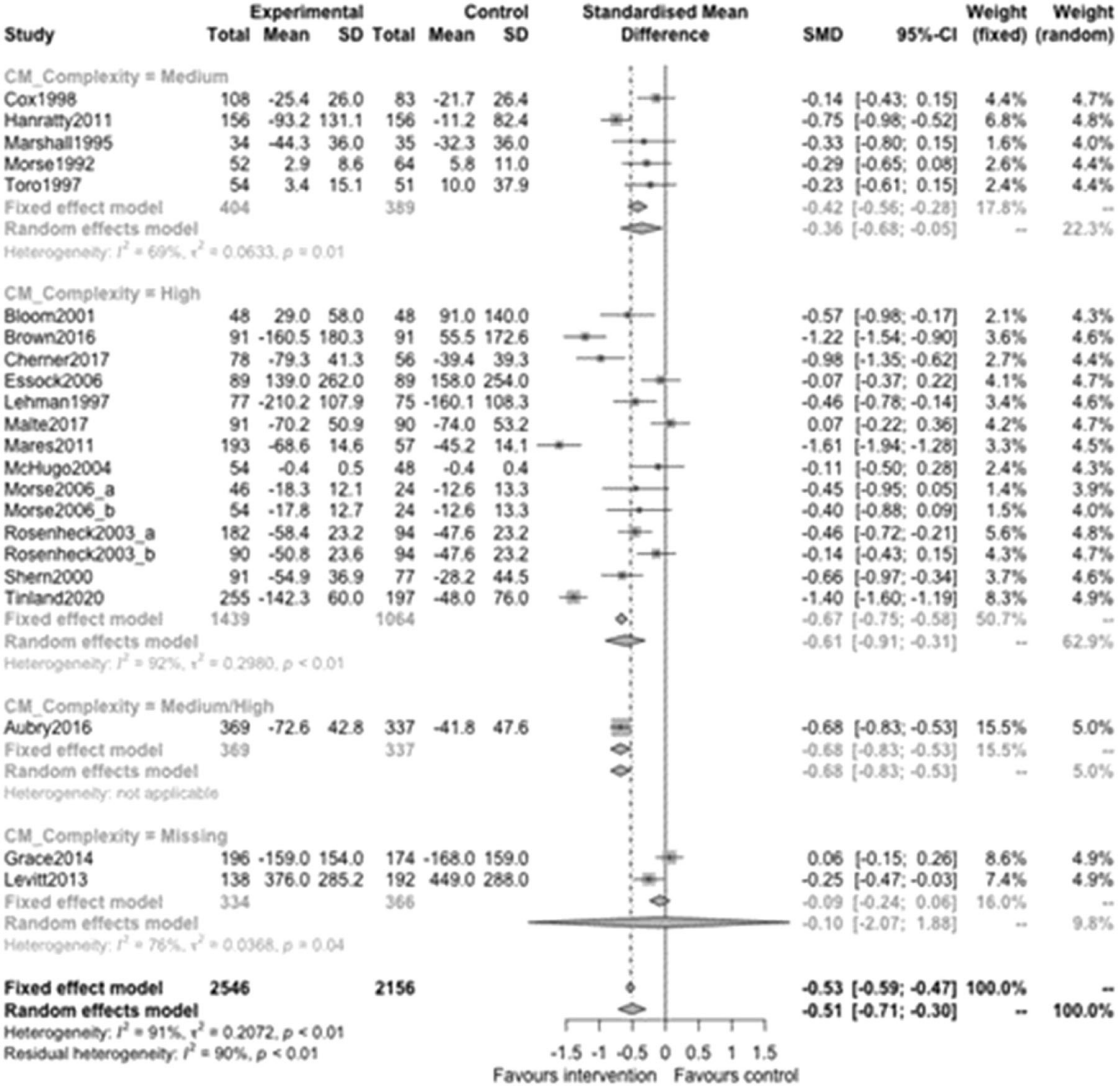
Homelessness outcomes 1 year or longer split by complexity of need.

Figure 15 shows that complexity of need subgroup effectiveness was −0.36 (95% CI: [−0.68, −0.05]) for medium complexity, and −0.61 (95% CI: [−0.91, −0.31]) for high complexity of need. There was not a statistically significant difference between these subgroups (estimate: −0.26, *p* value: 0.3, 95% CI: [−0.76, 0.25]).

**Figure 16:**
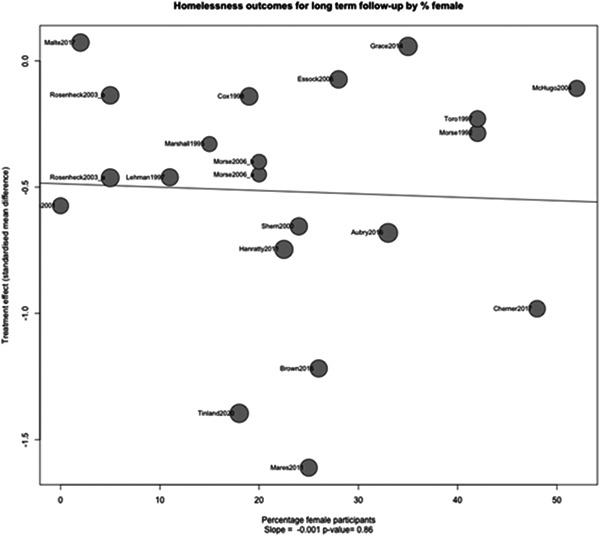
Meta‐regression of homelessness outcomes 1 year or longer split by % female participants.

Figure 16 shows that there is no evidence of a relationship between homelessness outcomes and the percentage of female participants. The slope of the line of the relationship is extremely shallow (slope: −0.001, *p* value: 0.86). This indicates that there is no evidence that case management is differentially effective for homelessness outcomes when the proportion of female participants is higher or lower.

**Figure 17:**
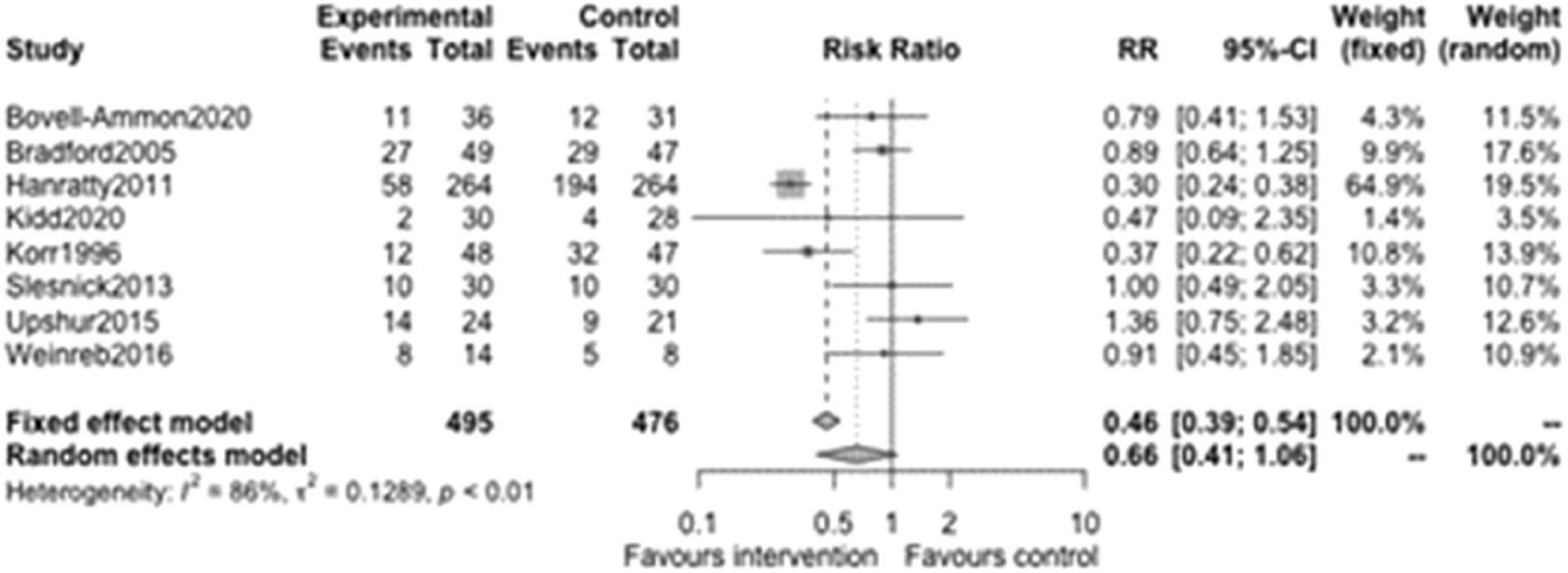
Binary homelessness outcomes of less than 1 year.

Binary outcomes (i.e., when the outcome is expressed as a yes/no type of response) were explored for homelessness. Figure 17 supports the previous continuous analyses of homelessness outcomes and shows that case management of any type is associated with a reduced odds of homelessness at follow‐ups less than 12 months (odds ratio [OR]: 0.66, 95% CI: [0.41, 1.06] albeit not statistically significantly so (*p* value = 0.08). This means that the point estimate was that trial participants receiving case management were about 34% less likely to experience follow‐up, however we are not confident that effect was not in fact detrimental to homelessness.

**Figure 18:**
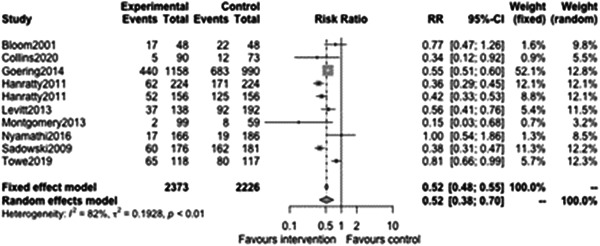
Binary homelessness outcomes 1 year or longer.

Figure 18 supports the previous continuous analyses of homelessness outcomes and shows that case management of any type is associated with a reduced odds of homelessness at follow‐ups less than 12 months (OR: 0.52, 95% CI: [0.38, 0.7]) which was statistically significant (*p* value = <0.01). This means that participants receiving case management typically had 48% reduced odds of being homeless at follow‐up.

**Figure 19:**
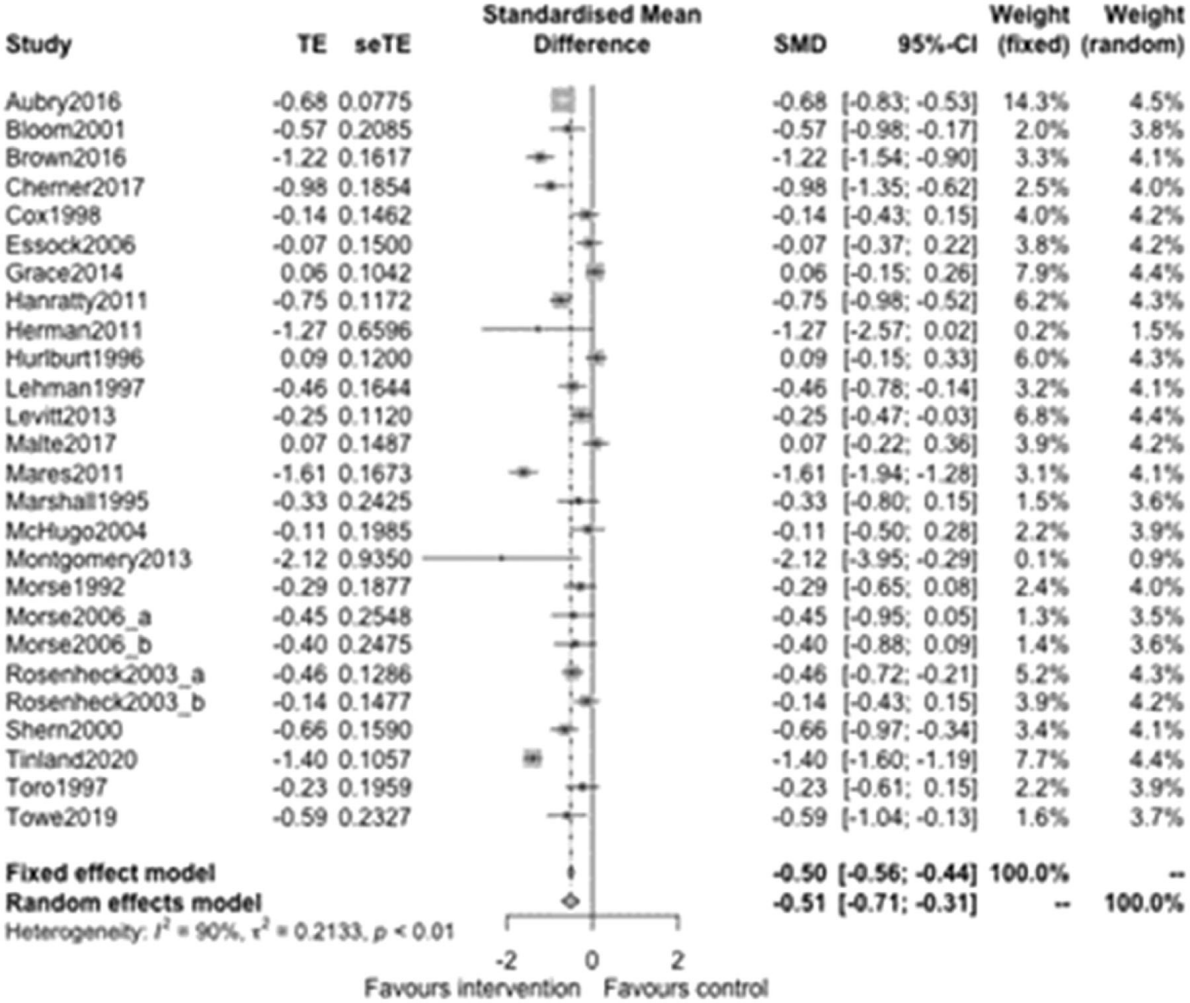
Generic inverse variance analysis of homelessness outcomes 1 year or longer.

**Figure 20:**
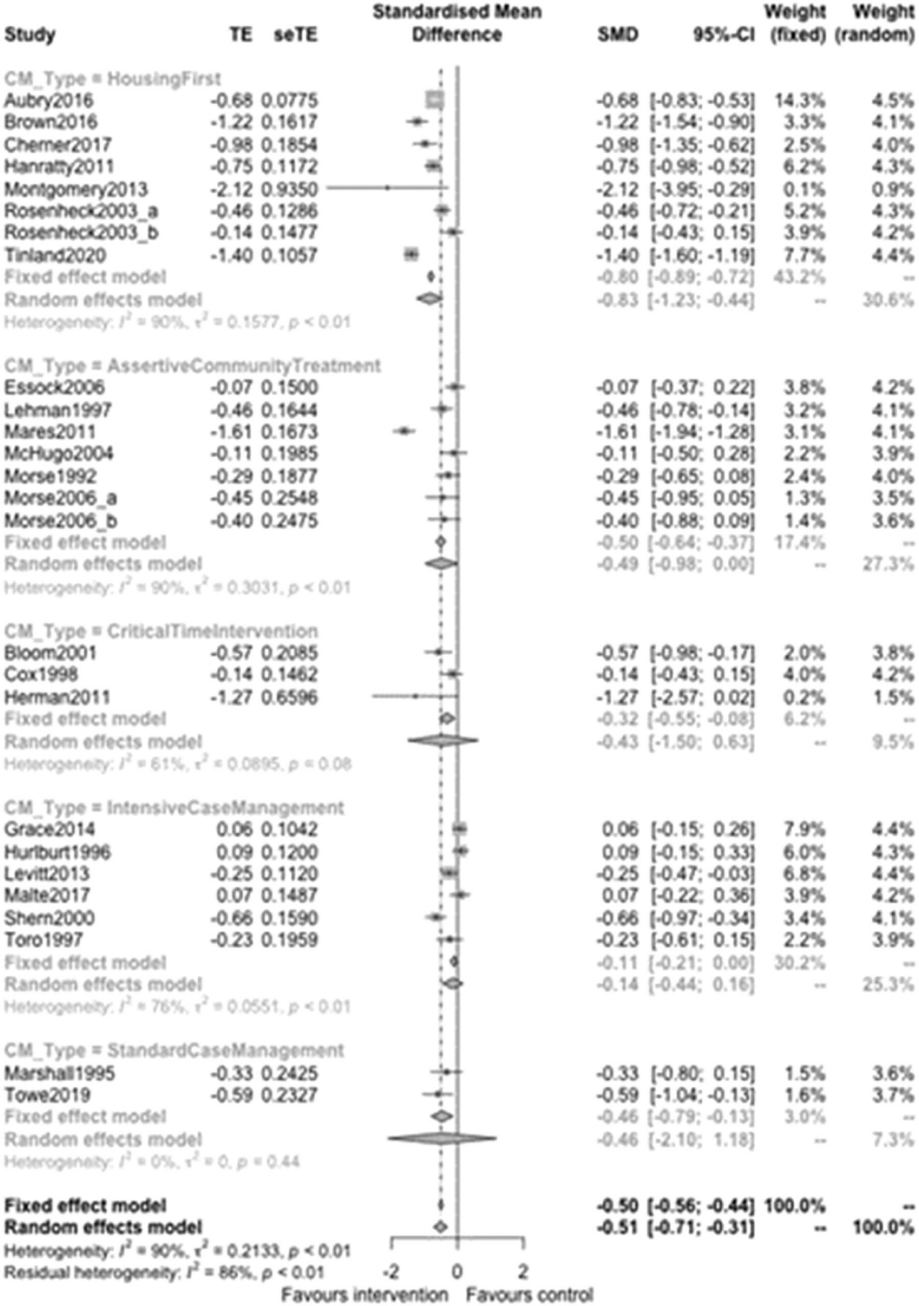
Generic inverse variance analysis of homelessness outcomes 1 year or longer split by case management type.

To recap, the generic inverse variance approach augments the previous analyses by adding in studies that reported between group outcomes only (i.e., they did not report outcomes separated by randomised groups, but instead reported just the difference between them). The generic inverse variance approach added four studies to the forest plot, but did not alter the conclusion that case management of any type improves homelessness outcomes for any case management (Figure 19). Generic inverse variance analyses split by case management type did not alter the previous conclusions either (Figure 20).

#### 5.3.2 Mental health outcomes

An important, and frequently reported, secondary outcome was mental health and this was explored in detail within the analyses.

**Figure 21:**
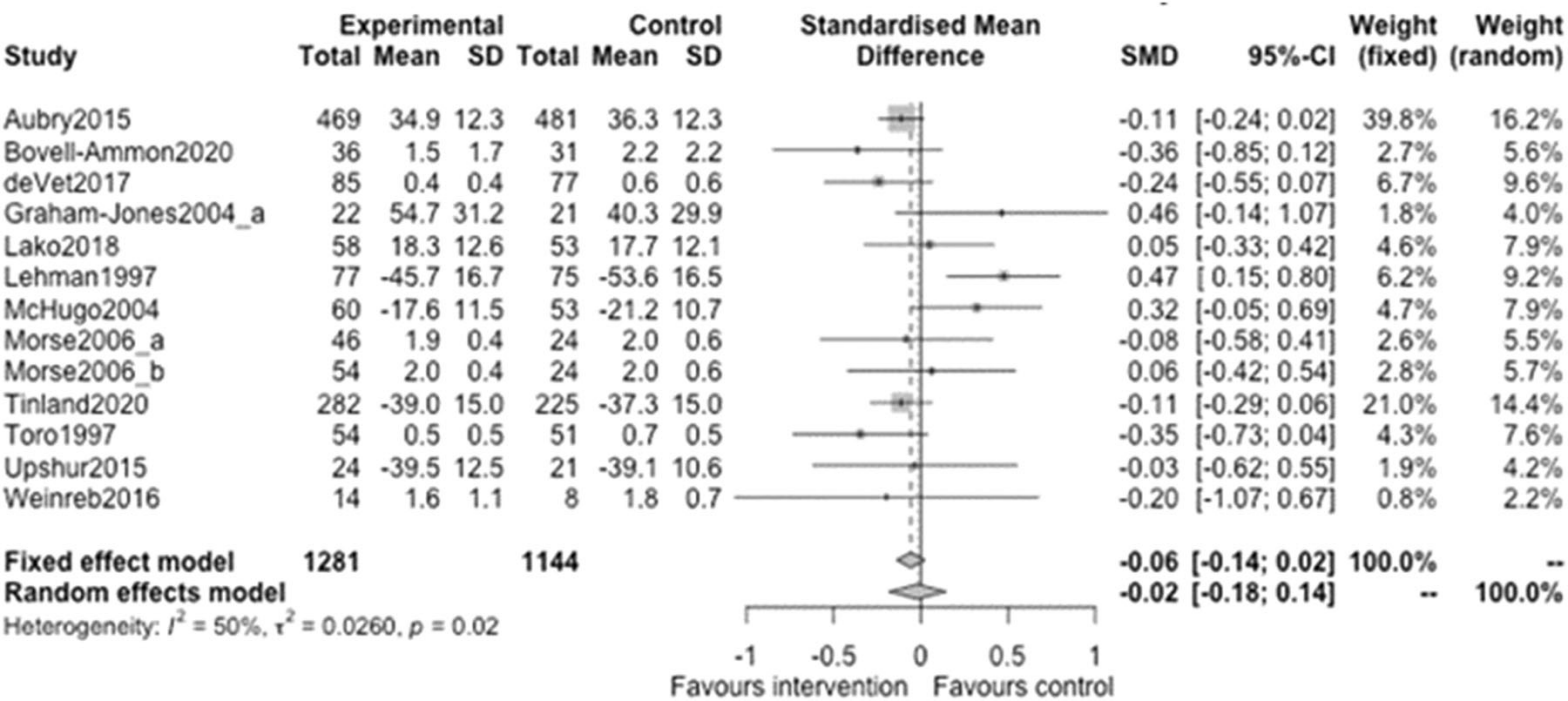
Mental health outcomes less than 1 year.

Figure 21 shows that the overall estimate of effectiveness was −0.02 (95% CI: [−0.18, 0.14]), but was not statistically significant (*p* value = 0.792). Heterogeneity was moderate (*I* ^2^ = 0.5). This indicates that case management overall was not effective at altering mental health outcomes in the short (less than 12 months) term.

**Figure 22:**
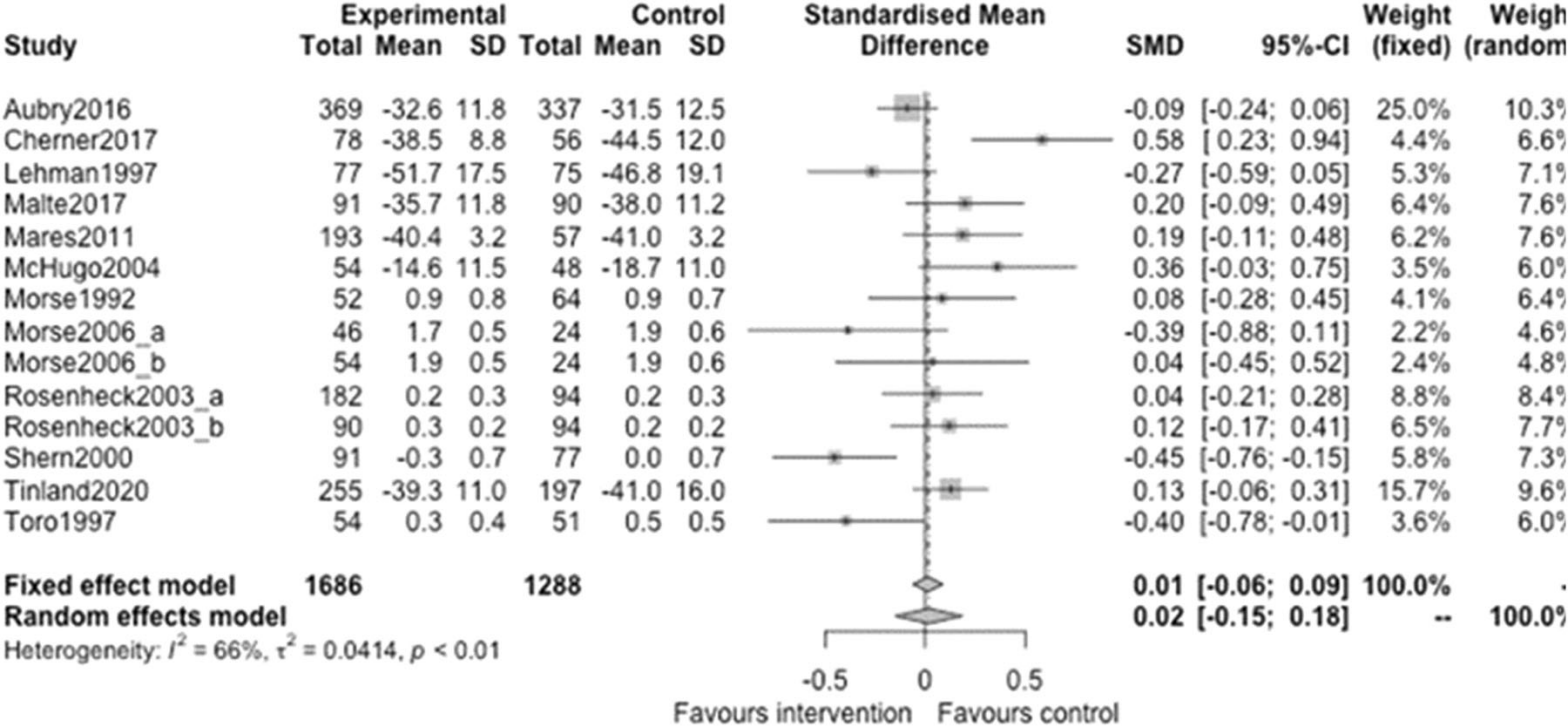
Mental health outcomes 1 year or longer.

Figure 22 shows that the overall estimate of effectiveness was 0.02 (95% CI: [−0.15, 0.18]), but was not statistically significant (*p* value = 0.817). Heterogeneity was moderate (*I* ^2^ = 0.66). Again, here this final diamond is almost perfectly on 0 (the point of no intervention effect). This indicates that case management overall was not effective at altering mental health over longer follow‐ups (12 months or longer).

**Figure 23:**
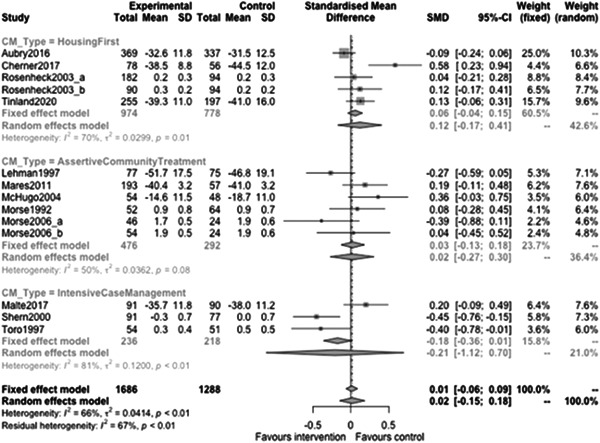
Mental health outcomes 1 year or longer split by case management type.

Figure 23 shows that case management type subgroup effectiveness was 0.12 (95% CI: [−0.17, 0.41]) for Housing First, and 0.02 (95% CI: [−0.27, 0.3]) for Assertive Community Treatment, and −0.21 (95% CI: [−1.12, 0.7]) for Intensive Case Management but none of these were statistically significantly different from 0.

**Figure 24:**
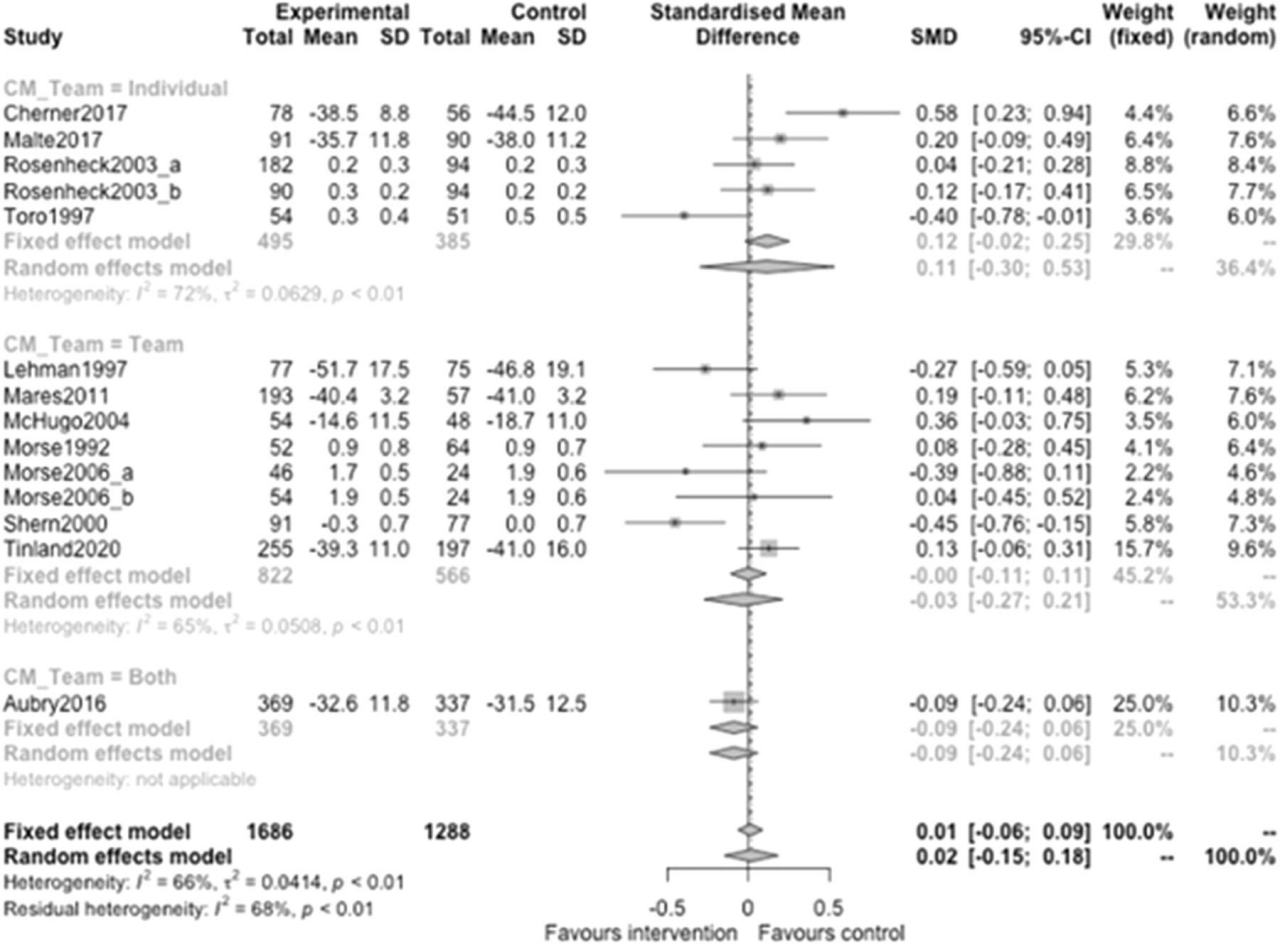
Mental health outcomes 1 year or longer split by team‐individual case manager.

Figure 24 shows that team/individual subgroup effectiveness was −0.03 (95% CI: [−0.27, 0.21]) for individual case managers, and −0.09 (95% CI: [−0.24, 0.06]) for when there was a team of case managers but none of these were statistically significantly different from 0.

**Figure 25:**
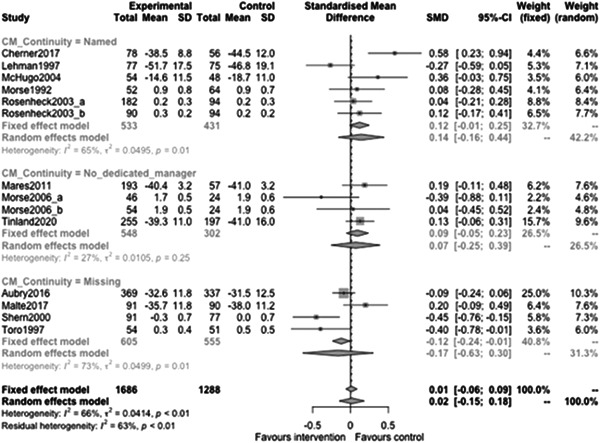
Mental health outcomes 1 year or longer split by continuity of care.

Figure 25 shows that continuity of care subgroup effectiveness was 0.07 (95% CI: [−0.25, 0.39]) for named case managers, and −0.17 (95% CI: [−0.63, 0.3]) for when there was no dedicated case manager but none of these were statistically significantly different from 0.

**Figure 26:**
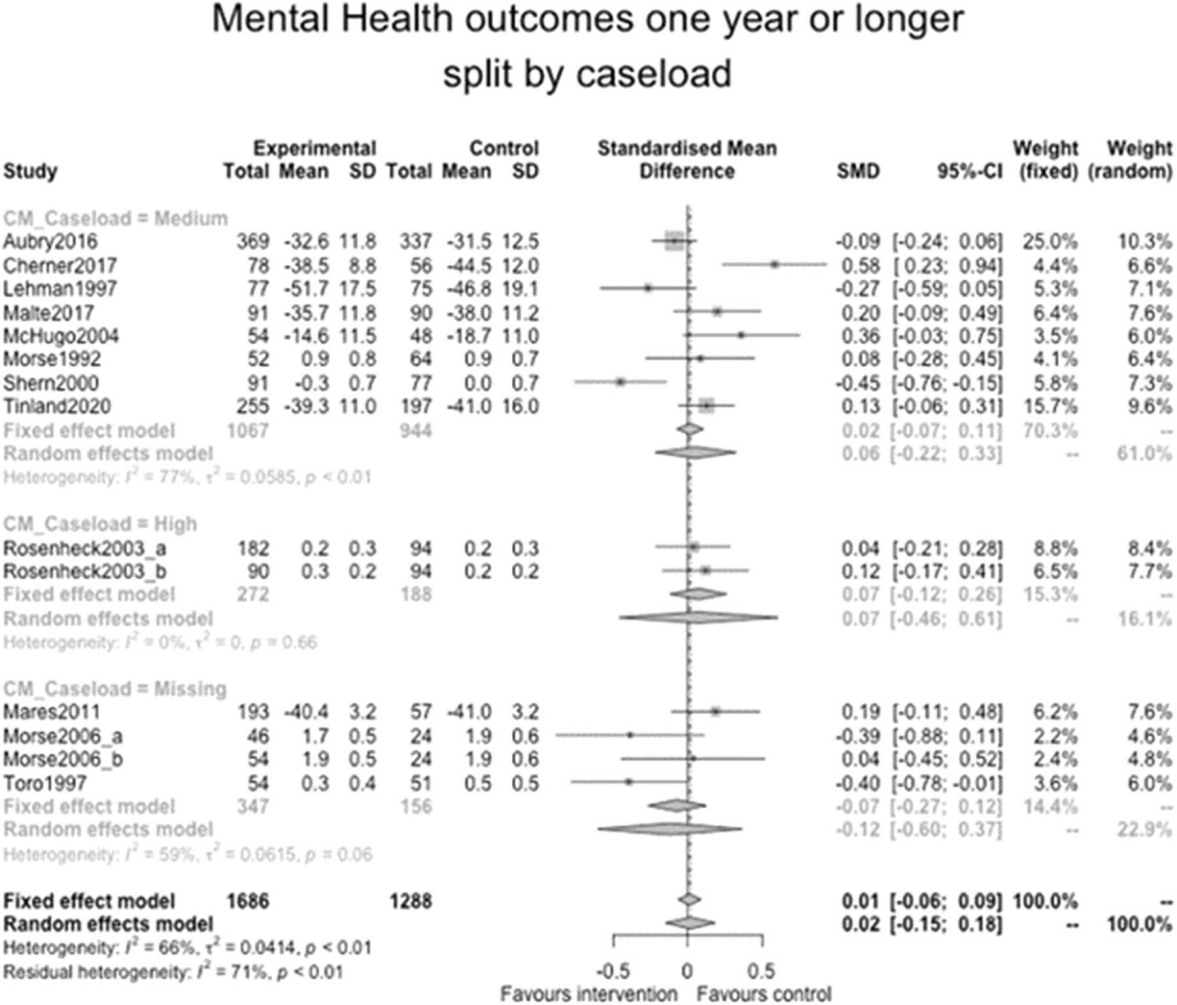
Mental health outcomes 1 year or longer split by caseload.

Figure 26 shows that continuity of caseload subgroup effectiveness was 0.06 (95% CI: [−0.22, 0.33]) for medium case loads (8–20 PEH per case manager), and 0.07 (95% CI: [−0.46, 0.61]) high case loads (21 or more PEH per case manager) but none of these were statistically significantly different from 0.

**Figure 27:**
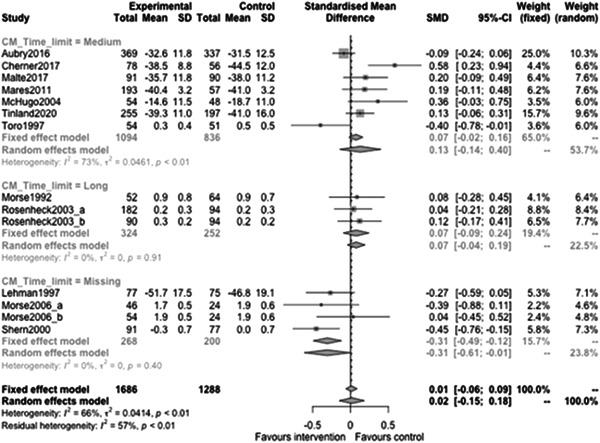
Mental health outcomes 1 year or longer split by time limit of provision.

Figure 27 shows that time limit of provision subgroup effectiveness was 0.13 (95% CI: [−0.14, 0.4]) for medium time limits (more than 6 months to less than 3 years), and 0.07 (95% CI: [−0.04, 0.19]) long time limits (3 years or longer) but none of these were statistically significantly different from 0 (the missing group did show statistically significant benefits, but this is difficult to interpret).

**Figure 28:**
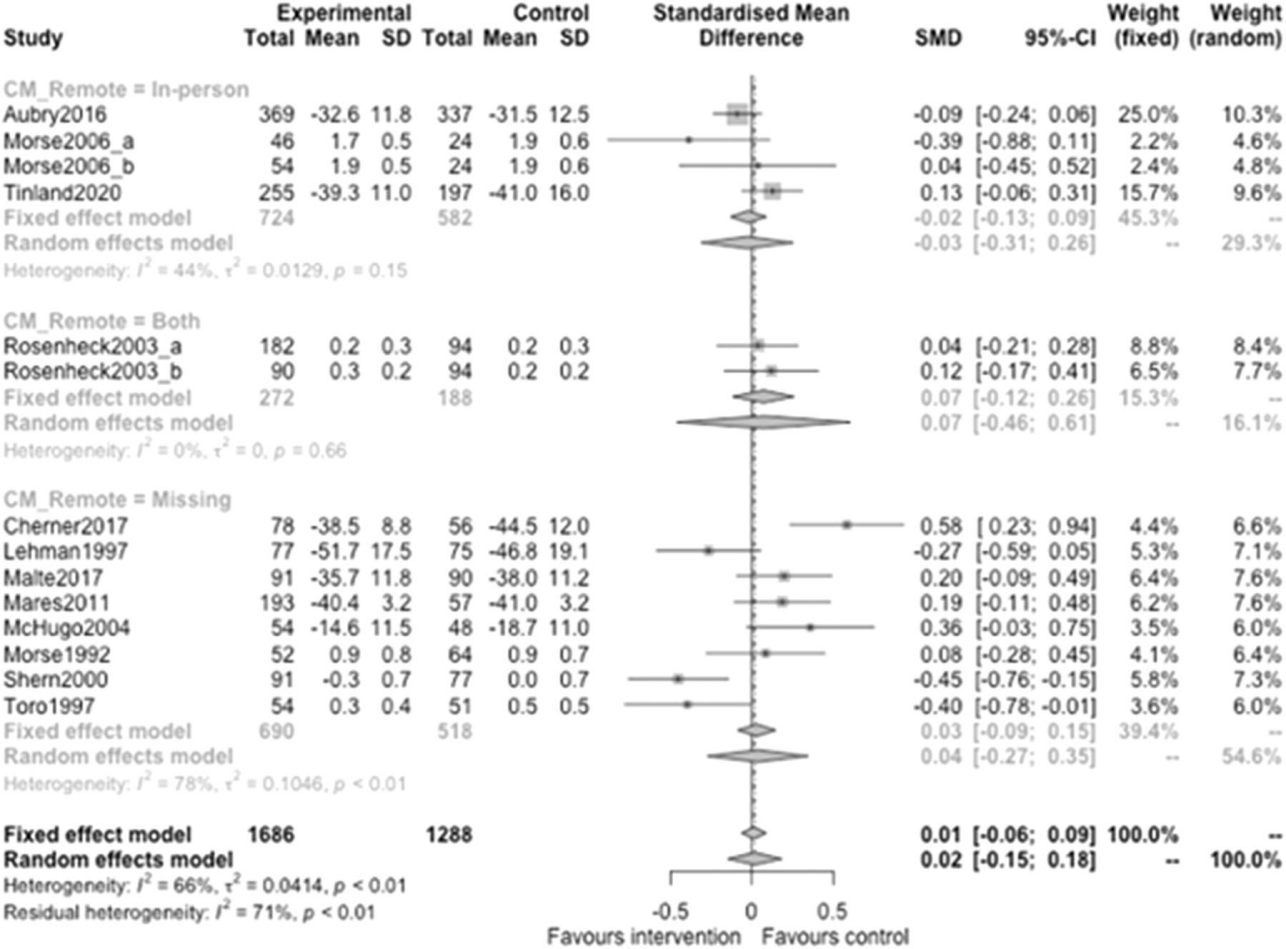
Mental health outcomes 1 year or longer split by remote or in‐person.

Figure 28 shows that in‐person/remote provision subgroup effectiveness was −0.03 (95% CI: [−0.31, 0.26]) for in‐person provision, and 0.07 (95% CI: [−0.46, 0.61]) when both in‐person and remote provision were used.

**Figure 29:**
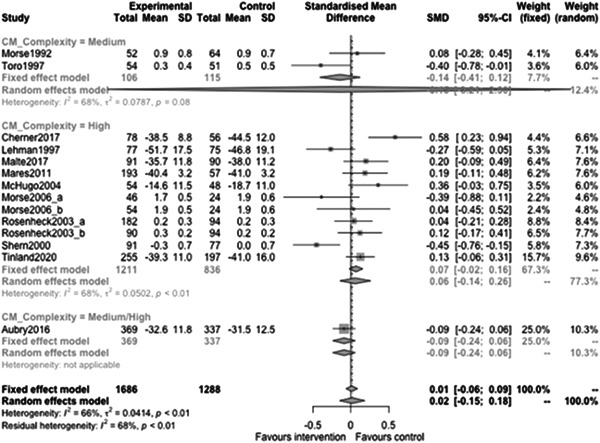
Mental health outcomes 1 year or longer split by complexity of need.

Figure 29 shows that the complexity of need subgroup effectiveness was −0.15 (95% CI: [−3.21, 2.9]) for medium complexity, and 0.06 (95% CI: [−0.14, 0.26]) high complexity, but none of these were statistically significantly different from 0.

**Figure 30:**
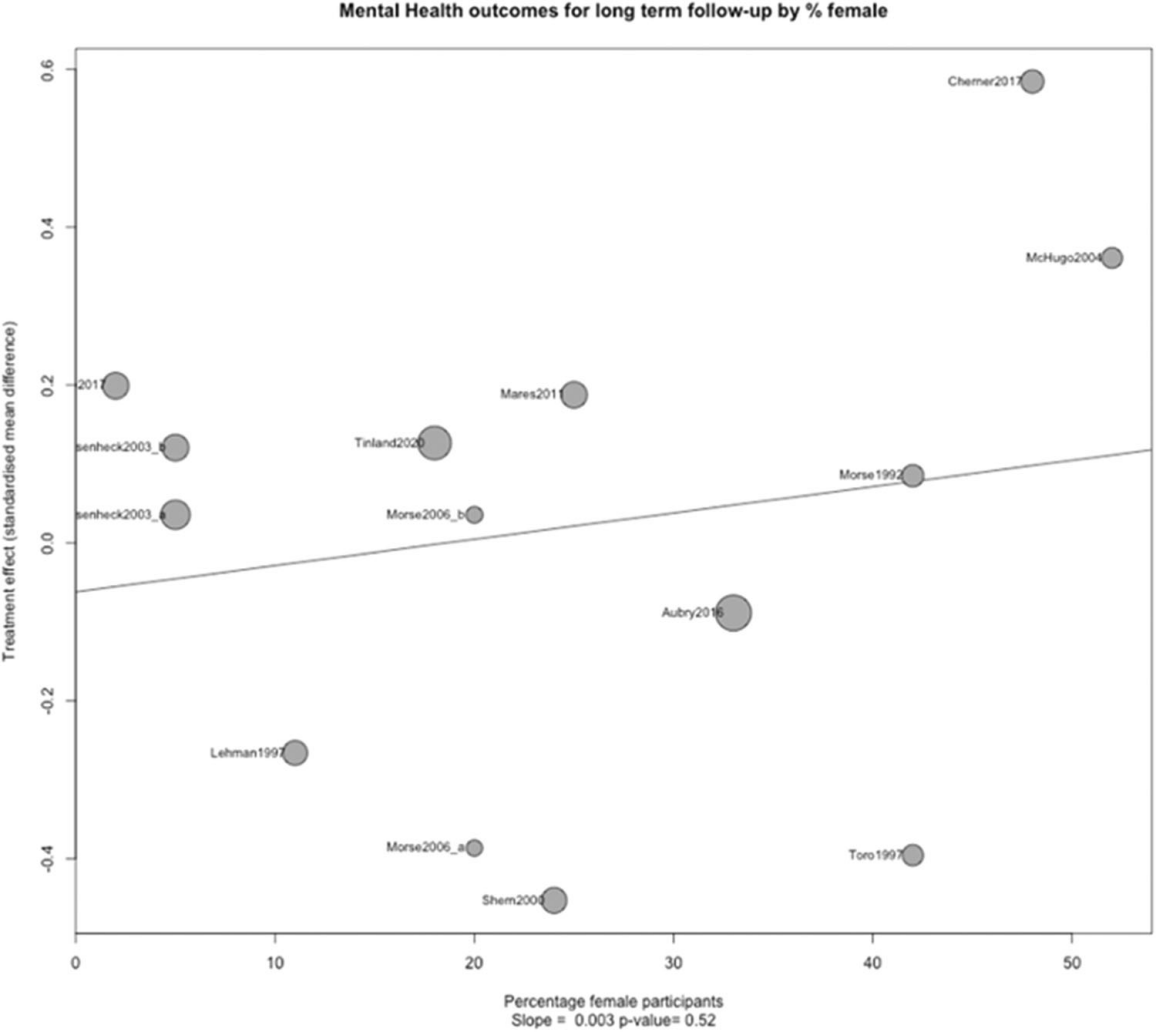
Meta‐regression of mental health outcomes 1 year or longer by % female.

Figure 30 shows that despite the upward slope of this fitted line there is no statistically significant relationship between mental health outcomes and the proportion of female participants (slope: 0.003, *p* value: 0.52).

#### 5.3.3 Substance use outcomes

**Figure 31:**
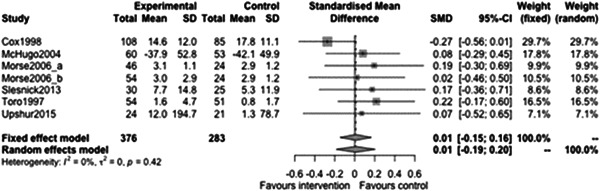
Substance use outcomes of less than 1 year.

Figure 31 shows that there is no statistically significant effect of case management on substance use outcomes at follow‐ups less than 12 months (estimate: 0.01, 95% CI: [−0.19, 0.2], *p* value: 0.91). Indeed the point estimate here is tiny and the CI rules out large effects in either direction.

**Figure 32:**
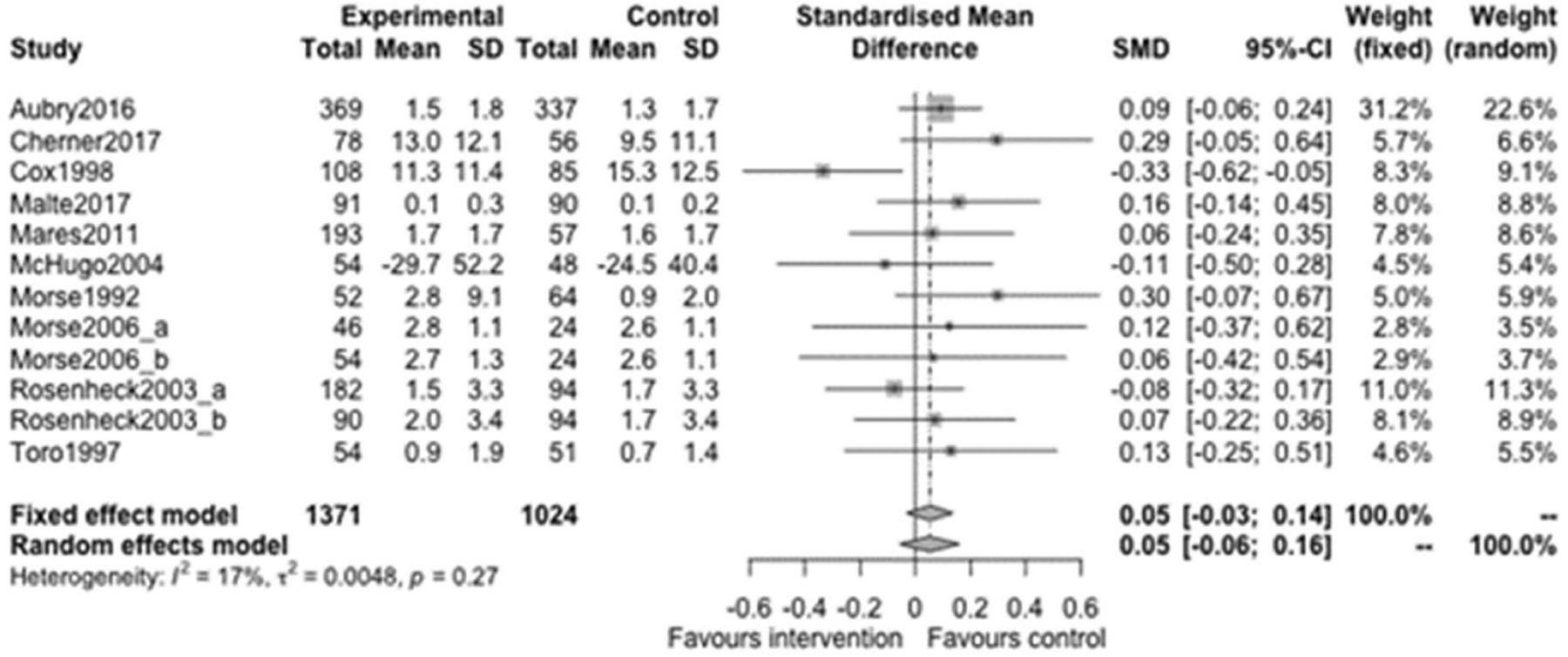
Substance use outcomes 1 year or longer.

Figure 32 shows that there is no statistically significant effect of case management on substance use outcomes at follow‐ups 12 months or longer (estimate: 0.05, 95% CI: [−0.06, 0.16], *p* value: 0.31). Again, the range of plausible values for the effect does not include large protective (or indeed harmful) effects of case management on substance use outcomes.

#### 5.3.4 Physical health outcomes

**Figure 33:**
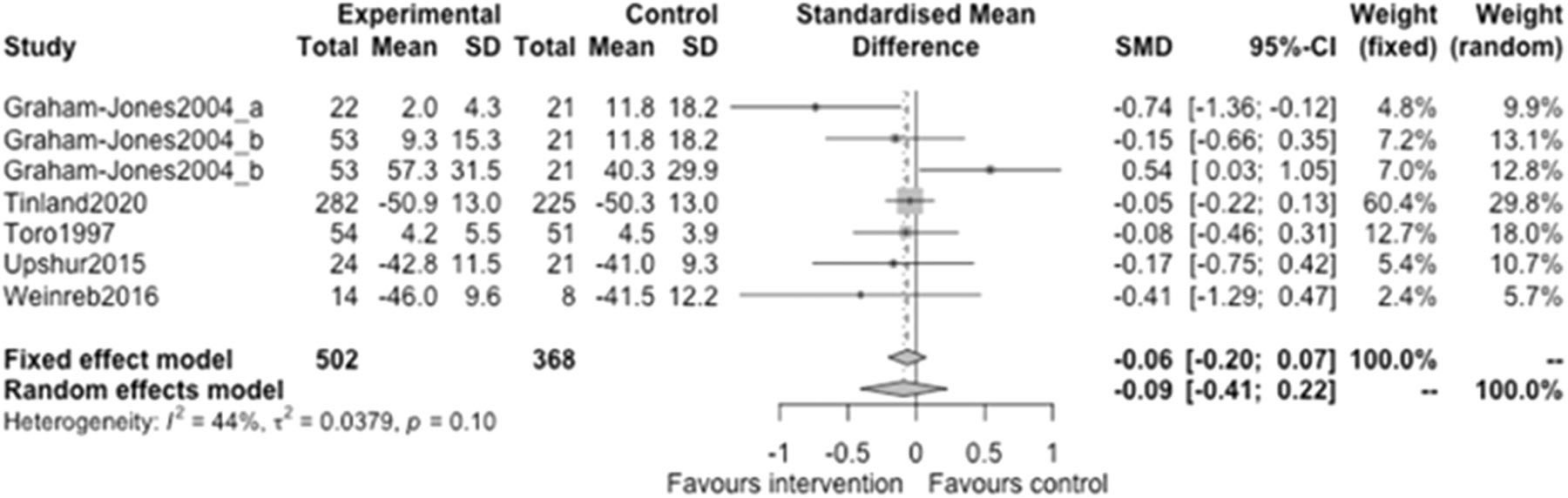
Physical health outcomes less than 1 year.

Figure 33 shows that there is no statistically significant effect of case management on physical health outcomes at follow‐ups less than 12 months (estimate: −0.09, 95% CI: [−0.41,0.22], *p* value: 0.5).

**Figure 34:**
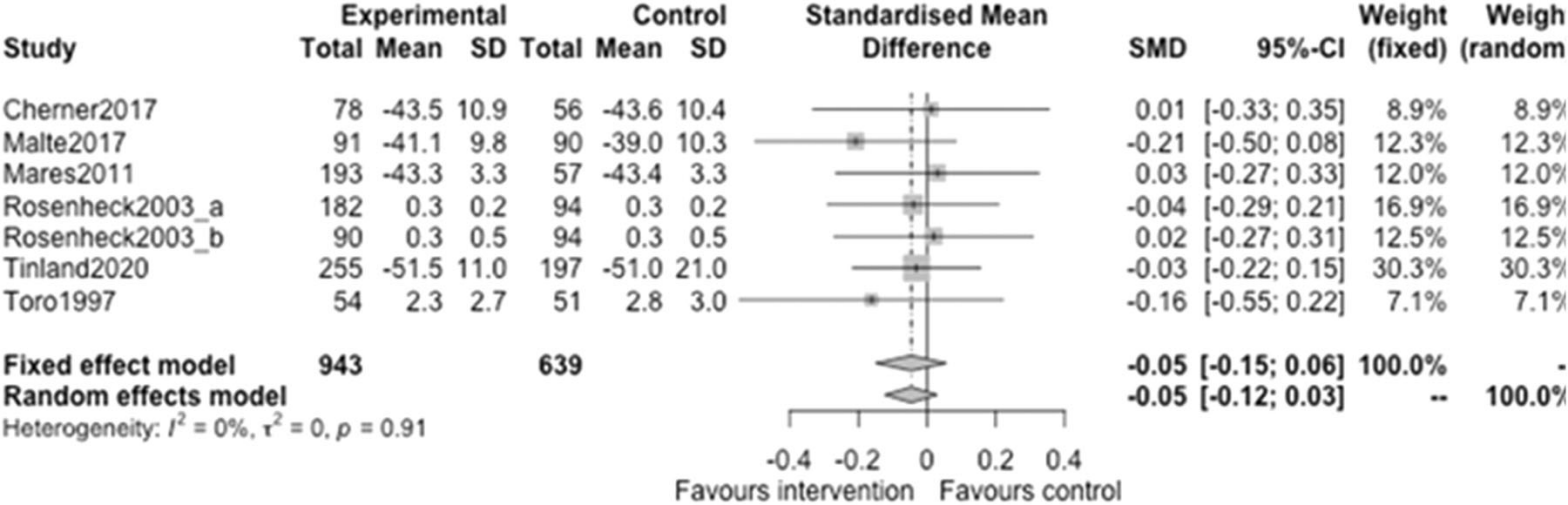
Physical health outcomes 1 year or longer.

Figure 34 shows that there is no statistically significant effect of case management on physical health outcomes at follow‐ups 12 months or longer (estimate: −0.05, 95% CI: [−0.12, 0.03], *p* value: 0.19).

#### 5.3.5 Capabilities and wellbeing outcomes

**Figure 35:**
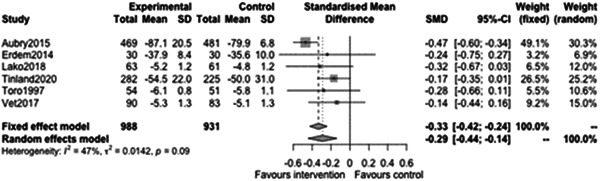
Capabilities and wellbeing outcomes less than 1 year.

Figure 35 shows that there is a statistically significant effect of case management on Capabilities and Wellbeing outcomes at follow‐ups less than 12 months (effect: −0.29, 95% CI: [−0.44, −0.14], *p* value: <0.01). This equates to an improvement of roughly one‐third of a standard deviation in outcome for the case management intervention.

**Figure 36:**
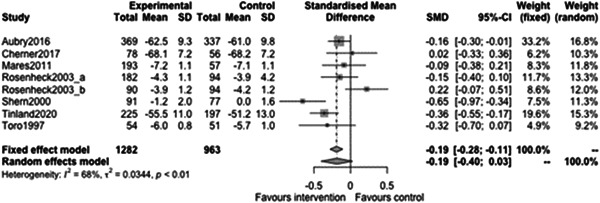
Capabilities and wellbeing outcomes 1 year or longer.

Figure 36 shows that there is no statistically significant effect of case management on Capabilities and Wellbeing outcomes at follow‐ups 12 months or longer (estimate: −0.19, 95% CI: [−0.4, 0.03], *p* value: 0.08). However, most of the individual studies favour intervention and the CI excludes substantial harm.

#### 5.3.6 Employment outcomes

**Figure 37:**
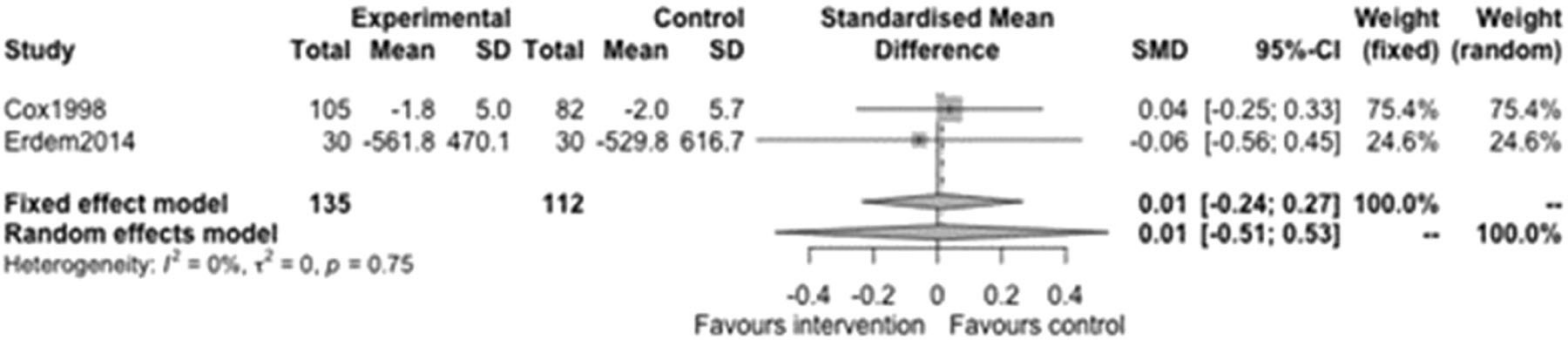
Employment outcomes less than 1 year.

Figure 37 shows that there is not a statistically significant effect of case management on employment outcomes at follow‐ups less than 12 months (estimate: 0.01, 95% CI: [−0.51, 0.53], *p* value: 0.79). The point estimate is very small and the CI includes moderate effects in either direction indicating there is little evidence to support any effect.

**Figure 38:**
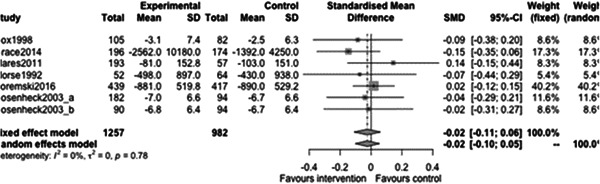
Employment outcomes 1 year or longer.

Figure 38 shows that there is no statistically significant effect of case management on employment outcomes at follow‐ups 12 months or longer (estimate: −0.02, 95% CI: [−0.1, 0.05], *p* value: 0.48). The CI here rules out even small effects in either direction.

The homelessness finding in favour of a case management intervention is notable. A typical variance in any measured outcome will be a single standard deviation (SD) so a statistically significant results of half a SD in favour of an intervention is considerable. The measured outcomes for housing varied and details for each included study can be viewed at https://github.com/MarkKelson/CHI_analysis. Taking the At Home/Chez Soi study as an example, where the measure was % housed (Aubry 2016), 20% more PEH receiving the intervention were housed at 12 months than those in the usual care control group.

#### 5.3.7 Case management comparisons with usual care not included in the meta‐analyses

Some of the interventions that compared case management with a usual care group could not be included in the meta‐analyses because appropriate data were not available within the study reports. Findings from these studies are summarised briefly below. The studies and their outcomes were heterogeneous in nature but, overall, findings support the results from meta‐analyses that case management provides superior housing (but not mental health) outcomes when compared to usual care.

1. Three studies (Calsyn, 2005; Ferreiro, 2020; Lapham, 1993) compared **ACT** to usual care. One (Lapham, 1993) looked at housing outcomes and found no difference in stable housing days (past 30 days) between groups. Two studies (Calsyn, 2005; Ferreiro, 2020) explored mental health outcomes and found no differences across groups.
2. One study (Gulcur, 2003) compared **ACT within a Housing First model** with usual care (continuum of care) and concluded, from a modelling exercise, that Housing first (ACT) was associated with smaller proportions of time homeless and in psychiatric hospitals, while a companion paper (reported in Greenwood, 2005) did not measure any differences in psychiatric symptoms.
3. Four studies (Baumgartner, 2012; Samuels, 2015; Shaw, 2017; Shinn, 2015) compared **CTI** to usual care. Only one of these included a homelessness outcomes (Baumgartner, 2012) and noted a significant reduction in homelessness for the CTI versus usual care group at 14–18 month months (OR: 0.22, 95% CI: 0.01, −0.96). This is compatible with the findings for the studies that could be included in a meta‐analysis (Figure 19). Baumgartner 2012 also noted a reduction in psychiatric hospitalisations for the CTI versus usual care group (OR = 0.11 [95% CI: 0.01, −0.96]). The other three studies (Samuels, 2015; Shaw, 2017; Shinn, 2015) explored mental health outcomes only. Samuels (2015) noted that mothers experiencing homelessness reported significant declines in mental health, regardless of intervention condition. Shaw (2017) explored engagement with the community health team of discharged prisoners and found that the intervention significantly improved contact at 6 weeks but this diminished with time and was non‐significant at 12 months. Shinn 2015 explored mental health outcomes for children, noting no effect for most mental health measures.
4. One study (Braucht, 1996) compared **ICM** to usual care. Braucht 1996 found no significant difference in housing status or mental health outcomes noting that these improved in both groups. Slesnick 2015 compared **ICM to other types of therapy** (community reinforcement or motivational interviewing) and found no difference in substance use outcomes between groups although they reduced across time in all groups.
5. Thre studies (Burt, 2012; Conrad, 1998; Nyamathi, 2001) compared **SCM** to usual care. Burt 2012 found that intervention clients spent more days housed than the usual care group at three months (Intervention mean ± SD = 79 ± 97 days; comparison group 3 ± 35 days). For Conrad 1998, an initial reduction in homelessness in the intervention group compared to control diminished with time and reversed at 24 months. Significant improvements in some addiction measures were noted. Nyamathi (2001) explored psychological factors only, noting no difference across groups.

**Case management comparisons without another type of case management but no usual care control group**:

Some studies did not compare a case management intervention with usual care and these are summarised briefly below, with additional information in Supporting Information: Appendix 3.

The evidence to date can be summarised as below. Overall, the findings do not add strength to the suggestion from the meta‐analyses that there may be a small advantage from ACT over other types of case‐management. There may be a trend for the more intensive approaches of ACT and ICM to be superior to standard case management. However, the studies were few and highly heterogeneous in nature so no clear conclusions can be drawn.

1. One study (Calsyn, 1998 Trial 2) found improved housing and mental health outcomes for **ACT as compared to Brokered Case Management (BCM)**. The number of days housed per month in the ACT versus BCM groups increased by 14.9d for BCM and 20.9d for ACT groups (40% difference, *p* = 0.0015). Psychiatric symptoms improved for the ACT group and worsened for BCM but this was not significant (reported in Kenny, 2004).
2. Three studies compared **ACT to SCM** (Essock, 2006; Frisman, 2009; Morse, 2006). Morse (2006) found that ACT resulted in improvements in days living in stable housing for ACT compared with SCM (*p* = 0.03). Two studies (Essock, 2006; Frisman, 2009) did not measure a significant difference in housing outcomes and none of the three studies measured a significant difference in mental health outcomes.
3. One study compared **ACT to ICM** (McHugo, 2004) finding no significant difference in housing outcomes but a significant improvement in reported psychiatric symptoms for the ICM versus ACT group (*p*: 0.01).
4. One study (Clark, 2016) compared **ACT to CTI** and identified no significant difference in subjects likely to be housed at 6 months but noted a significant decrease in psychiatric symptoms for the CTI compared to the ACT group (*p* < 0.01).
5. One study (Kidd, 2020) compared **CTI to SCM** finding no significant difference in housing or mental health outcomes.
6. Three studies (Buchanan, 2009; Cheng, 2007; Ellison, 2020) compared **ICM to SCM**. Two (Cheng, 2007; Ellison, 2020) looked at housing outcomes. Cheng (2007) favoured ICM over SCM for housing (days housed, *p* < 0.001) but no significant differences in mental health outcomes (reported in Rosenheck, 2003). Ellison (2020) found no difference between groups in housing stability as measured by the number of days spent in HUD‐VASH housing in the past 30 days. Buchanan (2009) explored HIV‐immunity in HIV‐positive patients, noting that 55% of the intervention and 34% of the control group were alive with intact immunity at 1 year (*p* = 0.04).

**Case management variations within one type of case management:**

1. Two studies (Hall, 2018; Padgett, 2011) explored issues around case management with abstinence requirements and tend to support the Housing First approach whereby abstinence is not a requirement for housing assistance. Hall (2018) looked at **SCM with and without substance abuse treatment** and concluded that residential stability can be achieved without substance abuse treatment before housing admission. Treated participants had a greater likelihood of housing discharge than active users (adjusted hazard ratio [AHR] 1.43, 95% CI: 1.17–1.73). Padgett (2011) compared the **Housing First to Treatment First** approaches and found that the Housing First group had significantly lower rates of substance abuse and substance abuse treatment, and were less likely to leave the programme. 11% of Housing First participants left the programme compared to 54% treatment first participants (*p* = 0.000).
2. In contrast, Koffarnus 2011 explored the very specific issue as to whether **case‐managed, paid employment training (the Therapeutic Workplace) contingent on abstinence**, could promote alcohol abstinence when compared with a paid or unpaid training, not contingent on abstinence. Based on multiple measures, authors concluded that pay improved workplace attendance and, additionally, that access contingent on breathalyzer results improves abstinence without reducing workplace attendance.

There were two other, very different studies.

1. Hurlburt (1996) found that clients with **access to housing certificates within ICM** were 4.87 times more likely to achieve independent housing than those with the same case management support but no housing certificates (*p* < 0.05).
2. Bender 2018 examined **SCM versus SCM with a risk detection intervention** (a very short term intervention workshop (3 days) ‘Safety Awareness for Empowerment (SAFE)’). This was a small urban US study and the small size of the study (*N* = 97) and high attrition (nearly 24% at 6 months) limit the reliability of the findings that suggest that the intervention group improved their risk detection abilities significantly more than the control group (*p* = 0.018).

**Case management to influence treatment adherence or vaccination status in PEH:**

Nyamathi and colleagues carried out three studies exploring the effect of case management interventions on treatment and vaccination adherence. In an exploration of nurse case‐managed Isoniazid treatment for tuberculosis with an incentive compared to usual care (a single educational session), intervention PEH were almost twice as likely to complete treatment as those in the control group. Completion rates exceeded corresponding rates of control participants by 20% or more for all participant subgroups other than daily drug users (Nyamathi, 2008).

In another study looking at nurse case management plus incentives and tracking (NCMIT) to increase hepatitis vaccination, compared to education and incentives plus tracking (SIT) or education and incentives only (SI). 68% of the NCMIT group had all three doses of HAV/HBV vaccine compared to 61% in the SIT and 54% in the SI group (*p* < 0.05) (Nyamathi, 2009; Nyamathi, 2009a). A further study exploring hepatitis vaccine completion rates amongst PEH receiving intensive peer coaching and nurse case management compared to intensive peer coaching with minimum nurse involvement and usual care found no significant difference in vaccine completion rates across the groups (Nyamathi, 2015; Nyamathi, 2016).

**Cost effectiveness studies**:

Twelve very different studies provided cost or cost effectiveness information comparing case management approaches compared to usual care. Six studies looked at Housing First (Gilmer, 2009; Latimer, 2019; Latimer, 2020; Stergiopoulos, 2015; Tinland, 2020; Tonks, 2009) with a mixture of ACT and ICM approaches; three at ACT (Lehman, 1999; Mares, 2011; Morse, 2006); two at ICM (Nyamathi, 2016; Rosenheck, 2003) and one at CTI (Shaw, 2017). Findings were contrasting and there is too little evidence, as yet, to draw clear conclusions although there are some trends. Some case management costs may be off‐set by reductions in use of other services but this approach will probably incur additional costs. Cost effectiveness may be measured on the basis of Society's ‘willingness to pay’ for each additional day of stable housing.

***Housing First**:* Overall, there is a consistent finding that case management costs increase with Housing First but there are some reductions in costs of health service use (Gilmer, 2009; Tinland, 2020; Tonks, 2009). Gilmer (2009) found that case management costs increased by $6,403 (*p* < 0.001) from pre‐ to postintervention, but inpatient plus emergency services costs declined by $6,103 (*p* = 0.034), and costs for mental health services provided by the criminal justice system declined by $570 (*p* = 0.020) compared with the control group. Overall there was no significant difference in total costs for Housing First compared to usual care clients. Tinland (2020) found that, compared to usual care, Housing First participants spent 48% less on health care (mean € 29 454 v. € 47 570; RR 0.62, 95% CI, 0.48–0.78) and 93% less on standard residential services (mean €687 v.€8963; RR 0.07, 95% CI, 0.05–0.11). Tonks 2009 found that medical bills fell considerably in the Housing First group with a total cost rate reduction of 53% for housed participants versus wait list control (rate ratio: 0.47; 95% CI: 0.25–0.88).

Latimer and colleagues carried out two studies comparing Housing First with ICM (Latimer, 2019) and Housing First with ACT (Latimer, 2020) with usual care. For ICM, at a ‘willingness of pay’ threshold of 67 Canadian Dollars per day of stable housing, there was an 80% chance that HF was cost‐effective compared with usual care (Latimer, 2019). Housing First with ACT had more than an 80% chance of being cost‐effective with a threshold of 60 Dollars. Latimer (2020) concluded that Housing First with ACT is about as cost effective as Housing First with ICM for PEH with moderate needs. A direct contrast with Stergiopoulos (2015) who concluded that the cost of supportive housing with ICM services was CaD $14 177 per participant annually, approximately30% less than the cost of supportive housing with ACT (CaD $22 257).

***ACT**:* Lehman (1999) observed that, compared with usual care, ACT costs were significantly lower for mental health in‐patient days and mental health emergency room care, and significantly higher for mental health outpatient visits and treatment for substance misuse. ACT and usual services incurred $242 and $415 respectively in direct treatment costs per day of stable housing, an efficiency ratio of 0.58 in favour of ACT. In contrast, Mares (2011) found that total quarterly healthcare costs were significantly higher for ACT clients than usual care subjects ($4544 vs. $3326) due to increased use of outpatient mental health and substance abuse services.

Morse (2006) did not include a usual care condition but concluded that integrated ACT (IACT, where the same team provides mental health and substance abuse services) and standard case management (usual CM) costs were less than those for standard ACT (ACTO). Total costs at 19–24 months per intervention (SD) were: IACT $11,744 (9220); ACTO ($19,139 (20,649); Usual CM $8,805 (13,039).

***ICM**:* Looking at the very specific issues of usual care versus ICM for newly release parolees, Nyamathi (2016) concluded that usual care was as effective for housing outcomes as ICM. Rosenheck (2003) calculated that the ICM approach used in HUD‐VASH costs $45 more than usual care for each additional day housed, another piece of evidence in relation to ‘willingness to pay’.

***CTI**:* Shaw (2017) found that, after prison release, the overall average contact (excluding inpatient services) was higher for the intervention group. Cost‐effectiveness analysis indicated that an extra cost of £15,426 would be incurred for every extra person engaged at 1 year after release.

See Supporting Information: Appendix 3 for additional detail on study designs.

#### 5.3.8 Impact of bias and heterogeneity on findings

Although there was little sign of publication bias (Figure 6) only 17 of the 64 intervention studies (27%) were assessed as having a high quality methodology (low risk of bias) and 36 (56%) were assessed as low quality (high risk of bias).

For the 37 studies included in meta‐analyses (see Figure 4) the study quality was slightly higher: 11 studies were assessed as high quality (30%) (Aubry, 2016; Collins, 2020; de Vet, 2017; Grace, 2014; Herman, 2011; Hurlburt, 1996; Kidd, 2020; Sadowski, 2009; Slesnick, 2013; Tinland, 2020; Towe, 2019) 9 as medium (24%) (Bloom, 2001; Erdem, 2014; Hanratty, 2011; Lehman, 1997; Malte, 2017; Marshall, 1995; Nyamathi, 2016; Upshur, 2015; Weinreb, 2016) and 17 as low quality (46%). Of the 17 studies rated as low quality, for 13 of these (Bovell‐Ammon, 2020; Bradford, 2005; Brown, 2016; Cox, 1993; Essock, 2006; Graham‐Jones, 2004; Lako, 2018; McHugo, 2004; Montgomery, 2013; Morse, 2006; Rosenheck, 2003; Shern, 2000; Toro, 1997) the low quality assessment was due to a single factor of not providing separate attrition rates for both intervention and control arms (White, 2019; see Assessment of risk of bias in included studies). Nevertheless, there is a risk of bias across the body of included studies. In addition there is high heterogeneity amongst studies for a number of the meta‐analyses (hence the use of random effects models) and the implications of this heterogeneity are discussed with the analyses.

Overall, despite the study quality issues and heterogeneity of studies within the meta‐analyses, the consistency of findings suggest that there can be some confidence in the positive findings for housing outcomes. Similarly, the consistency of findings, and medium heterogeneity, suggest that there can be some confidence in the findings that interventions are unlikely to influence mental health outcomes.

#### 5.3.9 Implementation synthesis

The purpose of synthesising data on views and experiences of case management programmes was to address the question:

What is known about the implementation and process factors that may impact on intervention delivery in terms of case management approach, intervention components and recipient characteristics.

Themes from a purposive sample of 41 studies were extracted as described in the methodology (see Treatment of Qualitative Research). Findings are summarised in Table 1, listing the number of studies that identified each of the themes. The number of studies is listed for information and should not be taken as inferring a hierarchical order since there are other important factors that would influence how much confidence a reader might place in each finding (e.g. the quality of each contributing study and the relevance of study populations and settings to one's own context). Further detail is provided in the text below the Table. Themes relating to local interagency and national policy and guidance were surfaced by the research, along with those looking at the specific components of case management, which is the main focus of this review. Since the broader themes are relevant to the context of face to face practice, and to practitioners and policy makers, the findings from the analysis have been presented in full.

**Table 1:** Summary of factors that may impact case management programme delivery.

Theme	Specific recommendations related to the theme (where consensus from 2 + studies)
**Face to face practice: Case management components**	
**Conditionality** Barriers due to conditions attached to services 9 studies: Choy‐Brown et al., 2021, Clifasefi et al., 2016, Cole, 2017, Kirst et al., 2014, Montgomery and Cusack, 2017, Montgomery et al., 2019, Roberts, 2012, Stanhope et al., 2015, Toombs et al., 2021	Minimise conditionality: Clifasefi, 2016; Cole, 2017; Kirst, 2014; Montgomery, 2017; Montgomery, 2019; Roberts, 2012; Stanhope, 2015; Toombs, 2021
**Team versus individual case management** 7 studies: Fleury, 2014, Flowers, 2014; Miller, 2001; Montgomery, 2017; Montgomery 2019; Ponce, 2018; Vitopoulos, 2018	Include CM/peer mentors with lived experience: Montgomery, 2017; Vitopoulos, 2018
**Continuity of care** Desirability of case manager continuity 6 studies: Blosnich, 2020; Clark, 2016; de Vet, 2017; Ploeg, 2008, Shepherd, 2019, Vitopoulos, 2018	Maintain continuity of care: Blosnich, 2020; Clark, 2016; de Vet, 2017; Ploeg, 2008; Shepherd, 2019; Vitopoulos, 2018
**Professional versus non‐professional case manager** 5 studies: Collins, 2019. Clifasefi, 2016; Fitzpatrick, 2011; Miller, 2001; Stergiopoulos, 2012	No specific consensus
**Case load** Time availability/case load for case management 5 studies: Austin et al., 2014; Chinman et al., 2017; de Vet et al, 2017; Rog et al., 1997; Vitopoulos et al., 2018	Acknowledge time barriers to care provision: de Vet, 2017; Rog, 1997
**Case manager availability**: Enhancing accessibility of CM services for clients 4 studies: Montgomery and Cusack, 2017; Montgomery et al., 2019; Toombs et al., 2021; Vitopoulos et al., 2018	Provide timely response to clients by CM answering phone calls, calling back same day, location near clients, no waitlists or formal booking systems: Montgomery 2017; Montgomery 2019; Toombs 2021
**Remote/in‐person provision** Appointment location facilitating access 3 studies: Blosnich, 2020; Montgomery, 2019; Toombs, 2021	No specific consensus
**Frequency of contact** Frequency of case manager‐client contact 3 studies: First, 1990; Montgomery, 2017; Newman, 2017	Frequent case manager‐client contact: First, 1990; Montgomery, 2017; Newman, 2017
**Time limit of provision** 2 studies: Clark, 2016; Richardson, 1996	Provide extended support: Clark, 2016; Richardson, 1996
**Face to face practice: Other themes relating to the case manager‐PEH relationship**	
Non‐housing support and training needs of clients 14 studies: Choy‐Brown, 2021; Clifasefi, 2016; Cole, 2017; Collins, 2019; García, 2020; Miller, 2001; Montgomery, 2017; Montgomery, 2019; Newman, 2017; Ponce, 2018; Richardson, 1996; Roberts, 2012; Tsai, 2014; Vitopoulos, 2018	Provide training related to: Education, employment, finance, parenting, independent living skills, substance abuse, clothing, medical care: Choy‐Brown, 2021; Clifasefi, 2016; Cole, 2017; Collins, 2019; García, 2020; Miller, 2001; Montgomery, 2017; Montgomery, 2019; Newman, 2017; Ponce, 2018; Richardson, 1996; Roberts, 2012; Tsai, 2014; Vitopoulos, 2018 Independent living skills including cleaning, cooking, washing, sleeping in a bed, budgeting, and making appointments: Cole, 2017; Collins, 2019; García, 2020; Miller, 2001
Community support and development 11 studies: Adame, 2020; Clifasefi, 2016; Cole, 2017; Fleury, 2014; Francis, 2000; Montgomery, 2019; Ponce, 2018; Roberts, 2012; Stergiopoulos, 2012; Toombs, 2021; Tsai 2014	Community building activities like meals, arts performances, trips, cooking classes, game nights, exercise classes, volunteering, community meetings: Adame, 2020; Clifasefi, 2016; Cole, 2017; Montgomery, 2019; Ponce, 2018; Roberts, 2012; Stergiopoulos, 2012; Toombs, 2021 Nurture positive client CM relationships: Adame, 2020; Clifasefi, 2016; Montgomery, 2017; Ponce, 2018; Roberts, 2012; Stanhope, 2007
Client confidence in CM programmes and CM 11 studies: Adame, 2020; Clifasefi, 2016; Cole, 2017; de Vet, 2017; Kirst, 2014; Montgomery, 2017; Ponce, 2018; Stanhope, 2007; Toombs, 2021; Vitopoulos, 2018; Zerger, 2016	Aim for clients perceiving programme as a support system: Adame, 2020; Ponce, 2018 Offer flexibility and tailored support: Toombs, 2021; Vitopoulos, 2018 Seek to minimise distrust, lack of motivation/readiness to receive support Clifasefi, 2016; Cole, 2017; de Vet, 2017; Montgomery, 2017; Stanhope, 2007; Vitopoulos, 2018; Zerger, 2016
Relationship with case manager 10 studies: Adame, 2020; Chinman, 2000; Clifasefi, 2016; Cole, 2017; de Vet, 2017; Jost, 2014; Montgomery, 2017; Ponce, 2018; Roberts, 2012; Stanhope, 2007	Support positive client‐case manager relationships: Adame, 2020; Chinman, 2000; Clifasefi, 2016; Cole, 2017; de Vet, 2017; Jost, 2014; Montgomery, 2017; Ponce, 2018; Roberts, 2012; Stanhope, 2007
Providing information to clients to facilitate engagement 6 studies: Blosnich, 2020; Cole, 2017; Montgomery, 2017; Ponce, 2018, Roberts, 2012; Tsai, 2014	Adequate information around programmes and available support: Montgomery, 2017; Ponce, 2018; Tsai, 2014
Case manager commitment 5 studies: Chinman, 2017; Choy‐Brown, 2021; de Vet 2017; Miller, 2001; Quinn, 2018	CM understanding of evidence base: Perception of intervention being beneficial to clients: Chinman, 2017; de Vet, 2017 Reconciliation of intervention with personal beliefs (e.g., harm reduction conflicting with Housing First principles): Choy‐Brown, 2021; Quinn, 2018
**Agency support for case managers and CM teams**	
Emotional skills and training needs of case managers 15 studies: Blosnich, 2020; Chinman, 2017; Choy‐Brown, 2021; Clifasefi, 2016; Cole, 2017; de Vet, 2017; Fleury, 2014; Francis, 2000; Miller, 2001; Montgomery, 2017; Ploeg, 2008; Stanhope, 2007; Stanhope, 2015; Stergiopoulos, 2012; Zerger, 2016	Nurture CM emotional investment in clients: Francis, 2000; Zerger, 2016 and trust development: Ploeg, 2008; Ponce, 2018; Stanhope, 2007 Develop ‘therapeutic alliance’: Ploeg, 2008; Ponce, 2018; Stanhope, 2007; Stanhope, 2015 Support and training on management of difficult situations to minimise stress/burnout: Clifasefi, 2016; Cole, 2017 Specific training in crisis management, harm reduction and motivational interviewing: Miller, 2001; Montgomery, 2017; Stergiopoulos, 2012
Attracting and retaining staff 12 studies: Austin, 2014; Clifasefi, 2016; Collins, 2019; Fitzpatrick, 2011; Fleury, 2014; Flowers, 2014; Kietzman, 2020; Miller, 2001; Montgomery, 2017; Stergiopoulos, 2012; Toombs, 2021; Vitopoulos, 2018	(Try to address difficulty) of recruiting staff from diverse backgrounds: Clifasefi 2016; Miller, 2001; Stergiopoulos, 2012 Increase funding/pay/status to reduce staff turnover: Fitzpatrick, 2011; Flowers, 2014
Communication and engagement with other agencies 11 studies: Austin, 2014; Choy‐Brown, 2021; de Vet, 2017; First, 1990; Miller, 2001; Montgomery, 2017; Montgomery, 2019; Ploeg, 2008; Ponce, 2018; Shepherd, 2019; Vitopoulos, 2018	Active communication and/or co‐location: Montgomery, 2017; Montgomery, 2019; Ponce, 2018
Managerial support for case managers 5 studies: Chinman, 2017; Choy‐Brown, 2021; Fleury, 2014; Miller, 2001; Rog, 1997	Managers to provide specific support for the work of case managers: Chinman, 2017; Choy‐Brown, 2021; Fleury, 2014; Miller, 2001; Rog, 1997
Tensions around adherence to policies and guidelines—with potential for rule breaking to deliver services 4 studies: Choy‐Brown, 2021; Francis, 2000; Miller, 2001; Quinn, 2018	No specific consensus
**Housing (availability, security and choice)**	
Housing safety, security and choice 12 studies: Adame, 2020; Cole, 2017; Collins, 2019; Fitzpatrick, 2011; Fleury, 2014; García, 2020; Kirst, 2014; Montgomery, 2017; Montgomery, 2019; Roberts, 2012; Stergiopoulos, 2012; Zerger, 2016	Facilitators to safety/security: Stability of housing: Adame, 2020; Kirst, 2014 Formal security measures (e.g., staff, cameras): Cole, 2017; Montgomery, 2017; Roberts, 2012 Maximise security in relation to drugs/violence in the community: García, 2020; Montgomery, 2017; Roberts, 2012 and for women: Cole, 2017; Montgomery, 2017; Roberts, 2012 Assuring privacy: García, 2020; Montgomery, 2017
Landlord relationships 7 studies: Choy‐Brown, 2021; Collins, 2019; Fleury, 2014; Montgomery, 2017; Montgomery, 2019; Ponce, 2018; Zerger, 2016	Recognise importance of landlords’ views and relationship development with case manager: Choy‐Brown, 2021; Collins, 2019; Fleury, 2014; Montgomery, 2017; Montgomery, 2019; Ponce, 2018; Zerger, 2016
Lack of access, to appropriate housing and other resources, as a barrier 6 studies: Adame, 2020; Austin, 2014; Blosnich, 2020; Collins, 2019; Montgomery, 2017; Ploeg, 2008	Assure financial resources for housing and basic items such as furniture, utilities, food: Adame, 2020; Collins, 2019; Montgomery, 2017 Provide funding for transport: Blosnich, 2020; Collins, 2019; Montgomery, 2017
**National Policy and Guidance (interagency working legal frameworks, large scale resource allocation)**	
Agency partnership and collaboration 15 studies: Austin, 2014; Fitzpatric,k 2011, 2014; Flowers, 2014; Ploeg, 2008; Kietzman, 2020; Miller, 2001; Montgomery, 2017; Montgomery, 2019; Newman, 2017; Patel, 2013; Rog, 1997; Shepherd, 2019; Stergiopoulos, 2012; Vitopoulos, 2018	Multi‐agency discussion/management meetings Montgomery 2017; Shepherd 2019; Vitopoulos 2018 Importance of coordination Miller 2001; Patel 2013; Ploeg 2008 Named contact in external partner(s) Austin 2014; Fitzpatrick 2011
Policies and guidelines 8 studies: Fitzpatrick, 2011; Miller, 2001; Montgomery, 2017; Ponce, 2018; Quinn, 2018; Rog, 1997; Stergiopoulos, 2012; Vitopoulos, 2018	No specific consensus
Data monitoring and sharing 8 studies: Austin, 2014; Chinman, 2017; Choy‐Brown, 2021; Fitzpatrick, 2011, Miller, 2001; Montgomery, 2017; Patel, 2013; Ponce, 2018	Establish/agree metrics: Austin, 2014; Miller, 2001; Montgomery, 2017
Funding availability and requirements 8 studies: Choy‐Brown, 2021; Fitzpatrict, 2011; Fleury 2014; Francisn, 2000; Miller, 2001; Ponce, 2018; Quinn, 2018; Rog, 1997	Reduce complexity of requirements, e.g., eligibility for scheme entry: Francis, 2000; Ponce, 2018
Culture and senior commitment 8 studies: Austin et al., 2014; Chinman et al., 2017; Choy‐Brown et al., 2021; de Vet et al., 2017; Fitzpatrick et al., 2011; Flowers et al., 2014; Vitopoulos et al., 2018	Recognise importance of organisational culture and commitment fostered by senior management: Austin et al., 2014; Chinman et al., 2017; Choy‐Brown et al., 2021; de Vet et al., 2017; Fitzpatrick et al., 2011; Flowers et al., 2014; Vitopoulos et al., 2018
Client identification and referral 5 studies: Blosnich, 2020; Fitzpatrick, 2011; Montgomery, 2017; Ponce, 2018; Quinn, 2018	No specific consensus
Alignment with existing procedures 3 studies: Fleury, 2014; Flowers, 2013; Ponce, 2018	Alignment with existing procedures to facilitate programme implementation Fleury, 2014; Flowers, 2013; Ponce, 2018

#### 5.3.10 Face to face practice: Case management components

##### Conditionality (nine studies)

Nine studies reported on issues around the conditionality of service provision (Choy‐Brown, 2021; Clifasefi, 2016; Cole, 2017; Kirst, 2014; Montgomery, 2017; Montgomery, 2019; Roberts, 2012; Stanhope, 2015; Toombs, 2021).

Five studies reported the impact of having conditions attached to service provision from the perspective of clients (Clifasefi, 2016; Cole, 2017; Kirst, 2014; Montgomery, 2017; Roberts, 2012). Clients were wary and reported discomfort when engaging in programmes that had conditions of treatment for substance use (Clifasefi, 2016; Cole, 2017). Programme recipients reported that the number of rules could be a burden, which led to them exiting programmes (Cole, 2017; Montgomery, 2017). Additionally, clients felt their case manager was watching whether they were abiding by the rules, which they disliked, and reported a lack of independence (Cole, 2017; Montgomery, 2017; Roberts, 2012). For clients who had responsibilities, such as employment, the need to engage with case managers and treatment could conflict with their other responsibilities (Montgomery, 2017).

Six studies reported on the effect of having conditions attached to the provision of services from the perspective of case managers (Choy‐Brown, 2021; Cole, 2017; Montgomery, 2017; Montgomery, 2019; Stanhope, 2015; Toombs, 2021). Conditions attached to the provision of case management were reported to be a barrier to clients engaging in services (Choy‐Brown, 2021; Cole, 2017; Montgomery, 2017; Stanhope, 2015). The requirement for clients to live in single‐occupancy housing was a barrier to those who wanted to cohabit with a partner or significant other (Choy‐Brown, 2021). Further, case managers reported that many programme recipients would break rules regarding single‐occupancy and invite other people to live with them, which could lead to evictions (Cole, 2017). Additionally, substance use, refusal to engage with treatment, or long periods of incarceration could lead to clients exiting programmes (Cole, 2017; Montgomery, 2017; Stanhope, 2015). Not having conditions attached to provision was considered to promote client engagement in programmes (Montgomery, 2019; Toombs 2021). In an evaluation of *HUD‐VASH*, housing was not contingent on engaging with services (Montgomery, 2019).

##### Team versus individual case management (seven studies)

Seven studies reported on how the professionals employed to deliver services affected programme implementation (Fleury, 2014; Flowers, 2014; Miller, 2001; Montgomery, 2017; Montgomery, 2019; Ponce, 2018; Vitopoulos, 2018). An evaluation of *Pathways to Independence* highlighted the benefit of including specialists within a team (Ponce, 2018). Team members included a psychologist and health specialists, and case managers were easily able to access appropriate support for clients (Ponce, 2018). Similarly, Montgomery (2019) reported that *HUD‐VASH* had expanded to include specialised staff, such as nurses and employment experts to support clients to achieve successful outcomes. However, it was noted that such staff might not have the appropriate qualifications or experience to support clients (Fleury, 2014; Miller, 2001).

Some studies reported on the inclusion of those with lived experience of homelessness in programme implementation (Flowers, 2014; Montgomery, 2017; Vitopoulos, 2018). Vitopoulos (2018) reported that peer mentors were integrated into case management teams and supported clients in sharing their experiences.

In an evaluation of *HUD‐VASH*, clients discussed the importance of speaking to people who understood their experiences (Montgomery, 2017). Clients appreciated speaking to *Peer Support Specialists* and suggested including more peer mentors on case management teams (Montgomery, 2017). In a study of *Housing First*, recruiting people with lived experience of homelessness to an advisory panel had been difficult due to challenges in defining homelessness (Flowers, 2014). For the *Housing Outreach‐Project Collaborative*, peer volunteers were recruited and staff reported creating a selection criteria and directly targeting appropriate candidates (Vitopoulos, 2018). Peer workers were treated as staff and integrated into programme teams. It was particularly important to provide clear role definitions for peers and clarify their responsibilities to facilitate successful working (Vitopoulos, 2018).

##### Continuity of care (six studies)

Six studies considered continuity of care (Blosnich, 2020; Clark, 2016; de Vet, 2017; Ploeg, 2008; Shepherd, 2019; Vitopoulos, 2018). Having a dedicated case manager was seen to facilitate programme implementation, particularly within the limits of CTI (Clark, 2016). Some participants experienced changes in their case manager, which was perceived as disruptive (Blosnich, 2020). In one study, transgender recipients reported that changes in case management had been difficult with one recipient reporting five changes in their named case manager (Blosnich, 2020). In a study by Shepherd (2019), support workers reported that although recipients had a dedicated case manager, there were frequent changes. This made contact difficult and support workers were sometimes tasked with making clinical decisions about the mental health needs of the recipient.

Continuity of care was seen as important for clients to achieve their goals, and required communication between different service providers (de Vet, 2017; Ploeg, 2008; Vitopoulos, 2018). Case managers delivering CTI discussed continuity of care from shelter programmes to community housing (de Vet, 2017). To ensure a smooth transition for the client, the CTI case manager needed to work with the shelter case manager to complete initial assessments. Where shelter case managers were unavailable, it created a barrier to clients' successful integration into community housing, and made it difficult to adhere to the time limits of the intervention (de Vet, 2017). Case managers in the *Homelessness Intervention Programme* acted as a link between providers when clients were transitioning to different care settings and ensuring that the care plan was communicated (Ploeg, 2008).

##### Professional versus nonprofessional case manager (five studies)

There was no direct evidence relating to whether the case manager should be professionally qualified but six studies noted that a range of skills and experiences were required (Clifasefi, 2016; Collins, 2019; Fitzpatrick, 2011; Miller, 2001; Stergiopoulos, 2012).

Recruiting staff with the requisite skills to deliver case management could be challenging (Collins, 2019). Additionally, it was difficult to recruit staff who could support clients from diverse cultural backgrounds, particularly in regard to language (Clifasefi, 2016; Miller, 2001; Stergiopoulos, 2012). Collins (2019) reported that some staff held biases against service users regarding race, class and disability.

Fitzpatrick (2011) reported using staff turnover as an opportunity to recruit professionals whose values better aligned with the organisational culture and approach to homelessness. In a study of *At Home/Chez* Soi, those providing services directly to clients were recruited via health authorities (Flowers, 2014). In an evaluation of *Housing First Ethno‐Racial ICM*, targets were created to ensure that management and delivery staff were recruited who were representative of the clients they were serving in terms of ethnicity (Stergiopoulos, 2012). This meant staff had an understanding of the lived experiences of clients and contributed to positive relationships (Stergiopoulos, 2012).

##### Case load (five studies)

Five studies explored case load and time (Austin, 2014; Chinman, 2017; de Vet, 2017; Rog, 1997; Vitopoulos, 2018). Three studies discussed the amount of time staff had available and its impact on case management services (Austin, 2014; Chinman, 2017; Vitopoulos, 2018). Case managers reported difficulties in spending the required time to provide therapeutic support to clients due to the other tasks they were required to do. In some cases, cases managers were required to spend time finding appropriate accommodation (Austin, 2014) and recruiting clients into the programme (Vitopoulos, 2018). Further, in an evaluation of *MISSION‐Vet*, the intensive service provision required for the intervention did not align with large caseloads and the pressure to deliver outcomes (Chinman, 2017).

The impact of caseload on programme implementation was discussed in two studies (de Vet, 2017; Rog, 1997). Both studies reported that case managers occasionally had to deliver different interventions to different caseloads of clients. For example, de Vet (2017) reported that case managers had both CTI caseloads and standard case management caseloads. When new CTI clients were accepted, case managers needed to transfer clients receiving standard case management to another member of staff, or were asked to work overtime. Similarly, case managers were also required to provide services for to clients within their standard caseload alongside taking on additional clients in the *Homeless Families Programme* (Rog, 1997).

##### Case manager availability (four studies)

Four studies reported on the accessibility of case management services (Montgomery, 2017; Montgomery, 2019; Toombs, 2021; Vitopoulos, 2018). Having immediate or timely access to case management services was perceived as a facilitator (Montgomery, 2017; Toombs, 2021). In practice, this was achieved by case managers answering phone calls or calling participants back the same day, locating case managers on the same site as clients, not having waitlists for participant enrolment, and not having formal booking systems for appointments (Montgomery, 2017; Montgomery, 2019; Toombs, 2021). Further, Vitopoulos (2018) reported that case managers took arts supplies to clients' home, when clients had difficulties with mobility to ensure they could still participate in activities.

One study reported on the availability of case managers as a barrier to engaging with case management (Montgomery, 2017). Some service users reported that their case managers had low availability and were difficult to reach. Additionally, Participants expected a timely response from case managers and in some cases, their messages were not returned or went unanswered for long periods (Montgomery, 2017).

##### Remote/in person provision (three studies)

The very small number of studies looking at the issue of appointment location (Blosnich, 2020; Montgomery, 2019; Toombs, 2021) suggest that convenience of appointment location is an issue but do not provide any evidence in relation to remote or in‐person meetings. In an evaluation of *HUD‐VASH*, case managers were located on the site where clients lived (Montgomery, 2019). Case managers reported that this allowed them to have a holistic view of clients' well‐being and development. Toombs (2021) reported that group sessions took place at two different sites, which facilitated access for programme recipients.

Appointment location was also reported to be a barrier to programme implementation by Blosnich (2020), where some clients needed to travel long distances to receive services. This was particularly problematic where clients had limited access to transport.

##### Frequency of contact (three studies)

Three studies reported on the impact of the frequency of contact with case managers (First, 1990; Montgomery, 2017; Newman, 2017). Higher contact frequency was seen as beneficial to service users engaging with case management services. First (1990) reported that clients who saw their case manager more frequently were more likely to remain housed than those who had infrequent contact. Additionally, Montgomery (2017) found that more frequent case management meetings led to quicker housing placements for service users. In some cases, service users were able to choose how often they met with their case manager depending on their need. In one such case, service users were able to meet with their case managers daily if required (Newman, 2017). Similarly, staff on the *Homeless Intervention Programme* reported frequent, almost daily, meetings when they first began working with new clients, which helped case managers to establish a good relationship.

Infrequent contact with case managers was seen as a barrier to positive relationships, and in one instance, a service user reported not seeing a case manager for over a year (Montgomery, 2017).

##### Time limit (two studies)

Two studies reported on the impact of the timeframe of support in relation to programme implementation (Clark, 2016; Richardson, 1996). Richardson (1996) reported that the length of time within a programme correlated with successful completion, with clients who spent longer in the programme more likely to complete. In a comparison of CTI and ACT, the limited timeframe for delivery CTI was related to clients progressing quicker to independence (Clark, 2016). However, case managers showed a preference for ACT as they were able to provide follow‐up support to clients for an extended period of time (Clark, 2016).

#### 5.3.11 Face to face practice: Other themes relating to the case manager‐PEH relationship

##### Non‐housing support and training needs of clients (14 studies)

Non‐housing support and training needs were associated with successful engagement with case management programmes and reported in fifteen studies (Choy‐Brown, 2021; Clifasefi, 2016; Cole, 2017; Collins, 2019; García, 2020; Miller, 2001; Montgomery, 2017; Montgomery, 2019; Newman, 2017; Ponce, 2018; Richardson, 1996; Roberts, 2012; Tsai, 2014; Vitopoulos, 2018). The types of non‐housing support that clients received related to education, employment, finance, parenting, independent living skills, and substance abuse (Choy‐Brown, 2021; Clifasefi, 2016; Cole, 2017; Collins, 2019; García, 2020; Miller, 2001; Montgomery, 2017; Montgomery, 2019; Ponce, 2018; Richardson, 1996; Roberts, 2012; Tsai, 2014; Vitopoulos, 2018). Clients also indicated wanting to receive education related to substance abuse, clothing, and medical care (Clifasefi, 2016; Roberts, 2012). One study reported that difficulties accessing the employment market were a barrier to delivering employment services to clients (Choy‐Brown, 2021).

Independent living skills were particularly important for engagement with case management programmes (Cole, 2017; Collins, 2019; García, 2020; Miller, 2001; Montgomery, 2017; Vitopoulos, 2018). Independent living skills related to cleaning, cooking, washing, sleeping in a bed, budgeting, and making appointments (Cole, 2017; Collins, 2019; García, 2020; Miller, 2001). Where clients lacked independent living skills, this could threaten their ability to retain housing and remain in case management programmes (Collins, 2019; García, 2020). The development of independent living skills facilitated clients' engagement with programmes (Montgomery, 2017). Some programmes incorporated classes on independent living skills for clients (Montgomery, 2017; Montgomery, 2019; Ponce, 2018), and case managers made informal assessments and demonstrated appropriate skills to clients (Cole, 2017; Collins, 2019; Vitopoulos, 2018).

##### Community support and development (11 studies)

Community support and development was perceived by staff and clients to support programme engagement and was reported in eleven studies (Adame, 2020; Clifasefi, 2016; Cole, 2017; Fleury, 2014; Francis, 2000; Montgomery, 2019; Ponce, 2018; Roberts, 2012; Stergiopoulos, 2012; Toombs, 2021; Tsai, 2014). Types of community building activities implemented by programmes included meals, arts performances, trips, cooking classes, game nights, exercise classes, volunteering, and community meetings (Adame, 2020; Clifasefi, 2016; Cole, 2017; Montgomery, 2019; Ponce, 2018; Roberts, 2012; Stergiopoulos, 2012; Toombs, 2021). Clients also reported engaging in community building behaviours such as making food for others, checking in on neighbours, and helping neighbours with small tasks (Adame, 2020). A sense of community was perceived to benefits clients, particularly in their transition into housing (Cole, 2017; Francis, 2000). Clients enjoyed events that supported the development of community (Clifasefi, 2016; Cole, 2017; Tsai, 2014). However, some clients were concerned about aspects of social integration and experienced loneliness and loss of belonging when entering case management programmes (Cole, 2017; Fleury, 2014).

Barriers to community development included lack of staff to deliver activities, and issues around substance abuse (Adame, 2020; Clifasefi, 2016; Cole, 2017). Whilst some clients used alcohol and substances to create a sense of community, other clients wanted to avoid substances to progress with their own goals. This impacted clients who did not want with others using substances; these clients sought community elsewhere outside of programmes and developed in their own hobbies and interests (Adame, 2020; Clifasefi, 2016; Cole, 2017).

##### Client confidence in case management programmes and CM (11 studies)

Clients' confidence and acceptance of case management and associated programmes was reported in eleven studies (Adame, 2020; Clifasefi, 2016; Cole, 2017; de Vet, 2017; Kirst, 2014; Montgomery, 2017; Ponce, 2018; Stanhope, 2007; Toombs, 2021; Vitopoulos, 2018; Zerger, 2016). Facilitators of client confidence in case management included perceiving case management programmes as a support system (Adame, 2020; Ponce, 2018), client readiness to receive support and housing (Stanhope, 2007), experiencing reliable programme implementation (Kirst, 2014), and perceiving support as relevant to their current circumstances (Toombs, 2021). Programme flexibility and the ability for clients to tailor the support they received increased the relevance of case management programmes to clients and made them more likely to engage in services (Toombs, 2021; Vitopoulos, 2018).

Barriers to client confidence and acceptance of case management programmes included a lack of trust, disbelief, lack of motivation, difficulties transitioning to living in housing, and clients not being ready to accept support (Clifasefi, 2016; Cole, 2017; de Vet, 2017; Montgomery, 2017; Richardson, 1996; Stanhope, 2007; Vitopoulos, 2018; Zerger, 2016). Clients found it difficult to trust new people and environments, and were also distrustful that they would receive case management and housing services (Cole, 2017; Vitopoulos, 2018; Zerger, 2016). Once clients had received housing, they were concerned that their housing was not secure and could be taken away from them (Cole, 2017). Some clients experienced difficulties transitioning to living in housing after experiencing homelessness (Clifasefi, 2016; Cole, 2017). Clients' responses into moving into housing included disbelief, feeling enclosed and negative emotions (Cole, 2017). Clients not being ready or open to receive case management services was seen as a barrier to programme acceptance (Montgomery, 2017; Stanhope, 2007). For some clients, substance use or other difficulties meant that clients were not open to receiving case management and were a barrier to programme engagement (Montgomery, 2017; Stanhope, 2007). A lack of client motivation to engage with case management services was also a barrier to successful programme implementation (Montgomery, 2017; Richardson, 1996). Some programme recipients also found it difficult to accept support from other professionals aside from case manager (de Vet, 2017).

##### Relationship with case manager (10 studies)

The effect of the client‐case manager relationship on implementation was reported in 10 studies (Adame, 2020; Chinman, 2000; Clifasefi 2016; Cole, 2017; de Vet, 2017; Jost, 2014; Montgomery, 2017; Ponce, 2018; Roberts, 2012; Stanhope, 2007). Positive client‐case manager relationships facilitated programme engagement from both the client and case manager perspective (de Vet et al., 2017, Montgomery and Cusack, 2017). When relationships were positive, clients perceived case managers as caring, supportive, empathetic and compassionate (Adame, 2020; Clifasefi, 2016; Montgomery, 2017; Ponce, 2018; Roberts, 2012; Stanhope, 2007). This allowed clients to develop trust in their case manager and feel comfortable communicating with them (Jost, 2014; Montgomery, 2017). As such, clients also perceived case managers were part of the community, which facilitated programme engagement (Adame, 2020). In a study which gave tenants a choice of case manager, tenants focussed on the personal qualities of case managers that would allow them to develop rapport (Jost, 2014). Chinman (2000) reported the effects of having a relationship with a case manager after 12 months of being in the *Community Care and Effective Services and Supports* programme. Regardless of whether the relationship with the case manager was weak or strong, having a relationship with a case manager was associated with fewer days spent homeless.

Equally, a poor client‐case manager relationship was a barrier to programme engagement and was a reason for programme exits (Montgomery, 2017). Staff also perceived that a lack of connection with a client could be a barrier to effective programme implementation (Stanhope, 2007). When relationships were poor, case managers were perceived as non‐empathetic, impersonal, and unsupportive (Montgomery, 2017). From the clients' perspective, poor relationships could stem from perceived favouritism, miscommunication, unfair evictions, and conflict (Clifasefi, 2016; Cole, 2017). Some clients reported that the amount of engagement that was expected with their case manager was overwhelming and clients felt a lack of privacy, and disrespect (Cole, 2017).

##### Providing information to clients to facilitate engagement (six studies)

The information provided to clients directly affected their engagement with programmes and was reported in six studies (Blosnich, 2020; Cole, 2017; Montgomery, 2017; Ponce, 2018; Roberts, 2012; Tsai, 2014). Providing adequate information about programmes and the support available was a facilitator for client engagement in homelessness programmes and increased client satisfaction (Montgomery, 2017; Ponce, 2018; Tsai, 2014). Information was provided to clients directly via case managers, and through orientation sessions, housing lists, and resource lists (Montgomery, 2017; Ponce, 2018).

When participants need for information was not met, this was a barrier to engagement in programmes (Blosnich, 2020; Cole, 2017; Montgomery, 2017; Roberts, 2012). Programme recipients reported a lack of promotion about available programmes resulting in limited awareness amongst potential clients (Cole, 2017; Montgomery, 2017). Programme recipients were also frustrated when case managers provided unclear information, or were unable to answer their questions regarding the programme (Blosnich, 2020; Montgomery, 2017). In some instances, that limited information provided by case managers was seen as a contributor to clients exiting programmes (Montgomery, 2017). Clients specifically were dissatisfied with information provided about how to live in housing and the conditions surrounding their lease (Cole, 2017). Although some clients attended orientation, they found it difficult to understand the information presented due the pace of the session and complexity of programme elements (Montgomery, 2017). Roberts (2012) reported that clients perceived a lack of information about additional services and resources they could receive.

##### Case manager commitment (five studies)

The commitment of case managers affected the implementation of programme delivery and was associated with client outcomes (Chinman, 2017; Choy‐Brown, 2021; de Vet, 2017; Miller, 2001; Quinn, 2018). The commitment of case managers to clients was perceived to facilitate the ability of clients to achieve their goals (Miller, 2001).

Case managers' perception of whether an intervention was beneficial to clients affected the commitment and adherence to certain approaches (Chinman, 2017; de Vet, 2017). In an evaluation of *CTI*, case managers sometimes increased the intensity of services where they should have been decreased, or omitted the transfer‐of‐care meeting (de Vet, 2017). Similarly, in an evaluation of *MISSION‐*VET, case managers perceived that the intervention didn't support clients to meet their needs (Chinman, 2017). This negative perception of the intervention hindered implementation of the programme.

Two studies reported that case managers found it difficult to deliver services according to *Housing First* principles due to their own personal beliefs (Choy‐Brown, 2021; Quinn, 2018). Case managers' personal beliefs around harm reduction sometimes conflicted with *Housing First* principles, which made it difficult to ensure that case management services was being delivered consistently to programme recipients (Choy‐Brown, 2021). Additionally, some case managers believed that clients wuld not do well if they would not engage with services (Quinn, 2018).

#### 5.3.12 Local services (supporting staff, supervision, leadership and culture)

##### Emotional skills and training needs of case managers (17 studies)

The emotional skills and associated training needs of case managers were reported in 17 studies (Blosnich, 2020; Chinman, 2017; Choy‐Brown, 2021; Clifasefi, 2016; Cole, 2017; Collins, 2019; de Vet, 2017; Fleury, 2014; Francis, 2000; Miller, 2001; Montgomery, 2017; Ploeg, 2008; Ponce, 2018; Stanhope, 2007; Stanhope, 2015; Stergiopoulos 2012; Zerger, 2016). Case managers perceived that their emotional investment in clients improved access to housing, affected client stability, and reduced the number of programme exits (Francis, 2000; Zerger, 2016). As such, achieving a high‐quality therapeutic alliance with clients was prioritised and case managers reported actively engaging in relationship building and good communication (Ploeg, 2008; Ponce, 2018; Stanhope, 2007; Stanhope, 2015). Case managers discussed the importance of building trust with clients to facilitate engagement with the programme (Ploeg, 2008; Stanhope, 2007). Trust was developed through spending time with clients, listening, displaying empathy, showing hope for clients, discussing aspects of their life not related to case management, and continuing to engage with clients when they were having difficulties (Ploeg, 2008; Ponce, 2018; Stanhope, 2007). In some instances, case managers perceived that clients would try to manipulate them and discussed the need to set clear boundaries (Stanhope, 2007). Additionally, case managers discussed the need to communicate consequences to clients when they violated programme or lease conditions (Stanhope, 2007; Stanhope, 2015). Case managers did not want to be seen as enforcers, and reported explaining the consequences or using persuasion (Stanhope, 2007; Stanhope, 2015).

Case managers also needed to engage with clients who were verbally abusive or violent, which had a high emotional cost (Clifasefi, 2016). These encounters were stressful for case managers, and went alongside interacting with clients with high needs. The stress of working in case management and delivery high intensity services were related to turnover and burnout (Collins, 2019; Fleury, 2014; Francis, 2000). Clients also perceived that staff struggled when difficult situations arose (Clifasefi, 2016). As such, clients suggested that staff should receive training on how to manage difficult situations with clients and appropriate methods of self‐care (Clifasefi, 2016; Cole, 2017). Clients also suggested specific training for case managers in how to work with clients who were transgender, as some had conflicting views when providing services (Blosnich, 2020).

Seven studies reported on the technical skills and training needs of staff to effectively implement case management (Chinman, 2017; Choy‐Brown, 2021; de Vet, 2017; Fleury, 2014; Miller, 2001; Montgomery, 2017; Stergiopoulos, 2012). Training was perceived to facilitate the delivery of case management programmes and create a collaborative environment between staff (Fleury, 2014; Stergiopoulos, 2012). Case managers reported that training in CBT allowed them to deliver therapy to clients at home (Montgomery, 2017). Case managers also perceived that using motivational interviewing was effective at supporting clients (Montgomery, 2017). Use of the *Getting To Outcomes* framework facilitated planning for intervention delivery and helped teams resolve problems (Chinman, 2017). Staff received training through workshops, webinars, coaching sessions, and supervision (Choy‐Brown, 2021; Fleury, 2014). Staff received training regarding the delivery of specific interventions (e.g., ACT, ICM) and implementing *Housing First* principles (Stergiopoulos, 2012). In an evaluation of CTI, de Vet (2017), reported that training was related to staff adherence to the model. Miller (2001) reported that staff required further training in the form of supervision and case managers also requested training in crisis management, harm reduction and motivational interviewing (Miller, 2001 Montgomery, 2017; Stergiopoulos, 2012).

###### Attracting and retaining staff (12 studies)

The impact of staff resources on project implementation was reported in 12 studies (Austin, 2014; Clifasefi, 2016; Collins, 2019; Fitzpatrick, 2011; Fleury, 2014; Flowers, 2014; Kietzman, 2020; Miller, 2001; Montgomery, 2017; Stergiopoulos, 2012; Toombs, 2021; Vitopoulos, 2018).

Barriers to programme implementation included staff recruitment, turnover, understaffing, role definition, and issues related to diversity and inclusion (Austin, 2014; Clifasefi, 2016; Collins, 2019; Fitzpatrick, 2011; Fleury, 2014; Kietzman, 2020; Miller, 2001; Montgomery, 2017; Stergiopoulos, 2012; Toombs, 2021; Vitopoulos, 2018). Recruiting staff with the requisite skills to deliver case management could be challenging (Collins, 2019). Additionally, it was difficult to recruit staff who could support clients from diverse cultural backgrounds, particularly in regard to language (Clifasefi, 2016; Miller, 2001; Stergiopoulos, 2012). Further, Collins (2019) reported that some staff held biases against service users regarding race, class and disability. Austin et al. (2014) reported that the rapid recruitment of staff could put pressure on management structures and led to the need to recruit additional managerial staff. Staff turnover was reported as an issue affecting programme implementation in six studies (Austin, 2014; Collins, 2019; Fitzpatrick, 2011; Fleury, 2014; Kietzman, 2020; Montgomery, 2017). Turnover of case managers was due to the high demands of the work and low pay (Fitzpatrick, 2011). At a case management level, staff turnover affected relationships with clients, learning opportunities within the teams, implementation of specific approaches to homelessness, and ability to meet client demand and need (Fleury, 2014; Kietzman, 2020; Montgomery, 2017). At a leadership level, staff turnover impacted the supervision available to case managers (Fleury, 2014). Due to issues with recruitment and turnover, understaffing and staff disruption became an issue which could affect the clients' stability (Collins, 2019; Fleury, 2014; Montgomery, 2017).

Five studies reported strategies regarding recruitment and retention of case managers (Fitzpatrick, 2011; Fleury, 2014; Flowers, 2014; Stergiopoulos, 2012; Vitopoulos, 2018). Fitzpatrick 2011 reported increasing case manager salaries. Further, Fitzpatrick (2011) reported using staff turnover as an opportunity to recruit professionals whose values better aligned with the organisational culture and approach to homelessness. In a study of *At Home/Chez* Soi, those providing services directly to clients were recruited via health authorities (Flowers et al., 2014). These professionals remained employed by the health authority, which provided benefits in terms of salary and professional status, and helped reduce turnover (Flowers, 2014). The use of buddy systems was reported to help new employees embed into new teams and organisations (Fleury, 2014). In an evaluation of *Housing First Ethno‐Racial ICM*, targets were created to ensure that management and delivery staff were recruited who were representative of the clients they were serving in terms of ethnicity (Stergiopoulos, 2012). This meant staff had an understanding of the lived experiences of clients and contributed to positive relationships (Stergiopoulos, 2012). For the *Housing Outreach‐Project Collaborative*, peer volunteers were recruited and staff reported creating a selection criteria and directly targeting appropriate candidates, which was an effective method of recruitment (Vitopoulos, 2018). Further, peer workers were treated as staff and integrated into programme teams. It was particularly important to provide clear role definitions for peers and clarify their responsibilities to facilitate successful working (Vitopoulos, 2018).

Two studies reported specifically on issues related to diversity and inclusion and how this affected programme implementation (Stergiopoulos, 2012; Toombs, 2021). One study assessed the *Housing Outreach Program Collaborative*, which was adapted for Indigenous young people in Canada (Toombs, 2021). First Nations were employed to deliver the intervention, which led to the tailoring of services and facilitated implementation. For example, the titles of team members were changed to fit culturally with clients and activities were aligned with their values and traditions (Adame, 2020; Toombs, 2021). Stergiopoulos (2012) evaluated *Housing First Ethno‐Racial ICM*, which employed staff that were representative of the clients they served. Staff were able to use their own lived experiences to help clients discuss issues relating to discrimination as well as creating formal mechanisms for reporting discrimination. Additionally, aligning the intervention to the culture and language of clients created an inclusive environment, which enabled clients to receive appropriate services.

##### Communication and engagement with other agencies (12 studies)

Communication and engagement with other agencies were an important part of case managers' roles and reported in ten studies (Chinman, 2017; Choy‐Brown, 2021; de Vet, 2017; First, 1990; Montgomery, 2017; Montgomery, 2019; Ploeg, 2008; Ponce, 2018; Shepherd, 2019; Vitopoulos, 2018) and shared space was discussed specifically in two studies (Austin, 2014; Miller, 2001). Case managers engaged with several agencies relating to housing, health, dental, clothing, food, transport, finance, education, lawyers, and religious (First, 1990; Montgomery, 2017; Montgomery, 2019; Ploeg, 2008). Effective communication with external agencies was seen as crucial for the success for programme delivery (Chinman, 2017). Case managers were able to leverage additional services for clients and also acted as advocates for clients to receive services (Montgomery, 2017; Montgomery, 2019; Ploeg, 2008; Ponce, 2018) Where difficulties arose in linking clients with services, this was perceived as barrier to clients' success (de Vet, 2017). Reasons for not being able to link clients with services provided by external agencies included lack of available resources, and limited contact between the client and case manager (First, 1990).

Strategies for effective communication between case managers and external agencies included co‐location of services, events to bring providers and agencies together, ensuring that case managers were kept informed of communication with external agencies by copying them into emails, sharing important necessary about clients, and actively building relationships with external providers (Montgomery, 2017; Montgomery, 2019; Ponce, 2018).

In some instances, engaging with agencies could be a barrier to successful programme delivery by case managers (Choy‐Brown, 2021; Shepherd, 2019). In one instance, external agencies created a barrier to programme delivery (Choy‐Brown, 2021). This was due to external agencies disagreement with the programme being delivered according to *Housing First* principals, and withholding housing and services for clients (Choy‐Brown, 2021). Considering services provided by support works, case managers were concerned that services were not being delivered in a manner that would benefit the client (Shepherd, 2019).

Two studies reported on the space required for programme implementation (Austin, 2014; Miller, 2001). In a study of HUD‐VASH, all programme staff were located together to support delivery according to Housing First principles and the work of a multidisciplinary team (Austin, 2014). In this way space acted as a facilitator to programme implementation.

Staff in the *Family‐Community Residence* programme discussed issues related to delivery two distinct programmes (Miller, 2001). Both programmes operated from the same building, but had clients with different needs and delivered different levels of support. Locating the programmes in one building created a barrier in terms of implementation, both due to a lack of space and clients receiving the wrong services. Having additional space would allow both programmes to operate more efficiently. Additionally, there was no staff area onsite, which made it difficult to have confidential conversations and for staff to take breaks away from clients.

##### Managerial support for case managers (six studies)

Support from management for the work of case managers affected programme implementation in six studies (Chinman, 2017; Choy‐Brown, 2021; Fleury, 2014; Miller, 2001; Rog, 1997). In the implementation of *Pathways Housing First*, case managers had frequent meetings with a clinical supervisory group, which facilitated case managers' use of harm reduction principles (Choy‐Brown, 2021). However, supervisors' time was limited and they were concerned that they were not able to fully support case managers due to the large amount of time case managers spent alone with clients in the community (Choy‐Brown, 2021).

In a report by Rog (1997), 31% of case managers wanted more supervision from management, particularly in regard to discussing issues with specific service users. Similarly, case managers working with parents wanted more input from managers about borderline cases of parental abuse, where they were not sure whether a referral to Children's Services was necessary (Miller, 2001).

A lack of access to management staff was a barrier to effective programme implementation for those working in clinical teams in large organisations (Fleury, 2014). In the implementation of *MISSION‐Vet*, case managers reported not being supported by managers in terms of providing feedback on their work or incentives to use the programme (Chinman, 2017). Some CTI case managers reported a lack of organisational support for chart documentation, which led to a duplication of work and lower quality client charts.

##### Tensions around adherence to policies and guidelines (four studies)

Four studies discussed tension between the policies and guidelines that case managers needed to adhere to and the need to provide services to clients (Choy‐Brown, 2021; Francis, 2000; Miller, 2001; Quinn, 2018). Choy‐Brown (2021) reported a conflict for case managers: one the one hand case managers were required to deliver services according to *Housing First* principles; and on the other hand, the funder required clients to undergo a psychiatric assessment to receive services. This tension led case managers to bend or break the rules to deliver services to clients. Case managers reported moving clinical assessments to follow‐up appointments and carefully wording clinical documents (Choy‐Brown, 2021). A similar dilemma existed for case managers in a study reported by Francis (2000). Many potential clients had substance use issues alongside mental health issues. However, to meet the funders' eligibility requirements, clients could not have a primary diagnosis of a substance use issue. In these instances, case managers reported being flexible with the eligibility requirements to allow clients to receive services, and sometimes asked health professionals to provide alternative diagnoses (Francis, 2000).

Miller (2001) reported that clients who were intended to be accepted onto a case management programme for families, were sometimes first offered a place on a programme for singles. This was due to participants not yet being reunited with their children, who were in foster care. Case managers also reported specifically accepting those clients who were more likely to achieve programme outcomes (Quinn, 2018), and overlooking rules regarding second occupants if it would facilitate client outcomes (Choy‐Brown, 2021).

#### 5.3.13 Housing (availability, security and choice)

##### Housing safety, security and choice (12 studies)

Housing security, safety and choice was a theme identified in 12 studies (Adame, 2020; Cole, 2017; Collins, 2019; Fitzpatrick, 2011; Fleury, 2014; García, 2020; Kirst, 2014; Montgomery, 2017; Montgomery, 2019; Roberts, 2012; Stergiopoulos, 2012; Zerger, 2016).

Housing security and choice was reported to affect programme implementation in seven studies (Collins, 2019; Fitzpatrick, 2011; Fleury, 2014; García, 2020; Montgomery, 2017; Stergiopoulos, 2012; Zerger, 2016). In a study of *HUD‐VASH*, clients reported that difficulties with finding suitable housing could lead to programme exit or significant delays in moving into housing (Montgomery, 2017; Stergiopoulos, 2012). Further, Montgomery (2017) reported that those who exited the *HUD‐VASH* programme were more likely to be dissatisfied with housing and the neighbourhood. Issues with housing included poor quality, lack of housing for specific populations (e.g. families), available housing being located in areas of high crime, and some landlords not accepting clients with criminal records or poor credit ratings (Fitzpatrick, 2011; García, 2020; Montgomery, 2017). In some instances, poor quality housing meant that programmes did not allow clients to take a lease, which extended the time required to search for housing (Montgomery, 2017). For clients receiving vouchers, there was the risk of losing the housing voucher if they did not find housing within an allotted timeframe, which contributed to programme exits (Montgomery, 2017). Fleury (2014) reported that differences in the perception of housing refusal between case management teams and housing teams could be a barrier to clients finding adequate housing.

Giving clients choice between living in single‐site housing or scattered‐site community housing, was perceived to facilitate programme implementation (Montgomery, 2019). Consumers seeking out housing options through adverts and websites could also facilitate the search for housing (Zerger, 2016). However, client preferences could also be a barrier to finding suitable housing (Collins, 2019; Zerger, 2016). In some cases, clients continued to hold unrealistic expectations for housing in terms of location or quality (Zerger, 2016). Some clients initially accepted housing, which was not their first choice, which could lead to transfer requests and stress (Collins, 2019; Zerger, 2016).

Clients' sense of safety and security affected programme engagement and was reported in six studies (Adame, 2020; Cole, 2017; García, 2020; Kirst, 2014; Montgomery, 2017; Roberts, 2012). Clients perceived that stable housing was associated with feeling safe (Adame, 2020; Kirst, 2014), and was a reason for choosing to engage in programmes (Cole, 2017). Clients perceived that formal security measures, such as security staff and cameras, were a positive aspect of housing and shelter programmes (Cole, 2017; Montgomery, 2017; Roberts, 2012). Some clients struggled in the transition to living in housing due to trust issues after experiencing homelessness, which resulted in some choosing to leave programmes (Cole, 2017).

Feeling unsafe was a barrier to clients continued engagement in programmes and caused some participants to exit programmes (Cole, 2017; Montgomery, 2017; Roberts, 2012). The behaviour of other residents could cause clients to feel unsafe, particularly where drugs and violence were involved (García, 2020; Montgomery, 2017; Roberts, 2012). Women perceived that they were particularly vulnerable and reported feeling threatened or in danger (Cole, 2017; Montgomery, 2017; Roberts, 2012). Privacy was also a concern for clients and a lack of caused some residents to leave programmes (García, 2020; Montgomery, 2017).

##### Landlord relationships (six studies)

The importance of landlords' views and relationship development was a theme identified by seven studies (Choy‐Brown, 2021; Fleury, 2014; Montgomery, 2017; Montgomery, 2019; Ponce, 2018; Zerger, 2016). Landlord relationships were viewed as important to housing stability in one study (Montgomery, 2017). To foster good relationships with landlords, Montgomery (2017) recommended holding fairs to engage with and recruit property owners. The authors also suggested a process of certification for landlords who consistently met guidelines, which could help speed up the process of service users being housed, and debarring those landlords who took advantage of service users.

In five studies, case managers were responsible for engaging with landlords to secure housing for service users (Choy‐Brown, 2021; Fleury, 2014; Montgomery, 2019; Ponce, 2018; Zerger, 2016). Case managers reported building good relationships with landlords by creating easy channels of communication by text (Choy‐Brown, 2021). Case managers needed to advocate to landlords on behalf of service users to ensure they were accepted and remained tenants (Montgomery, 2019), explain the process of accepting vouchers (Fleury, 2014), and troubleshoot any issues (Ponce, 2018). Good relationships with landlords meant they could be willing to lend to service users in the future and facilitate access to housing (Zerger, 2016).

##### Lack of access to appropriate housing and other resources as a barrier (five studies)

Five studies reported clients' lack of access to resources were a barrier to programme participation, particularly in regard to securing appropriate housing (Adame, 2020; Austin, 2014; Blosnich, 2020; Montgomery, 2017; Ploeg, 2008). Both case managers and clients reported that a lack of financial resources frequently created a barrier to securing housing, and obtaining basic items such as furniture, utilities, and food (Adame, 2020; Montgomery, 2017). Further clients sometimes had difficulties maintaining housing due to insufficient funds for rent payments (Montgomery, 2017; Ploeg, 2008). Some programmes also provide move‐in assistance funds to clients, which were perceived to facilitate access to housing (Montgomery, 2017). Programme recipients and case managers also reported that a lack of transport could hinder clients' access to housing (Blosnich, 2020; Montgomery, 2017). A lack of transport made it difficult for clients to attend house viewings, move into properties, and receive services (Blosnich, 2020; Montgomery, 2017). In some instances, case managers were able to provide transport for clients, which facilitated their search for housing (Montgomery, 2017).

#### 5.3.14 National policy and guidance (interagency working legal frameworks, large scale resource allocation)

##### Agency partnership and collaboration (15 studies)

Agency partnership and collaboration were seen as central to effective project implementation in 15 studies (Austin, 2014; Fitzpatrick, 2011; Fleury, 2014; Flowers, 2014; Kietzman, 2020; Miller, 2001; Montgomery, 2017; Montgomery, 2019; Newman, 2017; Patel, 2013; Ploeg, 2008; Rog, 1997; Shepherd, 2019; Stergiopoulos, 2012; Vitopoulos, 2018). Organisations delivering case management interventions in homelessness reported collaborative working with a range of partners which included government departments, non‐governmental organisations, housing authorities, voluntary sector organisations, community organisations, emergency shelters, drug and alcohol treatment centres, family programmes, youth services, landlord associations, property management services, dental services, and homemaking services (Austin, 2014; Fitzpatrick, 2011; Flowers, 2014; Montgomery, 2019; Newman, 2017; Ploeg, 2008; Shepherd, 2019; Stergiopoulos, 2012).

Rog (1997) reported that case management services for the *Homeless Families Programme* were delivered through a number of external providers. However, to retain control over the services delivered, the number of providers was eventually reduced. In an evaluation of *Housing First‐Ethno Racial*, an agency with experience of anti‐racism and anti‐oppression principles were key to the successful implementation of the programme, who supported with supervision and administration (Stergiopoulos, 2012).

Partnership and collaboration were seen to improve care for service users and benefit tenants (Kietzman, 2020). Within an evaluation of homelessness services in Newcastle, collaboration with the Young People's Service, Housing Advice Service, and the Family Intervention Project were associated with a reduction in the number of evictions (Fitzpatrick, 2011). Ploeg (2008) reported that working with homemaking services prevented eviction for elderly service users and allowed them to stay in their homes.

Within multiagency partnerships, several practices were seen as facilitators to programme delivery. Partners having common goals was seen to facilitate programme delivery as individuals were able to operate with understanding and respect (Fitzpatrick, 2011). The separation of case management services and housing services was seen to facilitate alignment with Housing First principles (Montgomery, 2019). This allowed case managers to retain some separation in enforcing any rules related to property and focus on delivery services according to Housing First principles. Engaging in multiagency discussion was seen to facilitate implementation of case management programmes (Shepherd, 2019; Vitopoulos, 2018). Shepherd (2019) reported management meetings that included representative from all key stakeholders, which allowed for open discussion and good quality relationships. Vitopoulos (2018) reported that multiagency discussion resulted in the provision of improved wraparound services for clients and increased access to appropriate services. Montgomery (2017) reported that to facilitate positive working relationships between different partners, HUD‐VASH Boot Camps were established. These were a forum where partners could discuss collaborative approaches to support programme recipients. Further, positive relationships between project co‐ordinators and community partners helped foster good working relationship (Flowers et al., 2014). Having a named contact for external partners could facilitate programme implementation and allow recipients to receive appropriate services in a timely manner (Austin, 2014; Fitzpatrick, 2011).

Four studies reported issues with partnership and collaboration that were barriers to programme implementation (Fleury, 2014; Miller, 2001; Patel, 2013; Ploeg, 2008). Fleury (2014) reported that partners possessing different philosophies and organisational cultures were a barrier to implementation. A lack of co‐ordination between service providers was also perceived to be a barrier to successful outcomes for service users (Miller, 2001; Patel, 2013; Ploeg, 2008). Specifically, lack of co‐ordination with foster care services was problematic in terms of children being reunified with parents in the *Family‐Community Residence* programme (Miller, 2001). Alongside stable housing, one of the programme goals related to reunifying children with parents. However, it could sometimes be difficult to reunify families where children were in stable placements. A lack of co‐ordination with hospital discharge services, application procedures and transport were seen as a barrier to elderly service users being able to stay in their own homes in the *Homelessness Intervention Programme*.

##### Policies and guidelines (eight studies)

Eight studies reported how policies and guidelines impacted programme implementation (Fitzpatrick, 2011; Miller, 2001; Montgomery, 2017; Ponce, 2018; Quinn, 2018; Rog, 1997; Stergiopoulos, 2012; Vitopoulos, 2018). Newcastle County Council and Your Homes Newcastle created the ‘Preventing Evictions Protocol’, which facilitated service users remaining in housing (Fitzpatrick, 2011). Staff reported that the protocol was clear and supported their work. As a result of the policy, the possibility that service users could be evicted was highlighted quickly and staff could take the necessary steps to support the service user and help them retain their accommodation. Additionally, Newcastle County Council and Your Homes Newcastle developed the ‘Allocations and Lettings Policy’, which helped to priorities housing need and aligns with statutory definitions for homelessness (Fitzpatrick, 2011).

In an evaluation of HUD‐VASH, the Houston Housing Authority reported a policy of paying up to 110% Fair Market Rents to facilitate service users moving to housing in appropriate areas (Montgomery, 2017). Housing authorities also created streamlined processes, to ensure service users could access housing quickly. These included the creation of single HUD‐VASH application forms, staff completing applications, and services users being provided with checklists (Montgomery, 2017). In an evaluation of *Housing Outreach Project‐Collaborative*, staff reported confusion over the process regarding service users in crisis contacting them out of office hours (Vitopoulos, 2018). As a result, a formal policy was developed and peer‐workers were provided with out of hours telephone numbers to contact the relevant clinical staff.

Considering guidelines in terms of case management approach, Rog (1997) reported that guidelines were derived from the *Homeless Families Program* National Program Office, who provided expectations on caseload and case management model. Quinn et al. (2018) reported a cultural change in informal guidelines regarding programme outcomes. Whereas previously staff suggested that service users could remain in the programme on a permanent basis, they refocussed their approach on moving recipients towards independence.

Ponce (2018) reported issues with communication of internal policies regarding whether programme recipients would receive housing vouchers. This lack of communication of internal policies created a barrier to programme delivery in terms of recruitment of recipients.

Three studies reported suggestions for policies and guidelines that might facilitate service users' access to appropriate housing. Montgomery (2017) suggested using a Vulnerability Index to assess which programmes would be most applicable to service users. Stergiopoulos (2012) suggested developing guidelines to engage hard to reach service users, and the development of anti‐racist strategies to support programme delivery. Regarding situations involving inappropriate parental behaviour, Miller (2001) suggested creating guidelines for staff to assess how to manage the situation and whether it was appropriate to involve Children's Services.

##### Data monitoring and sharing (eight studies)

Eight studies reported data monitoring and sharing process that impacted on programme implementation (Austin, 2014; Chinman, 2017; Choy‐Brown, 2021; Fitzpatrick, 2011; Miller, 2001; Montgomery, 2017; Patel, 2013; Ponce, 2018). In a study on *HUD‐VASH*, national performance metrics became a central concern for leaders (Austin, 2014). This was particularly the case when these performance metrics were not being met, which led to discussions between management and delivery staff. Consequently, staff reported focussing on recipients who were easier to house, rather than those who had greater vulnerabilities and may take longer to house; an unintended consequence. In regard to *HUD‐VASH* programmes, Montgomery (2017) reported a tension between the performance metrics used by HUD and those used by VA. Where housing authorities measured success by the number of recipients who leased housing, VA measured success by the number of recipients enroled on the programme. The tension in using these different metrics led to an issue for one Housing Authority where they did not receive enough referrals for housing meaning they could not use all the housing vouchers. Housing authorities suggested a need to improve data sharing between HUD and VA to ensure that programmes could meet their goals (Montgomery, 2017).

Similarly, Choy‐Brown (2021) reported that management staff used data monitoring to assess whether delivery staff were implementing harm reduction principles. Where harm reduction was not being implemented, contact could be made with team leaders to rectify any issues.

One study reported data monitoring conducting by Advice and Support Workers (Fitzpatrick, 2011). Although they were able to provide evidence for the effectiveness of the work they had done with service users (e.g., tenancy sustainment), other stakeholders were not convinced about their role within the programme and did not value their work.

Care teams reported that data monitoring tools could be useful in co‐ordinating care for recipients (Patel, 2013). Chinman 2017 reported that data reports could be useful to inform team discussions.

One study reported that not having a method for monitoring programme outcomes was a barrier to success (Miller, 2001). Particularly, the *Family‐Community Residence* programme involved children and there was no indication of follow‐up outcomes related to the children involved. Additionally, the programme lacked a method for assessing when adult recipients were able to move to more independent housing and follow‐up data for service users who had been involved in the programme.

Ponce (2018), reported that the monitoring requirements of the funder supported staff to help clients achieve their goals. However, staff also felt that working towards having a high number of client contacts made staff too outcomes focussed and less focussed on the individual.

##### Funding availability and requirements (eight studies)

Obtaining adequate funding and meeting funder and contracting requirements are issues. The requirements of funders were frequently seen as a barrier to effective intervention implementation (Choy‐Brown, 2021; Fleury, 2014; Francis, 2000; Ponce, 2018; Rog, 1997). In an evaluation of *Housing First*, tension existed between the principles of HF and the requirements of the funders (Choy‐Brown, 2021). Within HF, housing is provided unconditionally and does not depend on service users' engagement with treatment. However, the funders required service users to meet with a psychiatrist to be eligible for housing. A further source of tension was created due to funders' requirement for single‐occupancy housing. Choy‐Brown (2021) reported that this requirement presented a barrier to service integration.

Francis (2000) evaluated a government‐funded programme, which was integrated into a county system. This programme encountered barriers at both the federal and county levels regarding eligibility of service, which effected service delivery; residents were frequently excluded from participating in aspects of the intervention due to requirements regarding being housed, substance abuse or mental illness.

In an evaluation of *Pathways to Independence*, the requirement for service users to meet the government criteria for chronic homelessness also created barriers to accessing services (Ponce, 2018). Where staff perceived that there were people who could benefit for the programme, the requirement for chronic homelessness made them ineligible.

Additionally, the monitoring requirements of funders were perceived as burdensome by staff in some cases, which limited the time they had to engage with service users (Choy‐Brown, 2021). Multiple funders also increased the complexity of project delivery, which was a barrier to implementation (Fleury, 2014).

Only one study reported potential facilitators to programme delivery arising from agency contracting (Rog, 1997). This study reported incentives provided by programme funders to agencies providing case management services, which included payment for case management services and providing Section 8 certificates to service users.

##### Culture and senior commitment (eight studies)

The organisational culture and commitment fostered by management staff was a theme in eight studies (Austin, 2014; Chinman, 2017; Choy‐Brown, 2021; de Vet, 2017; Fitzpatrick, 2011; Flowers, 2014; Vitopoulos, 2018). In five studies, this was seen to facilitate programme effectiveness in five studies (Austin, 2014; Choy‐Brown, 2021; de Vet, 2017; Fitzpatrick, 2011; Vitopoulos, 2018). Austin (2014) suggested that the commitment of midlevel managers facilitated programme success. Midlevel managers played an important role in communicating and advocating the programme with senior leaders, frontline staff, and community members, which led to programmes achieving their goals. In an evaluation of *Housing Outreach Project‐Collaborative (HOP‐C)*, Vitopoulos (2018) reported on the importance of management ensuring that everyone understood their role within the project and bringing all the organisations involved together.

A lack of organisational culture made it difficult to ensure consistent programme delivery across staff. Choy‐Brown (2021) reported that the geographical dispersion of frontline staff made it difficult to create a cohesive organisational culture.

Two studies reported how commitment had been developed amongst management level staff (de Vet, 2017; Fitzpatrick, 2011). de Vet (2017) reported that site visits were conducted to develop managers' commitment to the project. Fitzpatrick (2011) suggested that the change in approach from crisis management to prevention, had led to a change in organisational culture and commitment from management staff. Due to taking a preventative approach, staff were able to work with greater flexibility and took a more supportive approach to the work they undertook.

Five studies demonstrated that commitment of senior leaders to interventions facilitated project implementation (Austin, 2014; Chinman, 2017; de Vet, 2017; Fitzpatrick, 2011; Flowers, 2014). In an evaluation of *Housing First (HF)* across multiple sites in the USA, Austin 2014, found that buy‐in from senior leaders supported engagement with government and community organisations. Fitzpatrick (2011) reported that commitment from senior leaders was essential to the success of interventions delivered by Newcastle County Council and Your Homes Newcastle. Strong leadership led to buy‐in from staff across the organisation, increased engagement with politicians and an increase in resources. Similarly, in an evaluation of *At Home/Chez Soi*, the commitment and knowledge of the site coordinator facilitated engagement with government officials, and contributed to positive changes in the provision of support available to service users (Flowers, 2014). Additionally, the enthusiasm of the site co‐ordinator helped to bring stakeholders together. In an evaluation of *MISSION‐Vet*, Chinman (2017) found that a lack of commitment to the intervention from senior leaders hindered implementation across multiple teams.

Several methods of encouraging buy‐in from senior leaders were reported. In an evaluation of CTI in the Netherlands, de Vet (2017), reported how an academic partner organisation secured the commitment of the senior leaders through early consultation, frequent visits, open discussions, and engagement at conferences and workshops. Fitzpatrick (2011) reported that a cultural shift had been stimulated by an unfavourable evaluation by the UK Department for Communities and Local Government (DCLG). The evaluation had led to the organisation pivoting their approach towards prevention and changing framework.

##### Client identification and referral (five studies)

Five studies reported that service users were often referred to case management services and housing programmes via other organisations (Blosnich, 2020; Fitzpatrick, 2011; Montgomery, 2017; Ponce, 2018; Quinn, 2018). For example, service users for *HUD‐VASH* learned about the programme through shelters, rehabilitation groups, or other services they received such as counselling (Blosnich, 2020; Montgomery, 2017). In the *Homeless Intervention Programme*, a programme for the elderly, alongside services users being referred by organisations such as care homes and community support services, recipients could also make self‐referrals or be referred by family and friends. In a study of PTI case management services, one recipient had received a referral letter whilst staying at a shelter (Ponce, 2018). Fitzpatrick (2011) reported that there were gaps in the referral process, particularly in regard to young people who were not known to Young People's Services. These gaps in the referral process were a barrier to programme entry for some young people.

In a study of supportive housing programmes, Quinn (2018) reported that a referral system had been created in Chicago (Chicago's Central Referral System) to support referral into a variety of programmes. This meant that multiple agencies could co‐ordinate referral into case management and housing programmes, particularly for the most vulnerable service users. However, it was noted that there were difficulties in matching service users with the most appropriate programme, and sometimes referrals were made to programmes that did not meet the needs of the recipient.

One study reported issues with recipient identification that could be a barrier to service users accessing appropriate housing in Chicago (Quinn, 2018). Housing programmes were responsible for identifying recipients and often engaged with potential recipients via homeless shelters, social services or medical service providers. However, some potential recipients had disengaged from services and were hard to reach.

##### Alignment with existing procedures (three studies)

Alignment with existing procedures was reported to facilitate programme implementation in three studies (Fleury, 2014; Flowers, 2014; Ponce, 2018). In an evaluation of *At Home/Chez Soi*, Fleury (2014) reported that familiarity with Housing First facilitated programme delivery. In a second evaluation of *At Home/Chez Soi*, the site co‐ordinator sought to align the project with the current government structures and social service provision to ensure service delivery past the research phase of the programme (Flowers, 2014). The integration of new PTI services into existing case management services within *Pathways to Independence* caused barriers to effective programme implementation (Ponce, 2018). Staff were concerned about delays in clients receiving PTI services and some staff perceived that only high‐function clients were receiving PTI services.

#### 5.3.15 Synthesis of results

Taking the findings from the intervention and implementation studies together (see Effects of Interventions and Process and Implementation Synthesis) there are a number of implications that policy makers and practitioners might consider, of specific relevance to the components of case management. The integrated findings, based on the sequential process of juxtaposing findings in a matrix, one of the integration methods discussed in Harden (2018), are summarised in Table 2.

**Table 2:** Synthesis of results—Summary of findings relating to case‐management components with potential implications for policy (for outcomes 12 months or longer).

Intervention findings (housing outcomes)	Intervention findings (mental health outcomes)	Implementation themes
**Case management type**		
Housing First (multi‐component including ACT or ICM) > CM	Variable outcomes but no evidence of an effect across the full body of evidence	No relevant evidence
**Team versus individual case manager**		
Team = individual CM approach	No evidence of an effect	Some support for including clinical specialists and peers with lived experience
**Type of case manager (professional, non‐professional, lived experience)**		
Too few studies	Too few studies	No evidence re professional qualifications but a recognition of a need for CMs able to support clients from diverse cultural backgrounds
**Conditionality**		
Too few studies	Too few studies	Minimise conditionality
**Continuity**		
No dedicated manager > Named manager	No evidence of an effect of either	Case manager continuity desirable
**Case load**		
No evidence of an effect	No evidence of an effect	No evidence re caseload size but the time needed for intensive case management is noted
**Frequency of contact**		
Too few studies	Too few studies	Few studies but frequency of contact correlated with better housing outcomes and improved CM‐client relationship
**Case manager availability**		
Too few studies	Too few studies	Provide timely response to clients
**Time limit**		
Greater impact in the medium compared to long‐term	No evidence of an effect	Too few studies
**Remote versus in‐person support**		
In‐person more effective than mixture of in‐person and remote	No evidence of an effect	No evidence re remote versus in‐person support but the convenience of appointment location noted
**Arranging versus referral**		
Two few studies	Too few studies	No relevant evidence
**Complexity of need**		
Greater impact on clients with high compared to medium complexity of need	No evidence of an effect	No relevant evidence

When considering the findings from the intervention and implementation studies together, a number of observations may be made:

1. From the statistical analysis of the intervention studies, all types of case management result in positive housing outcomes when compared to usual care, with a trend for the more intensive programmes to have better outcomes than standard case management.
2. Other potential benefits are small or null and there is an overall lack of effect on mental health outcomes.
3. There is a suggestion, from the interventional research, that no continuity of case manager may result in improved outcomes compared to continuity whereas evidence from the implementation research opposes this finding with evidence that case management continuity is desirable. There is no clear evidence‐based explanation for this difference.
4. A range of themes were noted from the qualitative analysis that relate to the case manager‐client face‐to‐face practice and the broader context of CM programmes. These could not be explored within a statistical analysis but have clear implications for policy and practice. These include provision for: the non‐housing support and training needs of clients; community support and development for newly housed clients; emotional support and training needs of case managers; housing safety, security and choice. At the national level, consideration should be given to inter‐agency partnership and collaboration via shared policies/guidelines, data sharing and top level commitment (see Table 1).
5. Housing First is a multicomponent intervention that offers more than case management. This could explain the finding that the overall benefits may be greater than other types of case management. It is notable that, of the five principles adopted within Housing First (see Figure 1) four were identified as key themes from the qualitative analysis: No conditionality/readiness conditions, offer choice, an individualised and personalised approach, support community building. The latter two are also supported by the suggestion from the intervention studies that in‐person rather than remote contact is preferable.

## 6 DISCUSSION

### 6.1 Summary of main results

Overall findings from this mixed methods review are summarised within the Synthesis of Findings and have clear implications for case management for PEH (see Table 2).

As well as supporting the multi‐component intervention of Housing First, the findings suggest that the team approach of Assertive Community Treatment may result in better housing outcomes than the individualised approach of Intensive Case Management. Since the case management component is broadly the same for each of these interventions (see Figure 1) one might speculate that the inclusion of additional, clinical or other support may lead to a more intensive intervention which is particularly advantageous for PEH with a higher complexity of need. The finding that case management programmes have a greater impact on clients with high compared to medium complexity of need may lend support to this theory.

Fidelity measures for a particular type of case management programme will define the components of that programme. ACT fidelity measures (Teague, 1998) stress the importance of personalised support (which may be facilitated with a multi‐disciplinary team), as well as community‐based and open‐ended support. No fidelity measures for ICM were identified by the review group and these would seem to be a necessary precursor to ensure that these two approaches (to individual or team managed case management) and their components can be compared, and the findings used to develop practice. The conflicting findings relating to continuity, where there is no clear evidence for ensuring individual case‐manager continuity, could also be suggestive of favouring a team approach but this is conjecture.

Overall, from the meta‐analyses, we found that case management of any description was superior to usual care for outcomes assessing both homelessness and capabilities and wellbeing. We did not find evidence that case management was more or less effective than usual care on outcomes related to mental health, substance use, physical health, and employment. Homelessness and mental health were addressed in the most studies and allowed the exploration of case management types and components.

For homelessness outcomes, case management type was important. Housing First (a multi‐part intervention) had the largest observed impact, followed by Assertive Case Management, Critical Time Intervention and finally Intensive Case Management (albeit the only statistically significant difference was between Housing First and Intensive Case Management).

The results for mental health outcomes were characterised by an overall lack of effect across all case management types but it should be noted that there were variations in outcomes across the body of studies and case‐management models (and usual care) are not consistent across countries.

There were not enough studies to explore differences in homelessness and mental health outcomes for all case management components. The findings from the analyses are summarised very briefly below. Contextual detail for each included study is provided in Supporting Information: Appendix 3. Although considered individually, it is recognised that many of these case management components are closely related and likely to be confounded with one another.

**Table:** 

Component	Homelessness	Mental Health
Case management type	Trend for Housing First (multi‐component including ICM or ACT) > ACT > ICM	No evidence of an effect across body of studies
Team/individual	Team~Individual	No evidence of an effect
Professional case manager	Too few studies	Too few studies
Conditionality	Too few studies	Too few studies
Continuity	No dedicated manager > Named manager	No effect of either
Caseload	No evidence of an effect	No evidence of an effect
Frequency of contact	Too few studies	Too few studies
Case manager availability	Too few studies	Too few studies
Time limit	Medium > long	No evidence of an effect
Remote/in‐person	In‐person > Mixed	No evidence of an effect
Arranging/referral	Too few studies	Too few studies
Complexity of need	High > Medium	No evidence of an effect
Percentage female	No effect	Very slight (non‐significant) positive trend

### 6.2 Overall completeness and applicability of evidence

This was a very large mixed methods review based on an extensive and systematic search for relevant studies, all of which concerning case management interventions for PEH.

### 6.3 Quality of the evidence

A large body of studies were identified looking at case management effectiveness for PEH based on a comprehensive search for studies that included a randomised, or matched, comparison group. In additional the review included a large and representative sample of qualitative and other studies looking at factors that may impact on programme delivery and outcomes.

The majority of studies were assessed as low quality (see Risk of Bias in Included Studies) and this assessment was affected by two major issues. Many intervention studies of otherwise high quality did not provide enough information to show losses to follow up were acceptable at the end of the intervention leading to a low quality outcome based on the critical appraisal tool (White, 2019). Many implementation studies of otherwise high quality did not describe the relationship between the researchers and participants (reflexivity) and any steps taken to mitigate any potential for researcher bias (White, 2018a).

The majority of the evidence base (both intervention and implementation studies) comes from the North American continent and contexts of usual care and case management interventions vary hugely across countries. This should be borne in mind when considering the relevance of the evidence for contexts outside North America.

### 6.4 Potential biases in the review process

There are some issues that may have potentially affected the review analysis; notably some methodological shortcomings in some of the studies and the heterogeneity of studies included in individual meta‐analyses(see Assessment of risk of bias in included studies). Other potential sources of bias are unlikely to have affected findings. The majority of studies were funded by public bodies who are very unlikely to have influenced study methodology or outputs (see Supporting Information: Appendices 3 and 4) and there is no indication that publication bias may have influenced the meta‐analyses (see Figure 6). The consistency of findings for the homelessness outcomes within the intervention studies, and for the major themes identified in the implementation studies, suggest that there can be some confidence in the direction of findings. Across the whole body of evidence, there appears to be no benefit in terms of mental health outcomes over usual care but there was some considerable variation in outcomes across studies.

In terms of case management components, these were considered individually, but may be closely related to one another. There was not enough evidence to be able to correlate any inter‐relationships. In addition, the definitions of case‐load and time‐limit of provision were necessarily broad, and it is possible that a trend may exist that was not identified within the current analysis.

The case management type, as described within the included studies, was not always clear. This is particularly true at the ACT/ICM boundary (give examples) where the review team had to make some assumptions (e.g. if intervention was described as ICM but delivered by a multidisciplinary team a note was made).

Similarly, definitions for frequency of contact as defined by experts for this review as very frequent (≥8 times/month), frequent (4–7 times/month), medium (2–3 times per month) and occasional (≤once per month) to not directly with other research such as Lukersmith (2016) where intensity (albeit to explore case management in brain injury and not homelessness) was defined as high (>3 contacts per week), medium (1–3 times/week) and low (< 3 times/month) making it difficult to compare findings directly with other studies in related areas.

The themes from the framework synthesis are presented in a narrative form. Although the number of studies identifying each theme are noted this should not infer a hierarchy in terms of the confidence that can be placed in the relevance of each finding to any particular context.

### 6.5 Agreements and disagreements with other studies or reviews

Comparing the findings of this review with other reviews (Munthe‐Kaas, 2018; Ponka, 2020) the housing outcomes are broadly similar in terms of the likely impact of any case management intervention on housing outcomes. This lends strength and credibility of the findings while adding in additional detail on the specific types and, to some extent, the components of case management that are likely to be most effective according to the current evidence base. As with previous reviews, the dearth of studies carried out outside the USA is striking and a clearly a recommendation for further research within other settings. A more recent review (Moledina, 2021) also supports the finding that permanent supporting housing interventions have significant benefits on housing stability as compared to usual care while most studies found no significant benefit on mental health outcomes.

Moledina (2021) also included an assessment of cost‐effectiveness studies but a very broad range of studies were included and not all included case‐management or a matched comparison group. In keeping with findings from this review Moledina (2021) concluded that the economic implications of case management interventions are highly uncertain and that case management approaches resulted in higher costs that may be cost‐offset by reductions in other services. In contrast to our findings, they concluded that ACT and ICM interventions may save tax payers money.

Overall, in terms of implementation of case management for PEH, there are many themes from this mixed methods review that resonate with themes from other research (Milaney, 2011; Ponka, 2020; Stergiopoulos, 2014; White, 2019). These shared recommendations are summarised in Table 3.

**Table 3:** Implementation themes in common with other published research.

Coordinated social support and healthcare access	Collaboration and cooperation—A true team approach at national and (inter) agency level
Client‐centred approach	Flexible
	Tailored
	Intensive
Communication and relationships	The right kinds of relationships/advocacy
	Recruiting and retaining case managers
Support and Training	Employment education and skills for clients
	Social/community integration support for clients
	Support and training of case managers

## 7 AUTHORS' CONCLUSIONS

### 7.1 Implications for practice

There are a number of implications from this review that policy makers might consider to be of relevance at national, local agency and face‐to‐face practice levels. At this stage, there is no evidence that case management can be recommended to improve the mental health of PEH; However, there are clear potential benefits in terms of housing outcomes and capabilities and wellbeing.

Implications for practice are outlined in Table 2. These are not recommendations but rather some prompts to help implementers consider the review findings within their own contexts.

### 7.2 Implications for research

Based on the findings from this review there are a number of recommendations for further research:

- Clarify the differences between the Intensive Case Management and Assertive Community Treatment approaches with publication of fidelity measures for ICM and clear selection criteria for each approach.
- New research, or analysis of existing studies, to further explore the effects of ICM, ACT and CTI on PEH groups with medium and high complexity of need.
- Explore variations of Housing First (ICM and ACT) and case‐management (and usual care) contextual variations across countries in relation to mental health, substance use, physical health, and employment outcomes. There is not currently enough evidence to assess whether case management is *ineffective* for all these outcomes in all contexts.
- Expand the research base outside North America.
- Explore costs as well as effectiveness within research studies and enhance the evidence for specific outcomes such as cost per day of stable housing for each case management approach.
- Improve methodological reporting within published research studies. In particular, to provide information on the composition of usual care as a control, and data on drop outs (attrition) in intervention and control groups for intervention studies and to consider the potential for researcher bias (and mitigations to address this) in qualitative research studies exploring implementation of case management interventions.
- Consider the use of additional tools such as GRADE (Guyatt, 2008) and GRADE‐CERQual (Lewin, 2018) in any update of this review to further assess the confidence that one might have in the individual findings across the body of intervention and implementation evidence.

## CONTRIBUTIONS OF AUTHORS

- Content: Ian Thomas, Ben Hannigan, Robin J Smith
- Systematic review methods: Alison L. Weightman, Mala Mann, Simone Willis, Rhiannon Cordiner
- Statistical analysis: Mark Kelson
- Information retrieval: Mala Mann, Lydia Searchfield

## DECLARATIONS OF INTEREST

None to declare.

## PLANS FOR UPDATING THIS REVIEW

We will update this review if a significant new body of research is available and funding is secured.

## PRELIMINARY TIMEFRAME

Approximate date for submission of the systematic review. July 2022.

## DIFFERENCES BETWEEN PROTOCOL AND REVIEW

One of the databases that we proposed to search (NHSEED) was not included in error and citation tracking forwards for each included study was precluded given the huge size of the evidence base identified (105 studies). Reference lists of all included primary studies were reviewed for additional studies along with reference lists of all systematic reviews identified within the search. These methods, identified a number of sibling papers of included studies (particularly economic analyses) but no further primary studies. This suggested that the search strategy already had good sensitivity and the authors are confident that review quality is unlikely to have been affected.

Due to the very large number of intervention papers included it was not possible to contact authors to obtain additional data if study reports did not contain sufficient data to allow calculation of effect size estimates. In these cases we employed standard methods to calculate a standardised mean difference from reported statistics or graph. Where no information was forthcoming the study was not included in meta‐analysis but in a narrative synthesis (see Dealing with missing data).

## SOURCES OF SUPPORT

**Internal sources**

- No internal sources of support.

**External sources**

- Centre for Homelessness Impact, UK.

https://www.homelessnessimpact.org/

## Supporting information

Supporting information.

## Footnotes

^1^
See scale at: https://www.mycasat.org/wp-content/uploads/2017/04/act-dacts.pdf

^2^

https://onlinelibrary.wiley.com/page/journal/18911803/homepage/author-guidelines

^3^

*Low complexity*—Where majority of the population has no other specific needs beyond homelessness. *Medium complexity*—Where the majority of the study population has one other additional need (including: psychiatric/mental health needs, substance use disorder, alcohol use disorder, physical health needs or are avulnerable population [e.g., veterans, youth, victim of domestic violence or institutionalised/incarcerated]). *High complexity*—Where the majority of the study population has two or more additional needs (including: psychiatric/mental health needs, substance use disorder, alcohol use disorder, physical health needs or are avulnerable population [e.g. veterans, youth, victim of domestic violence or institutionalised/incarcerated).
