# Supplementary material for: Exploring the effect of case management in homelessness per components: A systematic review of effectiveness and implementation, with meta‐analysis and thematic synthesis
Source: Campbell Syst Rev. 2023 May 17;19(2):e1329. doi: 10.1002/cl2.1329 (PMC10189499; doi:10.1002/cl2.1329)
Supplement: Supplementary file 1 — Supporting information. [file CL2-19-e1329-s001.docx]

# Appendices

## 1 Appendix 1:  Inclusion/Exclusion Summary

|  | **Inclusion criteria** | **Exclusion criteria** |
| --- | --- | --- |
| **Intervention** | Case-management, whereby the case-manager supports the person or household experiencing, or at risk of experiencing, homelessness by facilitating integrated access to health & social services and accommodation support | Case-management where housing support is not part of the intervention |
| **Study type: Quantitative** | Quantitative study designs with a comparison group    Research with data on the costs/cost-effectiveness of interventions and individual components of those interventions. | Designs without a comparison group (eg pre/post-test) or where the comparison group is from another study |
| **Study type: Qualitative** | Qualitative study designs and process evaluations where data are collected on views and experiences of participants or case-managers |  |
| **Population** | Individuals or households who are currently experiencing, or at risk of experiencing, homelessness as defined by the ETHOS typology (FEANTSA 2017) | Populations from countries other than high-income countries (as defined by the World Bank 2020)    Populations living in housing defined as inadequate but not as experiencing, or at risk of experiencing, homelessness. |

##

## 2 Appendix 2:  Search strategy

This case-management specific search strategy supplemented the findings from the Campbell EGM. Search strategies developed in Scopus and Medline (Ovid) and tested for their ability to identify known relevant studies with good sensitivity before adapting the search to the other databases. All search strategies are listed below.

In all, the following databases will be searched: ASSIA [ProQuest], CINAHL [EBSCO], Cochrane Library, ERIC [EBSCO], International Bibliography of the Social Sciences (IBSS) [ProQuest], Medline [OVID], PsycINFO [OVID], Scopus [Elsevier], Web of Science (Science Citation Index & Social Science Citation Index) [Clarivate].

In addition, the web sites explored by White et al (2020) to December 2019 were browsed for any publications in 2020 and 2021 using the search terms "case management" or "assertive community treatment" or "critical time intervention" or "care management" or "care coordination" or "managed care"

Homeless Hub https://www.homelesshub.ca/

European observatory on homelessness https://www.feantsaresearch.org/en/publications

United State interagency council on homelessness http://www.usich.gov/

ETHOS http://ethos.bl.uk/Home.do

WHO ICTRP http://apps.who.int/trialsearch/

Focus on Prevention http://www.preventionfocus.net/

Social Policy and Practice http://www.spandp.net/

FEANTSA https://www.feantsa.org/

National Coalition Homeless https://nationalhomeless.org/

Homelessness Australia https://www.homelessnessaustralia.org.au/

Mission Australia https://www.missionaustralia.com.au/publications/position‐statements/homelessness

National Alliance to end homelessness https://endhomelessness.org/

Institute of global homelessness https://www.ighomelessness.org/

Homelessness link https://www.homeless.org.uk/

Crisis https://www.crisis.org.uk/about‐us/how‐we‐work/

Housing first https://housingfirsteurope.eu/about‐the‐hub/

Canadian Alliance to end homelessness https://housingfirsteurope.eu/about‐the‐hub/

Social work and policy institutes http://www.socialworkpolicy.org/research/homelessness.html

Association of housing advice services https://www.ahas.org.uk/

Centre point https://centrepoint.org.uk/

Homelessness trust funds https://housingtrustfundproject.org/htf‐elements/homeless‐trust‐funds/

Meliville charitable trust https://melvilletrust.org/category/resources‐reports/

Conrad H Hilton foundation https://www.hiltonfoundation.org/priorities/homelessness#resources

Abt Associates https://www.abtassociates.com/

Mathematica https://www.mathematica‐mpr.com/

American Institutes of Research https://www.air.org/

Rand https://www.rand.org/

MDRC https://www.mdrc.org/

**Search Strategies**

**ASSIA [ProQuest]**

((ab(Homeless NEAR/1 (person* OR youth*)) OR ab(evict* OR homeless* OR "housing excl*" OR "residential stability") OR ab((street* OR private OR improvised OR shelter* OR emergency OR temporar* OR insecure OR overcrowded OR precarious OR stable OR marginal* OR Unstable OR Transitional OR Recovery OR "non permanent") NEAR/3 (dwell* OR hous* OR home* OR accommodat*)) OR ab(street NEAR/3 (life OR living OR lives OR youth* OR child* OR people OR person*)) OR ab((runaway* OR "Run away from home" OR "Running away" OR "Ran away" OR "Going missing" OR "Bag lady" OR houseless* OR unhoused OR "without a roof" OR roofless OR (rough NEAR/1 sleep*) OR destitut* OR "Skid row*" OR "sleepers out")) OR ab(("Pathways to Housing" OR "Accommodation based interventions" OR "Transitional living programs" OR "Homeless Veterans Reintegration Program" OR "access program*" OR "Supported Housing" OR "Housing Program" OR "HUD-VASH")) OR ab("Sober Transitional Housing and Employment Project" OR "sober house placement*" OR "Housing ladders" OR "Staircase housing" OR "low threshold housing" OR "housing status") OR ab(shelter* NEAR/2 (based OR housed OR residen* OR temporar*))) AND ab("case manage*" OR "assertive community treatment*" OR "critical time intervention*" OR "care manage*" OR "coordinated care" OR "care coordination" OR "managed care")) OR ab("Housing first" OR "at home chez soi" OR "homebase community prevention program*" OR "mckinney program*")

**CINAHL [EBSCO]**

S14 S12 OR S13

S13 AB "housing first" or "at home/chez soi" or "homebase community prevention program" or "mckinney program"

S12 S8 AND S11

S11 S9 OR S10

S10 AB ("case manage*" or "assertive community treatment*" or "critical time intervention*" or "care manage*" or "care coordination" or "managed care")

S9 (MH "Case Management")

S8 S1 OR S2 OR S3 OR S4 OR S5 OR S6 OR S7

S7 AB ((based or housed or residen* or temporar*) N2 shelter*)

S6 AB homeless* or runaway

S5 AB "Sober transitional housing and employment project" or "sober house placement" or "housing ladders" or "staircase housing" or "low threshold housing" or "housing status"

S4 AB "Pathways to housing" or "homeless veterans reintegration program" or "access program*" or "supported housing" or " housing program*" or "HUD-VASH"

S3 AB (evict* or homeless* or "housing excl*" or "residential stability" or ((street* or private or improvised or shelter* or emergency or temporar* or insecure or overcrowded or precarious or stable or marginal*) N3 (dwell* or hous* or home* or accommodat*)) or (street N3 (life or living or lives or youth* or child* or people or person*)) or runaway* or "Run away from home" or "Running away" or "Ran away" or "Going missing" or "Bag lady" or Houseless* or Unhoused or "without a roof" or Roofless or (rough adj3 sleep*) or Destitut* or "Skid row*" or "sleepers out")

S2 (MH "Homelessness")

S1 (MH "Homeless Persons")

**Cochrane Library**

#1 MeSH descriptor: [Homeless Persons] explode all trees

#2 MeSH descriptor: [Homeless Youth] explode all trees

#3 #1 OR #2

#4 (evict* or homeless* or "housing excl*" or "residential stability" or ((street* or private or improvised or shelter* or emergency or temporar* or insecure or overcrowded or precarious or unstable or marginal* or transitional or recovery or “non-permanent”) adj3 (dwell* or hous* or home* or accommodat*)) or (street adj3 (life or living or lives or youth* or child* or people or person*)) or runaway* or "Run away from home" or "Running away" or "Ran away" or "Going missing" or "Bag lady" or Houseless* or Unhoused or "without a roof" or Roofless or (rough adj3 sleep*) or Destitut* or "Skid row*" or "sleepers out")

#5 ("Pathways to housing" or "Accommodation based interventions" OR "Transitional living programs" OR "homeless veterans reintegration program" or "access program*" or "supported housing" or " housing program*" or "HUD-VASH")

#6 ("Sober transitional housing and employment project" or "sober house placement" or "housing ladders" or "staircase housing" or "low threshold housing" or "housing status")

#7 (homeless* or runaway*)

#8 ((based or housed or residen* or temporar*) adj2 shelter*)

#9 #3 OR #4 OR #5 OR #6 OR #7 OR #8

#10 ("case manage*" or "assertive community treatment*" or "critical time intervention*" or "care manage*" or "care coordination" or "managed care")

#11 #9 AND #10

#12 ("housing first" or "at home/chez soi" or "homebase community prevention program" or "mckinney program")

#13 #11 or #12 with Cochrane Library publication date Between Jan 1990 and Feb 2021

**ERIC [EBSCO]**

S1 "homeless person*" or "homeless youth*"

S2 Homelessness

S3 AB(evict* or homeless* or "housing excl*" or "residential stability" or ((street* or private or improvised or shelter* or emergency or temporar* or insecure or overcrowded or precarious or unstable or marginal* or transitional or “non-permanent” or recovery) N3 (dwell* or hous* or home* or accommodat*)) or (street N3 (life or living or lives or youth* or child* or people or person*)) or runaway* or "Run away from home" or "Running away" or "Ran away" or "Going missing" or "Bag lady" or Houseless* or Unhoused or "without a roof" or Roofless or (rough N3 sleep*) or Destitut* or "Skid row*" or "sleepers out")

S4 AB ("Pathways to housing" or "Accommodation based interventions" OR "Transitional living programs" or "homeless veterans reintegration program" or "access program*" or "supported housing" or " housing program*" or "HUD-VASH")

S5 AB ("Sober transitional housing and employment project" or "sober house placement" or "housing ladders" or "staircase housing" or "low threshold housing" or "housing status")

S6 AB (homeless* or runaway*)

S7 AB (based or housed or residen* or temporar*) N2 shelter*

S8 S1 OR S2 OR S3 OR S4 OR S5 OR S6 OR S7

S9 TX "case management"

S10 AB ("case manage*" or "assertive community treatment*" or "critical time intervention*" or "care manage*" or "care coordination" or "managed care")

S11 S9 OR S10

S12. S8 AND S11

S13 TX ("housing first" or "at home/chez soi" or "homebase community prevention program" or "mckinney program")

S14 S12 OR S13

(All limited publication from January 1990 – February 2021)

**International Bibliography of the Social Sciences (IBSS) [ProQuest]**

S13 S11 or S12

S12 ab(("Housing first" OR "at home chez soi" OR "homebase community prevention program*" OR "mckinney program*"))Limits applied

S11  S9 AND S10

S10 ab("case manage*" OR "assertive community treatment*" OR "critical time intervention*" OR "care manage*" OR "coordinated care" OR "care coordination" OR "managed care")Limits applied

S9 S1 OR S2 OR S3 OR S4 OR S5 OR S6 OR S7 OR S8

S8 ab(shelter* NEAR/2 (based OR housed OR residen* OR temporar*))Limits applied

S7 ab(("Sober Transitional Housing and Employment Project" OR "sober house placement*" OR "Housing ladders" OR "Staircase housing" OR "low threshold housing" OR "housing status"))Limits applied

S6 ab(("Pathways to Housing" OR "Accommodation based interventions" OR "Transitional living programs" OR "Homeless Veterans Reintegration Program" OR "access program*" OR "Supported Housing" OR "Housing Program" OR "HUD-VASH"))Limits applied

S5  ab((runaway* OR "Run away from home" OR "Running away" OR "Ran away" OR "Going missing" OR "Bag lady" OR houseless* OR unhoused OR "without a roof" OR roofless OR (rough NEAR/1 sleep*) OR destitut* OR "Skid row*" OR "sleepers out"))Limits applied

S4 ab(street NEAR/3 (life OR living OR lives OR youth* OR child* OR people OR person*))Limits applied

S3  ab((street* OR private OR improvised OR shelter* OR emergency OR temporar* OR insecure OR overcrowded OR precarious OR stable OR marginal* OR Unstable OR Transitional OR Recovery OR "non permanent") NEAR/3 (dwell* OR hous* OR home* OR accommodat*))Limits applied

S2  ab(evict* OR homeless* OR "housing excl*" OR "residential stability")Limits applied

S1 ab(Homeless NEAR/1 (person* OR youth*))Limits applied

**Medline [OVID]**

1. homeless persons/ or homeless youth/ or runaway behaviour/
2. (evict* or homeless* or "housing excl*" or "residential stability" or ((street* or private or improvised or shelter* or emergency or temporar* or insecure or overcrowded or precarious or stable or marginal*) adj3 (dwell* or hous* or home* or accommodat*)) or (street adj3 (life or living or lives or youth* or child* or people or person*)) or runaway* or "Run away from home" or "Running away" or "Ran away" or "Going missing" or "Bag lady" or Houseless* or Unhoused or "without a roof" or Roofless or (rough adj3 sleep*) or Destitut* or "Skid row*" or "sleepers out").ti,ab,kw OR ((based or housed or residen* or temporar*) adj2 shelter?).tw.kw. OR (temporar* adj2 (accommodat* or home? or hous*)).tw,kw
3. ("Pathways to housing" or "homeless veterans reintegration program" or "access program*" or "supported housing" or " housing program*" or "HUD-VASH").ti,ab,kw.
4. ("Sober transitional housing and employment project" or "sober house placement" or "housing ladders" or "staircase housing" or "low threshold housing" or "housing status").ti,ab,kw.
5. ((based or housed or residen* or temporar*) adj2 shelter*).ti,ab,kw.
6. or/1-5
7. case management/
8. ("case manage*" or "assertive community treatment*" or "critical time intervention*" or "care manage*" or "care coordination" or "managed care").ti,ab,kw.
9. 7 or 8
10. 6 and 9
11. ("housing first" or "at home/chez soi" or "homebase community prevention program" or "mckinney program").ti,ab,kw.
12. 10 or 11

Limit 12 to 1990 to current

**PsycINFO [OVID]**

1. Homeless/ (7251)
2. (evict* or homeless* or "housing excl*" or "residential stability" or ((street* or private or improvised or shelter* or emergency or temporar* or insecure or overcrowded or precarious or unstable or marginal* or transitional or “non-permanent” or recovery) adj3 (dwell* or hous* or home* or accommodat*)) or (street adj3 (life or living or lives or youth* or child* or people or person*)) or runaway* or "Run away from home" or "Running away" or "Ran away" or "Going missing" or "Bag lady" or Houseless* or Unhoused or "without a roof" or Roofless or (rough adj3 sleep*) or Destitut* or "Skid row*" or "sleepers out").ti,ab.
3. (homeless* or runaway*).ti,ab.
4. ((based or housed or residen* or temporar*) adj2 shelter*).ti,ab.
5. ("Pathways to housing" or "Accommodation based interventions" OR "Transitional living programs" or "homeless veterans reintegration program" or "access program*" or "supported housing" or " housing program*" or "HUD-VASH").ti,ab.
6. or/1-5
7. Case Management/
8. ("case manage*" or "assertive community treatment*" or "critical time intervention*" or "care manage*" or "care coordination" or "managed care").ti,ab.
9. 7 or 8
10. 6 and 9
11. ("housing first" or "at home/chez soi" or "homebase community prevention program" or "mckinney program").ti,ab.
12. 10 or 11

Limit 12 to 1990 to current

**Scopus [Elsevier]**

( ( ( TITLE-ABS-KEY ( ( evict* OR homeless* OR "housing excl*" OR "residential stability" ) ) ) OR ( TITLE-ABS-KEY ( ( ( street* OR private OR improvised OR shelter* OR emergency OR temporar* OR insecure OR overcrowded OR precarious OR stable OR marginal* ) near/3 ( dwell* OR hous* OR home* OR accommodat* ) ) ) ) OR ( TITLE-ABS-KEY ( ( street AND near/3 ( life OR living OR lives OR youth* OR child* OR people OR person* ) ) ) ) OR ( TITLE-ABS-KEY ( ( runaway* OR "Run away from home" OR "Running away" OR "Ran away" OR "Going missing" OR "Bag lady" OR houseless* OR unhoused OR "without a roof" OR roofless OR ( rough AND near AND sleep* ) OR destitut* OR "Skid row*" OR "sleepers out" ) ) ) OR ( TITLE-ABS-KEY ( ( "Pathways to Housing" OR "Homeless Veterans Reintegration Program" OR "access program*" OR "Supported Housing" OR "Housing Program" OR "HUD-VASH" ) ) ) OR ( TITLE-ABS-KEY ( ( "Sober Transitional Housing and Employment Project" OR "sober house placement*" OR "Housing ladders" OR "Staircase housing" OR "low threshold housing" OR "housing status" ) ) ) OR ( TITLE-ABS-KEY ( ( ( based OR housed OR residen* OR temporar* ) near/2 AND shelter* ) ) ) ) AND ( TITLE-ABS-KEY ( ( "case manage*" OR "assertive community treatment*" OR "critical time intervention*" OR "care manage*" OR "coordinated care" OR "care coordination" OR "managed care" ) ) ) ) OR ( TITLE-ABS-KEY ( ( "Housing first" OR "at home chez soi" OR "homebase community prevention program*" OR "mckinney program*" ) ) )

**Web of Science (Science Citation Index & Social Science Citation Index) [Clarivate]**

#12  AB=("Housing first" OR "at home chez soi" OR "homebase community prevention program*" OR "mckinney program*")

#11 #10 AND #9

#10 AB=("case manage*" OR "assertive community treatment*" OR "critical time intervention*" OR "care manage*" OR "coordinated care" OR "care coordination" OR "managed care")

#9 #8 OR #7 OR #6 OR #5 OR #4 OR #3 OR #2 OR #1

#8 AB=(shelter* NEAR/2 (based OR housed OR residen* OR temporar*) )

#7 AB=("Sober Transitional Housing and Employment Project" OR "sober house placement*" OR "Housing ladders" OR "Staircase housing" OR "low threshold housing" OR "housing status")

#6 AB=(("Pathways to Housing" OR "Accommodation based interventions" OR "Transitional living programs" OR "Homeless Veterans Reintegration Program" OR "access program*" OR "Supported Housing" OR "Housing Program" OR "HUD-VASH"))

#5 AB=((runaway* OR "Run away from home" OR "Running away" OR "Ran away" OR "Going missing" OR "Bag lady" OR houseless* OR unhoused OR "without a roof" OR roofless OR (rough NEAR/1 sleep*) OR destitut* OR "Skid row*" OR "sleepers out"))

#4 AB=(street NEAR/3 (life OR living OR lives OR youth* OR child* OR people OR person*) )

#3 AB=((street* OR private OR improvised OR shelter* OR emergency OR temporar* OR insecure OR overcrowded OR precarious OR stable OR marginal* OR Unstable OR Transitional OR Recovery OR "non permanent") NEAR/3 (dwell* OR hous* OR home* OR accommodat*) )

#2 AB=(evict* OR homeless* OR "housing excl*" OR "residential stability")

#1 AB=(Homeless NEAR/1 (person* OR youth*) )

## 3 Appendix 3: Summary characteristics of intervention studies

| **First author & year**  **Study design** | **Type of CM** | **Research question**    **Funding source** | **Intervention components** | **Location** | **Age** | **Ethnicity** | **Complexity of needs**    **ETHOS status** (FEANTSA 2017) | **Follow-up period(s)** | **Outcomes** |
| --- | --- | --- | --- | --- | --- | --- | --- | --- | --- |

| **At Home/** **Chez Soi** Adair 2016, 2017 Aquin 2017 **Aubry 2015,** 2016, 2019 Chu 2020 Chum 2020  Chung 2018 Durbin 2018 Edalati 2020 **Goering 2014** Hwang 2012 Kerman 2018, 2019, 2020 Kirst 2015, 2020 Kozloff 2016a, 2016b, 2016c  Mejia-Lanchero 2020 O'Campo 2016 Pakzad 2017 Poremski 2016, 2017 Stergiopoulos  2015a,2015b, 2016, 2019  Urbanowski 2018 Veldhuizen 2015  Volk 2016  Whisler 2021 RCT | | HF | To assess the effect of Housing First on homelessness for Canadians with severe mental illness    Funding source: Largely Mental Health Commission of Canada | I: Housing First (varied ICM and/or ACT) (N=1,158) C: Usual care (UC) (N=990) (Goering 2014) | Vancouver, Winnipeg, Toronto, Montreal, and Moncton, Canada Dates: 2009-2013  **Exceptions:**  Toronto only (Hwang 2012, Kirst 2015, Stergiopoulos 2015)   Toronto only - 2014-2017 follow up (Stergiopoulos 2019)  Montreal only (Poremski 2017) | Mean age 39-42 (five cities)  33% female (five cities) (Goering 2014)  33% female (five cities) (Goering 2014) | Varied reporting. 33-41% white  22% aboriginal, 25% other ethnic group (Goering 2014) | Medium and High    ETHOS: Varied 1-6 | 24 months  Exceptions: 3 year extension in Toronto (Mejia-Lanchero 2020, Stergiopoulos 2019) 42 months in Montreal (Poremski 2017) | Housing stability Mental health  Costs and cost-effectiveness |
| --- | --- | --- | --- | --- | --- | --- | --- | --- | --- | --- |
| **At Home/ Chez Soi** **Vancouver at Home** Palepu 2013a, 2013b  Parpouchi 2016, 2018 Patterson 2013, 2014 Rezansoff 2017  Russolillo 2013 Somers 2013a, 2013b, 2015, 2017  RCT | HF | | To explore HR with support services to TAU for homeless adults with mental illness    Funding source: Mental Health Commission of Canada | High needs: 1: Housing First with ACT (N=90) I2: Congregate housing with on-site support [CONG] (N=107) C: UC (N=107)  Moderate needs: I1: Housing First with ICM (N=100) C: UC (N=100) | Vancouver 2009-2012 | Varied but mean age ~ 41.  38 for substance dependence, 44 for no substance dependence  ~27% female  substance dependence(29%) no substance dependence(26%) | Varied reporting. ~ 16% Aboriginal, 56% White, 28% Other | High and medium    ETHOS: Varied 1-6 | 24 months | Housing stability Mental health |
| Baumgartner 2012  Herman 2011  Rosenblum 2002  Tomita 2012, 2014, 2015  RCT | CTI | | To assess the effectiveness of a CTI intervention in reducing homelessness for persons with severe mental illness discharged from inpatient psychiatric facilities    Funding source: National Institute of Mental Health | I: CTI AND Clinical treatment and/or other case-management as required. (N=77)  C: Clinical treatment and/or non-CTI case-management as required (N=73) | New York, USA 2002-2006 Location: NR | Mean 38   29% female | 57% african american (Baumgartner 2013) | High    ETHOS: 4. People living in institutions [7. Healthcare institutions (psychiatric hospital)] | Every six weeks for 18 months (9 month intervention) | Housing stability Psychiatric hospitalisation Capabilities and wellbeing |
| Bender 2018  RCT | SCM | | To pilot test a skills-based intervention designed to improve risk detection among homeless youth    Funding source: No funding received | I: Usual case management with risk detection intervention 'Safety Awareness for Empowerment' (SAFE) (N=56) C: Usual case management (N=41) | "An urban metropolitan city", USA 2012 -2013. | 100% 18-21 yrs of age  37% female | 41.9% white; 32.4% biracial/other; 20.3% black; 5.4% latino | Medium (51.4% Substance use disorder)      ETHOS: Varied 1-6 | 6 months post-baseline | Capabilities and wellbeing |
| Bloom 2001 Lennon 2005 Susser 1997  Valencia 1995, 1996  RCT | CTI | | To examine a strategy to prevent homelessness among individuals with severe mental illness by providing a bridge between institutional and community care    Funding source: National Institute of Mental Health | I: CTI in three (3 month) phases plus UC (N=48) C: UC (N=48) | New York USA 1991-1995 | 40% <35  0% female | 73% African American,22% Latino, 3% biracial, 2% caucasian | High (severe mental health diagnosis, >50% alcohol dependent, other traumas)    ETHOS: 3. People living in accommodation for the homeless [3. Homeless Hostels] | Monthly to 18 months | Housing status Psychiatric emergency room visits Physical health Crime/criminalisation Capabilities and wellbeing |
| Bovell-Ammon 2020  RCT | SCM | | To investigate whether Housing Prescriptons as health care can improve housing stability and effect the health of medically complex families with children    Funding source: Social Interventions Research and  Evaluation Network at the University of California and Boston Foundation’s Health Starts at Home Initiative | I: Housing Prescription (N=36) C: UC (list of housing services, hospital based social work and care navigation services) (N=31) | Boston?, USA  2016-2019 | Mean age of index child = 2.8yrs[SD=2.8]  Gender NR | Parent: 17.9% white; 52.2% black or african american; 13.4% other; 16.4% no response.  Child: 17.9% white; 55.2% black or african american; 11.9% other; 14.9% no response | Medium (member of family having a serious illness/disability)      ETHOS: Varied 1-6 | Every 6 months for 2 years | Housing status Mental health (parents) Employment Access to health services |
| Bradford 2005  RCT | SCM | | To examine the effectiveness of a shelter-based intervention, including intensive outreach, with continuity of care to engage homeless individuals with psychiatric and substance abuse problems    Funding source: NR | I: Intensive outreach (N=51) C: UC in the facility (N=51) | NR, USA Dates NR | Mean age (years) intervention group 39.09; control = 39.68  Intervention - 35% female control - 27% female | Intervention: 35.3% white; 64.7% black Control: 41.2% white; 54.9% black; 3.9% hispanic | High (78.7% substance use disorder; majority of population also anxiety/psychotic/mood disorder)    ETHOS: NR | From entry to exit of the programme? | Physical health Substance use Employment |
| Braucht 1996  RCT | ICM | | To investigate the effectiveness of case management for homeless substance abusers    Funding source: National Institute on Alcohol Abuse and Alcoholism | I: ICM (N=163) C: UC (N=160) | Denver, Colorado, USA 1991-1992 | mean age = 35  15% female | 56% caucasian, 29% african-american; 10% hispanic; 4% native american; 1% other/missing | Medium (alcohol/substance use issues)    ETHOS: NR | Baseline, 4 months and again 6 months post-discharge | Housing status Mental health Substance use Capabilities and wellbeing |
| Brown2016  nRCT | HF | | To investigate if Housing First is an effective intervention for reducing homlessness and service utilisation among vulnerable adults    Funding source: NR | I: Housing First (CM type NR) (N=91) C: UC (N-91) | Possibly Seattle, WA, USA Dates NR | Mean age 43  26% female | 56% White, 24.8% Black, 6.6% Asian/Pacific Islander, 12.7% Latino/Native American/Mixed | High  (High Psychiatric needs)    ETHOS: 4. People living in institutions | one year follow-up | Housing stability Mental health |
| Buchanan 2009   RCT | ICM | | To assess the health impace of a housing and case management programme for homeless people with HIV    Funding source: Michael  Reese Health Trust, Chicago Community Trust, the  AIDS foundation of Chicago, the Fields Foundation,  the Prince Charitable Trusts, the Siragusa Foundation,  and Housing and Urban Development’s Housing  Opportunities for People | I: Permanent housing with ICM (N=54) C: UC (discharge planning usually provided, referrals to shelter, eligible for case management through normal programme) (N=51) | Chicago , USA 2003-2007 | Mean age: intervention = 45; control = 43  Intervention = 28% female; control = 16% female | Intervention (91% African American, 2% Latino, 4% white, 4% other); control (86% african american, 6% latino, 2% white, 6% other). | High (Alcohol and substance abuse disorders, mental disorders, HIV-positive)    ETHOS: Varied 1-6 (‘those who had no stable housing 30 days prior’) | 12 months | Physical health |
| Burt 2012  nRCT | SCM | | To examine the impact of a housing and demonstration project (LA's HOPE) in providing employment for homeless adults with mental illness    Funding source: Community Development  Department of the City of Los Angeles | I: LA HOPE case management (N=56) C: Propensity score matched comparison group from 15 other programme's in the city N=415) | LA, USA 2004-2005 | Intervention: 2% 18-24yrs; 41%25-45; 40% 46-59yrs; 7% 60+  Control:  5% 18-24YRS; 55% 25-45YRS; 35% 46-59yrs; 5% >60yrs  Intervention = 54% female; control = 29% female | Intervention:  57% african american; 30% white; 5% hispanic; 7% other  Control: 40% african american; 35% white; 19% hispanic; 5% other | High **(**All had an Axis I psychiatric disorder and 63% and 52% of intervention and control groups respectively, had a cooccurring substance use disorder)    ETHOS: Varied 1-6 | Data on employment and housing were collected through June 30, 2007, giving each enrollee at least 13 months of follow up | Tenancy in permanent supportive housing (days housed) Housing stability  Employment |
| Caslyn 1998 Trial 2 Kenny 2004 Morse 1997  RCT | ACT | | To compare the effectiveness of the standard ACT team against an ACT team that also included community workers    Funding source: National Institute of Mental Health | I1: ACT  I2: ACT team including community workers (N=105 combined ACT groups) C: Brokered case management with 85:1 staff client ratio (N=60) | St.Louis, USA 1990-1993 | Mean age = 34.8  42% female | 55% African American; 45% Caucasian | NR    ETHOS: 4. People living in institutions (7. Health care institutions) | Three monthly to 24 months | Housing status Psychiatric symptoms Capabilities and wellbeing |
| Calsyn 2005  RCT | ACT | | To test the effects of ACT versus Integrated Treatment versus TAU on homeless people with mental illness and substance abuse disorders    Funding source: National Institute of Mental Health | I1: Integrated treatment (same mental health/substance abuse health professional(s)) I2: ACT C: UC [Numbers NR] | NR, USA 1998-2003 | Mean age = 40   21% female | 74% African-American | High    ETHOS: 1. People living rough; 2. People in emergency accommodation | 6-, 12-, 18- and 24-months | Crime/criminalisation |
| Cheng 2007  Marcus 2012 O'Connell 2008, 2012, 2017 Rosenheck 2003 RCT | ICM | | Assessing the causal effect of housing vouchers for decreasing homelessness in veterans with mental illness    Funding source: Veterans Affairs, New England Mental Illness Research, Education and Clinical Centre | I1: HUD-VASH with ICM and housing vouchers [N=182] I2: Case management only [N=90] C: UC [N=182] [Rosenheck 2003] | San Francisco, San Diego, New Orleans, Cleveland, USA  1992-1998 | Mean age = 42 [O'Connell 2008]  5% female [O'Connell 2008]   Subgroup: 0% female and all with substance abuse problems [O'Connell 2012 | Ethnicity = 64% african-american.    Subgroup: 66% [O'Connell 2012] | High (Past drug use, psychiatric condition, veterans)    ETHOS: 1. People living rough; 2. Overnight shelters; 3. Homeless accommodation (shelters and temp) | 6, 12, 18, 24, 30, and 36 months  [5 years O'Connell 2008] | Days Housed in past 90 days  Psychiatric distress Substance use Physical health Capabilities and wellbeing Costs and cost-effectiveness |
| Cherner 2017  nRCT | HF | | To explore outcomes for homeless adults with problematic substance abuse receiving a Housing First program    Funding source: Canadian Mental Health Association | I: Housing First (ICM) (N=89) C: UC (N=89) | Ottawa, Canada Dates NR | Mean age = 40  55% female for HF, 40% for control | NR | High    ETHOS: Various 1-6 | every 6 months, total of 2 years | Housing status Substance use Capabilities and wellbeing |
| Clark2016  nRCT | HF | | To examine two evidence-based models of case management for people with co-occuring disorders and histories of chronic homelessness    Funding source: Substance Abuse and Mental Health Services Administration | I1: CTI (N=144) I2: ACT (N=90) | Florida?, USA Dates NR | Mean age CTI - 46 ACT - 41  CTI - 12% female ACT - 31% female | 55.13% Caucasian, 36.33% African American, 13.67% Hispanic | Medium      ETHOS: Various 1-6 | 6 month follow-up | Housing status Substance use |
| Collins 2020  RCT | CTI | | To examine process findings from *Partnering for Success*, to address the needs of child welfare-involved families    Funding source: Reinvestment Fund, George Gund Foundation, Cleveland Foundation, Nonprofit Finance Fund, Sisters of Charity Foundation of Cleveland | I: CTI with ICM and trauma-informed approach plus traditional child welfare services (N=90)  C: UC (traditional child welfare services) (N=73) | Cuyahoga County, Ohio, USA 2015-2017 | Age 31.8±8.8  93% female | 71% black; 21% white; 8% hispanic | Medium    ETHOS: Various including 3. Accommodation for the homeless, 5. Non-conventional housing & 6. Temporarily with family and friends | 12 months 24 months | Need to access emergency shelter, rapid re-housing or coordinated assessment)  Capabilities and wellbeing |
| Conrad 1998  RCT | SCM | | To explore the effectivenes of case-managed residential care (CMRC) in reducing substance abuse, increasing employment, decreasing homelessness and improving health in homeless addicted veterans after inpatient discharge    Funding source: Health Services Research & Development Service;  National Institute on Alcohol Abuse and Alcoholism | I: ≤6 months transitional residential care and ongoing CM to 12 months (N=178) C: 21d hospital programme with referral to community services (N=180) | Illinois, USA Dates NR | Mean age ca 40 (range 25-70)  0% female | Ca 75% African American; 25% White | High (66% with 2+ substance misuse issues; Veterans; psych hospitalisation)    ETHOS:  1. Living rough; 2. In emergency accommodation; 6. Temporarily with friends/family | 5 years 3,6,9(during treatment),12,18,24 months (post treatment) | Housing status Addiction severity Substance use Capabilities and wellbeing |
| Cox 1993, 1998  RCT | ICM | | To test whether an ICM intervention would be effective with a group of homeless chronic public inebriate clients    Funding source: National Institute on Alcohol Abuse and Alcoholism | I: ICM (N=150) C: UC (N=148) | Seattle, USA Dates NR | mean age = 42.9 (+-10yrs) years  19% female | 44% white, 16% black, 33% native american, 7% hispanic | Medium (substance abuse/ alcohol use)    ETHOS: Various 1-6 | 6, 12, 18, 24 months (although 24 months truncated due to funding) | Housing status Employment |
| de Vet 2017  RCT | CTI | | Women only: To examine the effectiveness of CTI for abused women transitioning from women’s shelters to community living    Funding source: Netherlands Organization for Health Research and Development: Academic Collaborative Center for Shelter and Recovery | I: CTI in three (3 month) phases (N=94) C: UC (N=89) | 18 shelters Netherlands 2010-2014 | ~ 41 years  46% female | 67% Dutch native, circa 33% migrant | NR    ETHOS: 3. People living in accommodation for the homeless [3. Homeless Hostels] | 3,6,9 months | Housing status Psychological distress Capabilities and wellbeing |
| Ellison 2020  RCT | ICM | | To test the impacts of peer specialists on housing stability, substance abuse, and mental health status for previously homeless veterans with mental health and substance abuse issues    Funding source: NR | I1: HUD-VASH case management (N=85)  I2: UC case management (N=81) | Two VA medical centres, USA 2012-2016 | Mean age = 52.77 +-9.04  7% female | minority ethnicity = 46.99% | High (substance abuse or psychiatric diagnosis, veterans)    ETHOS: Various (likely 1-5) | Baseline, midpoint (about 4–6 mo), and endpoint (about 9–12 mo). Some interviews conducted up to 2.5 years after initial entry | Housing stability Mental health Substance use |
| Erdem 2014  RCT | SCM | | To explore treatment outcomes of an integrative housing intervention on capabilities for substance-abusing homeless mothers    Funding source: NR | I: Ecologically Based Treatment Intervention (EBT) - financial/utility assistance, substance abuse counselling, case management [N=30] C: UC with case worker and subsidised housing [N=30] | Midwestern City, USA 2010-2011 | Average 26.3 (SD 6.01) Range 18-41  100% female | 75% African American | Medium (substance or alcohol use)    ETHOS: 3. People living in accommodation for the homeless | 3,6,9 months | Housing status Mental health Employment Capabilities and wellbeing |
| Essock 2006 Frisman 2009  RCT (secondary analysis) | ACT | | To compare two different models of community-based case management (ACT and SCM) on clients with co-occurring mental health and substance abuse disorders    Funding source: National Institute of Mental Health, National Institute on Alcohol Abuse and Alcoholism, and the Substance Abuse and Mental Health Services Administration | I1: ACT (N=99) I2: Standard clinical case management (N=99) | Connecticut, USA 1993 - 2001 | mean age = 38±.8 yrs  28% female | 55% african-american; 14% hispanics; 4% other | High (100% psychiatric diagnosis, substance use disorder)    ETHOS: Various (likely 1-6) | every 6 months for 3 years | Mental health Substance use |
| Ferreiro2020  RCT | HF | | To assess feelings of loneliness among participants of an HF programme    Funding source: NR | I: Housing First (ACT) (N=46) C: UC (N=41) | Barcelona, Spain Dates NR | Mean age = 50  HF 15% female TAU 17% female | HF 73.9% Europe, 17.4% Africa, 4.3% South America, 4.3% Asia TAU 78% Europe, 14.6% Africa, 4.8% South America, 2.4% Asia | Medium    ETHOS: 1. Living rough; 2. In emergency accommodation; 3. Accommodation for the homeless | baseline, at 8mo, and at 21 months | Housing status Mental health Substance use |
| Grace 2014  RCT | ICM | | To explore joined up services for young people experiencing homelessness and unemployment    Funding source: State of Victoria’s Community Support  Fund | I: ICM with joined up delivery (N=222) C: UC (N=174) | Melbourne, Cheltenham, Bendigo, and Frankston, Australia 2005 -2008 | 73% 18-24 year olds; 27% 25-35 yr olds  35% female | 5% aboriginal or Torres Strait Islander | NR    ETHOS: Various 1-6 | 12 and 24 months | Housing stability Employment and income |
| Graham-Jones 2016  nRCT | SCM | | To test the effectiveness of a health advocate's casework with homeless people in terms of health related quality of life    Funding source: NHS Management Executive | I1: Health advocacy [N=22] I2: Health advocacy with outreach registration [N=53] C: UC [N=42] | Liverpool 8, UK 1993-1995 | 74% < 30 years old  72% female | 91% white British | Medium? (Homelessness due to domestic violence in 44%: 45% long term illness)    ETHOS: 3. Living in accommodation for the homeless (3. homeless hostels/); 4. Temporary accommodation) | 3 years months | Housing status Capabilities and wellbeing |
| Gulcur 2003  Tsemberis 2004  RCT      Greenwood 2005 (subset) | HF | | To compare two approaches to housing chronically homeless individuals with psychiatric difficulties and often substance abuse    Funding source: Center for Mental Health Services’ Substance Abuse and  Mental Health Services Administration | I: Housing First (ACT) (N=99) C:UC (Continuum of Care model; conditional) (N=126)    Greenwood 2005 subset:  I: Housing First (ACT) (N=93) C: UC (Continuum of Care model) (N=104) | New York State, USA Dates NR | Mean age = 42  23% female  Greenwood 2005 subset:  Mean age = 42  24% female | 28% caucasian, 40% african-american, 14.7% hispanic, 17.3% mixed/other    Greenwood 2005 subset:  29.4% caucasian, 38.1% african-american, 13.7% hispanic, 18.8% mixed/other | Medium (most with substance abuse)    ETHOS: Various 1-4, 6 | every 6 months for 2 years      Greenwood 2005 subset:  To 3 years | Housing status Time in psychiatric hospitals Substance use |
| Hall 2018   nRCT | SCM | | To compare housing retention and use of crisis public services (jail, emergency department visits, hospitalization, and substance detoxification) between individuals treated and untreated for SUD before move-in to a low-demand supportive housing program in New York City    Funding source: NR | I: Housing services plus substance use treatment [N=1425] C: Housing services alone [N=512] | New York, USA 2007- 2014 | I: 15% over 50 C: 28% over 50  Intervention: 18% 18-34 yrs; 24% 35-44yrs; 42% 45-54yrs; 15% 55+  Control:  15% 18-34yrs; 19% 35-44yrs; 38% 45-54yrs; 28% 55+ yrs  I: 25% female C: 14% female | I: 57% non-hispanic Black C: 66% non-hispanic Black  Intervention: 32% hispanic, 57% non-hispanic black, 10% non-hispanic white, 2% other race   Control:  21% hispanic, 66% non-hispanic black, 11% non-hispanic white, 3% other | High (mental and physical disorders)      ETHOS: Various 1-3,5 | 2 years | Tenure in supportive housing  Substance use Access to health and social services |
| Hanratty 2011  nRCT | HF | | To investigate HF for long-term homeless individuals with work-limiting disabilities residing in pubic shelters    Funding source: NR | I: Housing First (CM type NR but 'team') (N=264) C: UC (N=264) | Hennepin County, Minnesota, US Dates NR | Mean age = 46  I: 25% female C: 14% female | NR | Medium    ETHOS: NR | NR | Shelter use Crime/criminalistion |
| Hurlburt 1996a, 1996b  RCT | ICM | | To evaluate the effectiveness of providing housing vouchers as a means of providing independent housing to the severely mentally ill homeless    Funding source: National Institute of Mental Health and Center for Mental Health Services | I1: Traditional case management with Section 8 housing vouchers I2: Comprehensive case management without section 8 housing vouchers  I3: Traditional case management without access to section 8 housing vouchers [Numbers in each group NR] | San Diego, California, USA.  Dates NR | Split into age categories:  18-29yrs = 24.9%; 30-39yrs = 41.7%; 40-49yrs = 24%; 50+yrs = 9.4%  33% female | 63% white; 19.6% black; 12.4% hispanic; 5% other | Medium (mental illness)    ETHOS: Various 1-6 | Across a 2 year period (monthly data collection) | Housing status Housing consistency |
| Kidd 2020  RCT | CTI | | The study was designed to generate feasibility information related to trialing HOP-C for youth transitioning out of homelessness to housing    Funding source: NR | I: HOP-C (6 months mental health and peer support plus CM) [N=34] C: Case management alone plus UC [N=31] | Urban area, Canada 2017-2018 | 22  40% female | 31% black, 22% white | Medium (youth)    ETHOS: Likely 3. Living in accommodation for the homeless.  ‘Experience of homelessness but some current stability’ | 6 months | Housing status Mental health Capabilities and wellbeing |
| Koffarnus 2011  RCT | SCM | | To assess the efficacy of the Therapeutic Workplace, a substance abused intervention that promotes abstinence, among homeless alcoholics    Funding source: National Institutes of Health | I1: Alcohol abstinence req. for paid employment training; N=43] I2: Payment for employment training [N=42] C: Unpaid employment training [N=39] | Baltimore, MD, USA 2001-2005 | Mean age ca 43   ca 20% female | Large difference I1 63% white, I2 and C ca 43% whilte  Intervention: 43.6% white; 56.4% black Paid control: 42.9% white; 50% black; 7.1% other  Contingent paid control: 62.8% white; 37.2% black | Medium    ETHOS: Various 1-6 | 26 weeks | Substance use |
| Korr 1996  RCT | ACT | | To present housing outcomes from an Assertive Case Management programme for the homeless mentally ill admitted to psychiatric hospitals    Funding source: Illinois Department of Mental Health and Developmental Disabilities | I: Bridge Services [N=48] C: UC - available community services [N=47] | Chicago, USA Dates NR Location: on streets and in clients' accommodation | I: mean age 37 C: mean age 40  I: 13% female C: 28% female | I: 48% white, 43% black C: 50% white, 43% black | High (major mental illness, hospital incarceration)    ETHOS: Various 1-6 but at study entry 4. In institutions (7. Health institutions) | 6, 12 months | Housing status Days in hospital |
| Lako 2018  RCT | CTI | | Women only: To examine the effectiveness of CTI for abused women transitioning from women’s shelters to community living    Funding source: Netherlands Organization for Health Research and Development | I: CTI in three (3 month) phases (N=70) C: UC (N=66) | 9 shelters Netherlands 2010-2014 | Mean age 34 (SD 8)   100% female | ~ 27% Dutch native 61% 72% migrant | NR    ETHOS: 3. People living in accommodation for the homeless [3. Homeless Hostels] | 3,6,9 months | Post-traumatic stress Capabilities and wellbeing |
| Lapham 1993, 1996  RCT | HF [ACT vs ICM vs TAU]] | | Evaluation of a high and medium-intensity programme for homeless alcohol abusers    Funding source: National Institutes of Health | I1: High intensity (CM plus peer-supervised housing (N=161) I2: Medium intensity  (peer supervised housing only) (N=164) C1: Housing UC (N=92) C2: Non-housed UC (N=52) | Albuquerque, USA 1990 - 1993 | Median age = 37 years   12% female | 41% non-hispanic white, 31% hispanic, 18% native american and 10% other | High    ETHOS: Various 1-6 | 10 months | Housing status Substance use Employment |
| Lehman 1995, 1997, 1999  RCT | ACT | | To evaluate the effectiveness of ACT for homeless persons with severe and persistent mental illness    Funding source: Center for Mental Health Services | I: (N=77) ACT C: (N=75) UC | Baltimore, USA 1991-1993 | Mean age ca 39   11% female | ca 73% African American (I 61% C 84%); ca 24% white (I 35% C 12%) | High    ETHOS: 1. Living rough, 2. Emergency accommodation, 3. Accommodation for the homeless, 4. People in Institutions | 2, 6, 12 months | Housing status Hospital visits Physical health Capabilities and wellbeing  Costs and cost-effectiveness |
| Levitt 2013  RCT | ICM | | To compare Home to Stay, a pilot of intensive housing placement and community transition services for homeless families    Funding source: Institute on Alcohol Abuse and Alcoholism | I: Home to Stay (N=138) C: Standard services (shelter, caseworkers meet bi-weekly with caseworkers and after permanent housing obtained - shelter services cease) (N=210) | Location: New York City 2010-2012 | control group mean age 34=yrs +-7 intervention group 34 yrs +-9  Gender NR | NR | NR    ETHOS: NR (‘episodic and recidivist’) | Follow up period was from entry to programme to exit of the programme | Shelter status |
| Malte 2017 Cox 2017  RCT | ICM | | To determine whether homeless veterans receiving intensive addiction/housing CM has improved outcomes compared to a housing support group control    Funding source: VA Health Services Research &  Development | I: ICM (N=91) C: Drop-in housing support group (weekly) (N=90) | VA Puget Sound, Seattle Division, USA 2011-2015 | mean age = 51 years  2% female | Intervention:  60.4% white; 35.2% black;4.4% other Control: 58.9% white; 31.1% black; 10% other | High (homelessness, substance and alcohol use, psychatric needs, veterans)    ETHOS: Various 1-6 | 12-24 months at 3 or 6 monthly intervals. | Housing status Mental health Substance use Access to health and social care Physical health |
| Mares 2011  nRCT | ACT | | To explore the effect on service use and treatment outcomes amongst chronically homelessness clients receiving comprehensive housing and healthcare services via the Collaborative Initiative on Chronic Homelessness (CICH) programme versus usual care    Funding source: US Department of Housing and Urban Development, HHS.gov and US Department of Veterans' Affairs | I: CICH program (N=281) C: UC (N=104) | Multiple sites, USA 2004-2008 | Mean age ca 46  ca 22% female | Ca 60% 'racial/ethnic minority'  intervention = 58% racial/ethnic minority; control = 63% racial/ethnic minority | High (>50% each for mental health, substance abuse, phys health problem)    ETHOS: NR (‘chronically homeless’) | 2 years | Housing status Mental health Substance use Access to health and social care Capabilities and wellbeing |
| Marshall 1995  RCT | SCM | | To evaluate a social services case-management team for people with long-term mental disorders    Funding source: Medical Research Council and  Wellcome Trust | I: Case management (N=40) C: UC (N=40) | Oxford, UK 1991 | Mean age 53 years  15% female | NR | Medium  (mental health)    ETHOS: Various 1-6 | 7, 14 months | Housing quality Mental health Crime/criminalisation Employment and income |
| McHugo 2004  RCT | ACT | | To compare the provision of integrated provision of case management and housing services versus parallel provision for adults with severe mental illness    Funding source: Substance Abuse and Mental Health Services Administration | I: Parallel ACT (community health agencies) and housing provision (realtors/landlords) (N=60) C: Integrated mental health and housing provision via ICM (N=61] | Washington DC, USA Dates NR | Mean age 40 | 83% African American | High    ETHOS: Various but including 2. Emergency accommodation, 6. Conventional housing | 6, 12, 18 months | Housing status Mental health Capabilities and wellbeing |
| Montgomery 2013  nRCT | HF | | To compare an HF (HUD-VASH) with a treatment first programme for homeless veterans with severe mental illness    Funding source: NR | I: Housing First (CM type NR) [N=107] C: UC (Continuum of Care model) [N=70] | USA Dates NR | Mean age 56 in HF, 49 in TAU  HF 94% women TAU 75% women | HF 0.9% hispanic, 93.5% black/african american, 5.6% white; TAU 7.1% hispanic, 80% black/african american, 8.6% white, 4.3% other | Medium    ETHOS: NR | NR | Housing status Inpatient visits for mental health |
| Morse 1992  Caslyn 1998 Trial 1  RCT | ACT | | To compare the effectiveness of three community-based treatment programs serving homeless mentally ill people    Funding source: National Institute of Mental Health | I1: ACT (PACT programme) [N=52] I2: Drop in centres with social workers (40:1 client to staff) [N=62] C: Outpatient mental health clinic [N=64] | St. Louis, Missouri, USA 1988-1989 | Mean age = 34yrs  42% female | 52.5% non-white (almost all of these African-American) | Medium (71.9% psychiatric disorder some substance abuse)    ETHOS: 2. Emergency accommodation | Interviewed monthly but main was a 12 month follow-up. | Housing status Mental health Substance use Income Capabilities and wellbeing |
| Morse 1997  Kenny 2004  RCT | ACT | | To compare three types of CM in helping people with severe mental illness who were homeless or at risk of homelessness    Funding source: NR | I1: Broker case management I2: ACT I3: ACT with community workers [Numbers NR] | St.  Louis, Missouri, USA 1990-1993 | mean age = 35 +-10  42% female | 45% caucasian; 55% african-american | High    ETHOS: Various 1-6 | 6,12,18 months | Housing status Mental health Substance use Income |
| Morse 2006 RCT  Linked to Morse 2008 nRCT | ACT | | To compare the effectiveness of four intervention in providing services to homeless clients with dual disorders    Funding source: NR | I1: Integrated Assertive Community Treatment (IACT) (N=61)  I2: Assertive Community Treatment only (ACTO) (N=65) I3: Standard case management (N=65)  Morse 2008 also included: I4:New Integrated Assertive Community Treatment (NIACT) (N=79) | NR, USA Dates NR Location: Community provision | mean age = 40 yrs (Range 18 - 66yrs)  20% female (Morse 2006)  24% female (Morse 2008) | 73% african-american; 2% other minorities; 25% caucasian | High (psychiatric disorder, substance use disorders)    ETHOS: 1. Living rough, 2. Emergency accommodation; 5. non-conventional housing | 24 months (Morse 2006)  18 months (Morse 2008) | Housing status Mental health Substance use  Costs and cost-effectiveness |
| Nyamathi 2001  nRCT | SCM | | To examine the 6-month impact of three cognitive-behavioural HIV risk-reduction programmes on women residing in emergency or sober-living shelters and their intimate sexual partners    Funding source: National Institute of Mental Health | I1: PEER MENTOR (N=258) I2: nurse case-management (N=360) C: UC (N=330) | Los Angeles, USA (35 shelters) 1995-1998 | Mean - mid 30s  100% female but plus sexual partner | Wide variation across groups  Peer intervention: Women - 41.4% african-american, 46.5% hispanic/latino, 10.1% angloamerican, 2% other Partners - 47% african-american, 46% hispanic/latino, 7% angloamerican  Nurse case managed: women - 65.8% african-american, 21.9% latino/hispanic, 11.4% angloamerican, 0.9% other. Partners - 69.8% african-american, 21.7% hispanic/latino, 8.5% angloamerican Standard care: women - 80.2% african-american, 10.8% hispanic/latino, 7.2%angloamerican, 1.8% other. Partners - 82.2% african-american, 13.9% hispanic/latino, 3% angloamerican, 1% other | Medium (substance use)    ETHOS: 2. Emergency accommodation;  3. Accommodation for the homeless;  6. Temporarily in conventional housing | 6 months | Capabilities and wellbeing |
| Nyamathi 2008  nRCT | SCM | | To determine whether a validated nurse case-managed intervention with incentives and tracking would improve adherence to latent tuberculosis infection in homeless persons with characteristics predictive of non-adherence    Funding source: National Institutes of Health | I: Nurse case-managed Isoniazid treatment and incentive ($5 per treatment) [N=279] C: UC (single education session) [N=241] | Los Angeles, USA 1998-2003 | Mean 42 years  20% female | 81% Black | High  (veterans, injection drug use, daily alcohol/drug use, poor physical health)  ETHOS: 2. Emergency accommodation (80% emergency shelter, 20% drug recovery shelter) | 6 months | Physical health |
| Nyamathi 2009a Nyamathi 2009b  nRCT | SCM | | To evaluate the effectiveness of a nurse case-managed intervention compared with that of two standard programmes on completion of combined Hepatitis A virus and HBV vaccine among homeless adults    Funding source: National Institute on Drug Abuse | I1: Nurse CM + hepatitis education + incentives + tracking [NCMIT N=332] I2: Hepatitis education + incentives + tracking [SIT N=281] C: Hepatitis education + incentives [SI N=252] | Los Angeles, USA (12 shelters) 2003-2007 | Mean 42 years  23% female | 69% African American | Medium (substance use)    ETHOS: 1. Living rough;  2. Emergency accommodation;  4. Health care institutions (drug treatment) | 6 months | Access to health and social care (vaccination status) |
| Nyamathi 2015 Nyamathi 2016  RCT | ICM | | To explore three levels of peer coaching and nurse-delivered interventions on completion of Hepatitis A and B vaccination in homeless men on parole    Funding source: National Institute on Drug Abuse | I1: Intensive peer coaching and nurse case management (PC-NCM) (N=114) I2: Intensive peer coaching with minimal nurse involvement (PC) (N=117) C: UC (N=114) | Los Angeles, USA 2010-2013 | Mean 40 years  0% female | 46.3% african-american; 32.5% latino; 15% white; 6.2% other | High  (post incarceration and other health issues)    ETHOS: Various 1-6 (‘one who does not have a fixed, regular, and adequate nighttime residence’) | 6, 12 months | Housing status Substance use Physical health Access to health and social care Crime/criminalisation Employment and income |
| Nyamathi 2017  RCT | SCM | | To compare the effect of dialectical behavioral therapy-corrections modified (DBT-CM) program with a health promotion (HP) programe on achieving drug and alcohol abstinence among female homeless parolees/probationers    Funding source: National Institutes of Health | I: Dialectical behavioral therapy-corrections modified case management (DBT-CM) program [N=65] C: Health promotion (HP) education [N=65]; also once/week looking at chronic diseases | Los Angeles and Pomona, CA, USA 2015-2016 | Mean age ca 39   100% female | ca 40% Black, 40% Latino, 13% White  Control = 16/9% white; 36.9% black; 40% latino; 6.2% other Intervention = 10.8% white; 44.6% black; 40% latino; 4.6% other | High (incarceration plus majority drug use)    ETHOS:  Various 'Considered homeless prior to discharge from incarceration' | 6 months (12 week intervention) | Substance use |
| Padgett2011   nRCT | HF | | To compare the Housing First and Treatment First approaches for homeless adults with serious mental illness    Funding source: National Institute of Mental Health | I1: Housing First (CM type NR) [N=27] I2: Treatment First [N=48] | New York City, USA Dates NR | Mean age 44 for HF, 40 for TF  27% female for HF, 28% female for TF | 22% white, 33% african-american, 30% latino, 8% asian and 8% others for HF; 13% white, 62% african-american, 19% latino, 6% asian and 4% others for TF | High (mental health and substance abuse)    ETHOS: NR | 6, 12 months | Substance use |
| Rosenblum 2002  nRCT | ICM | | To contact an assessment of a mobile outreach clinic with ICM for homeless substance users    Funding source: National Institute on Drug Abuse | I: ICM C: Self-referral to social worker, no incentives for repeated sessions [Numbers NR] | New York City, USA 1997 Locations: Various, from a van in-person | Age NR  Gender NR | NR | High (substance use, psychiatric disorders and physical health issues)    ETHOS: NR | Reported a 4 month follow-up (N=128) | Housing status Substance use Physical health Access to health and social care |
| Sadowski 2009  RCT | HF (with CTI elements) | | To assess the effectiveness of a case management and housing program in reducing use of urgent medical services among homeless adults with chronic medical illnesses    Funding source: Michael  Reese Health Trust, the Chicago Community Trust, and  the AIDS Foundation of Chicago | I: Transitional housing followed by long-term housing with on-site case management (CM type NR) [N=201] C: UC - Standard discharge planning from social workers [N=206] | Chicago, USA 2003-2007 Location: Respite care or accommodation | Mean age intervention = 47yrs(SD=8) Mean age control = 46yrs(SD = 9)  26% female intervention; 21% female control | 78% African American  intervention: 81% african american; 8% hispanic; 7% white; 4% mixed/other Control: 76% african american; 8% hispanic; 10% white; 6% mixed/other | High (hospitalisation for chronic illness, > 50% with substance misuse)  ETHOS: Various 1-6 | 1, 3, 6, 9, 12, 18 months | Hospitalisation and emergency department visits Access to health and social care Capabilities and wellbeing |
| Samuels 2015  RCT | CTI | | To test the effect of a time-limited management model targeting homeless mothers who are experiencing mental health problems    Funding source: US Dept, of Health and Human Services, Public Health Service, Substance Abuse and Mental Health Services Administration, Center for Mental Health Services, Center for Substance Abuse Treatment | I: CTI in three (3 month) phases (N=97) C: UC including permanent housing (N=113) | New York, USA 2001 - 2005 | Mean age 33 (SD 8)   100% female (mothers) | 56% African American, 15% Caucasian, 13% Hispanic/Latino | Medium (mental health and/or substance abuse)    ETHOS: 3. People living in accommodation for the homeless [3. Homeless Hostels] | 3, 9, 15 months | Mental health (mothers) |
| Shaw 2017  RCT | CTI | | To establish whether or not CTI is effective in (1) improving engagement of discharged male prisoners who have mental illness with community mental health teams (CMHTs) and (2) providing practical support with housing, finance and re-establishing social networks    Funding source: National Institute for Health Research | I: CTI (N=72) C: UC (N=78) | 8 English prisons, UK Dates NR | Mean age NR  0% female | NR | High (severe mental health diagnosis, incarceration)    ETHOS: 4. People living in institutions (8. Penal institutions) | 6 weeks (end intervention), 6 months, 12 months | Mental health service contact Costs/cost effectiveness |
| Shern 2000   RCT | ICM | | To test a psychiatric rehabilitation approach for street-dwelling persons with severe mental illness    Funding source: NR | I: Choice programme (N=91) C: UC (N=77) | New York City, USA 1991-1994  Location: Community | Mean age 40  24% female | 61% Black | High Major mental illness diagnosis (91%) Lifetime alcohol or substance abuse disorder diagnosis (54%)    ETHOS: 1. People living rough; 5. Non-conventional dwellings ('staying somewhere not meant for overnight residence') | 24 months follow up (at 6 month intervals) | Housing status Unmet housing needs Mental health Access to health and social care Capabilities and wellbeing |
| Shinn 2015  RCT | CTI | | To compare the effects of a Family CTI intervention with usual care for children in 200 newly homless families in which mothers had diagnosable mental illness or substance problems    Funding source: Substance Abuse and Mental Health Services Administration, National Institute of Mental Health | I: CTI in three (3 month) phases (N=97) C: UC (N=103) | Westchester County, New York, USA 2001 - 2005 | Mean age 31 (SD 8.) for mothers  311 children—99 ages 1.5–5 years, 113 ages 6–10 years, and 99 ages 11–16 years  100% female (mothers) | Mothers: 65% African American, 25% white, 12% refused, 10% American Indian/Alaskan Native | Medium (Mothers with diagnosed mental health or substance abuse problem)    ETHOS: 3. People living in accommodation for the homeless [3. Homeless Hostels] | 24 months | Mental health (children and adolescents) Capabilities and wellbeing (reported school troubles) |
| Slesnick 2013  RCT | ICM | | To test the effectiveness of an integrative treatment targeting homeless substance abusing mothers with young children in their care    Funding source: NR | I: Ecologically-based ICM (N=30) C: UC (includes emergency shelter for women and their children up to three weeks at the shelter and linkage to housing and support services in the community) (N=30) | Ohio USA 2010 - 2011 | Mean age = 26  100% female | 75% African-American; 11.6% white non-hispanic; 1.7% asian/asian-american; 1.7% hispanic; 10% other/mixed. | Medium (Substance abuse)    ETHOS: Various 1-3, 5-6 | 3,6,9 month follow-up. | Housing status Mental health Substance use |
| Slesnick 2015 Zhang 2018  RCT | SCM | | To compare three interventions, CRA, MET and standard case management on substance use disorders in homeless youth    Funding source: National Institute on Drug Abuse | I1: Community reinforcement approach (CRA) - client matched behavioural change [N=93] I2: Motivational Enhancement Therapy (MET) - client led responsibility for change [N=86] I3: Standard case management [N=91] | Ohio, USA 2006-2010 Location: Drop in centres | Mean age 19  47% female | 66% african-american; 20% white  Overall = 65.56% african-american; 19.6% white not hispanic; 2.22% hispanic; 0.74% native american; 0.37% asian american; 11.48% other | High (Substance use/Youth)    ETHOS: Various 1-6 | 3, 6, 12 months | Housing status Mental health Substance use Capabilities and wellbeing |
| Tinland 2020 Lemoine 2019  RCT | HF | | To deternine whether the Housing First programme for people who are homeless with severe mental health disorders (*Un Chez Soi d'Abord)* improves hospital and emergency department use    Funding source: Programme Hospitalier de Recherche Clinique National, the French Ministry of Health (Direction  Générale de la Santé), the Fondation de France and Janssen Pharmaceutical  Company | I: Housing First (ACT) (N=353) C: UC (N=350) | Lille, Marseille, Paris, Toulouse, France 2011-2018 Location: Home or in City | Mean age 39  18% female | N/A | High (severe mental illness plus disability/past hospitalisations.alcohol or substance abuse)    ETHOS: 1. Living rough; 2. Emergency accommodation; 3. Accommodation for homeless; 4. Institutions. | 6,12,18, 24 months | Housing status Mental health (including emergency department and hosptial admissions) Substance use Capabilities and wellbeing Costs/cost effectiveness |
| Tonks2009 Larimer 2009  nRCT | HF | | To evaluate an HF intervention for chronically homeless individuals with severe alcohol problems with health care use and costs    Funding source: The Substance Abuse Policy Research Program (SAPRP) of the Robert Wood Johnson Foundation | I: Housing First (CM type NR) (N=95) C: Wait list control (N=39) | Seattle, USA  2005-2007 | Mean age 48  6% female | 39% white, 28% african-american | Medium (high alcohol use)    ETHOS: NR | 6 months | Substance use Costs/cost effectiveness |
| Toro 1997   RCT | ICM | | To evaluate an Intensive Case Management intervention for homeless persons    Funding source: U.S. Department of Labor | I: ICM (N=101) C: UC (N=101) | Buffalo metropolitan area, New York state, USA 1990? | Mean age 32   42% female | 54% african-american; 34% white; 11% other | Low (25% veterans)    ETHOS: NR | 6, 12, 18 months | Housing status Mental health Substance use Employment and income Capabilities and wellbeing |
| Towe 2019   RCT | SCM | | To determine whether, for homeless persons living with HIV/AIDS rapid re-housing can improve housing and HIV viral suppression more than standard housing assistance    Funding source: New York City Department of Health and Mental  Hygiene | I: Rapid re-housing with CM (N=119) C: UC (less contact, only 3 months help and had to travel to services) (N=117) | New York City , USA 2012-2014 | Mean age 46  22% female | 60% non-hispanic black; 33% hispanic; 6% non-hispanic white | High (58% mental health diagnosis, 81% alcohol or drug use)    ETHOS: 2. Emergency shelters; 3. Homeless shelters | Baseline, 6 and 12 months | Housing status Physical health |
| Upshur 2015  RCT | ICM | | To evaluate a chronic care model for disease management for alcohol use problems in homeless women    Funding source: National Institute of Alcohol Abuse and Alcoholism | I: Brief intervention and ICM for six months [N=40] C: UC [N=42] | Homeless clinic, USA Dates NR | Mean age I: 45 C: 46  100% female | White I = (43.0%) U = (22.5%)  Black I = (33.3%) U = (50.0%) Other I (23%) U = 27.5%) | Medium    ETHOS: Various 1-6 | Baseline, 3 months, 6 months | Housing status Mental health Substance use |
| Weinreb 2016  nRCT | SCM | | To explore a collaborative care model for homeless mothers with depression    Funding source: National Institute of  Mental Health | I: Collaborative care model (N=42) C: UC (N=25) | New York, USA 2010-2012 | Mean age I: 35 C: 38  100% female | White = I group n=8 C group n=6 Black I group n=20 C group n=13 other I group n=12C group n=5 non specified I group n=2 C group n=1 | Medium    ETHOS: 2. Emergency shelter | 3 months 6 months | Housing status Mental health Employment |

## 4 Appendix 4:  Summary characteristics of implementation studies

| **First author & year** | **Study design**    **Funding source** | **Type of CM intervention** | **Research question(s)** | **Setting** | **Participants**    **ETHOS** (FEANTSA 2017) | **Themes of relevance to case management** |
| --- | --- | --- | --- | --- | --- | --- |
| Adame 2020 | Qualitative: Semi-structured interviews Focus groups    Funding source: Premera Blue Cross | Housing First | To interview residents about their experiences of community and collect their suggestions for improving community building efforts  To understand how residents living in buildings operated by Plymouth Housing defined and experienced community in their day-to-day lives. | Seattle, USA 8 Housing First buildings November 2018 - February 2019 | N=38 Average age 56 34% female 47% caucasian; 26% black/african american Individual interviews (n = 14) Focus groups (n = 24)    ETHOS: 3. Accommodation for the homeless | Importance of: - community - mutual support and care (including CM as listening ear/part of community) - interpersonal connections - gatherings and events |
| Austin 2014 | Qualitative: Semi-structured interviews    Funding source: Department of Veterans Affairs | Housing First | To explore how individuals throughout the organisations experienced and responded to the challenges of transitioning to a Housing First approach | Northeast & Mid Atlantic, South, Midwest and West regions, USA 8 Veterans' Affairs facilities Feb-Dec 2012 | N=95 32 Director/Chief; 17 manager/coordinator; 14 case workers; 32 other staff    ETHOS: 3. Accommodation for the homeless | - Difficulty of Finding suitable accommodation (with no sobriety or treatment participation expectations) in difficult rental market - Facility leadership support (needed by CMs) - Risk of CM overload |
| Blosnich 2020 | Qualitative: Semi-structured interviews    Funding source: Veterans Affairs (VA),  VA Health Services | Housing First | To explore transgender and cisgender veterans' experiences utilising permanent supportive housing through the HUD-VASH programme | Pan USA All HUD-VASH housing February-May 2018 | N=48 14 cisgender women, 17 cisgender men, 17 transgender individuals 29% female Average age 55 50% white, 42% black/african american; Latin/hispanic 6%  75% identified as heterosexual 10% receiving HUD-VASH services for <1 year, 21% >4 years    ETHOS: 3. Accommodation for the homeless | - CM support as key facilitator - additional training needed for CM - multiple changes of CM as barrier to getting HUD-VASH - Lack of understanding of HUD-VASH admission process and wait times as barriers -Clients unable to receive services until homeless -Landlords' reluctance to rent to veterans with substance abuse -For transgender women, sex specific shelter experiences and incongruence of identification cards and gender expression |
| Chinman 2000 | Quantitative: Correlation study  (longitudinal survey)    Funding source: Centre for Mental Health Services | ICM | To assess the effect of the case management relationship on clinical outcomes among homeless persons with serious mental illness | Pan USA Meeting location not reported May 1994 - April 1996 | N=3,481 Average age 39 36% female 45% African American, 6% hispanic; 100% with severe mental illness (Structured Clinical Interview for Diagnosis, SCID), 44% alcohol use disorder, 38% drug use disorder    ETHOS: 1. Living rough or 2. Emergency accommodation  during 7+ of the 14 nights prior to recruitment | Clients' relationship with their case manager was associated with homelessness and general life satisfaction (in both cases p<0.001 for no/low alliance versus high alliance at 12 months) |
| Chinman 2017 | Qualitative: Semi-structured interviews [within cluster randomised trial]    Funding source: Health Services Research and Development Quality, VA Quality Enhancement Research Initiative | CTI | To evaluate how much the implementation strategy facilitated HUD-VASH’s adoption and implementation of MISSION-Vet | USA, three VA Medical Centres Meeting location: Not reported March 2013 - August 2015 | Interviews N=22 18 case managers, six supervisory staff, one peer specialist [doesn't match!]    ETHOS: 3. Accommodation for the homeless | CMs reported that MISSION-Vet was: -confusing - little involvement from leadership - difficulties with data feedback - not enough resource to implement |
| Choy-Brown 2021 | Qualitative: Focus groups    Funding source: National Institute of Mental Health | Housing First | 1. What are the challenges frontline staff and supervisors perceive in delivering services using an HF approach?  2. What strategies are employed to maintain fidelity to HF over time? | 5 Housing First sites, Mid-Atlantic/Northeastern USA Meetings held at HF site Dates not reported | N=33 17 case managers & 16 supervisors 60% female 76% white, 18% black/african American    ETHOS: 3. Accommodation for the homeless | - Lack of affordable housing & choice - Separation of housing and services - Funders' restrictions and practice 'drift' (fidelity to HF) - Importance of community engagement - Need for strong leadership and 'bending the rules' - Attracting knowledgeable staff - Training |
| Clark 2016 | Qualitative: Unstructured Interviews  (within non-randomised controlled trial)    Funding source: Substance Abuse and Mental Health Services Administration: Treatment for Homeless Program | Housing First CTI versus ACT | To discuss staff perceptions, program elements, and housing and behavioural health outcomes of two evidence models of case management | Florida (?), USA Meeting location: Short-term diversion centre (CTI); Not reported (ACT) Dates not reported | N=6 staff members    ETHOS: 3. Accommodation for the homeless | - Some preference for ACT model, primarily because of team support and extended follow through with clients |
| Clifasefi 2016 | Qualitative: Focus groups, interviews & observation    Funding source: National Institue on Alcohol Abuse and Alcoholism | Housing First | To develop a conceptual or thematic description of residents', staff, and management experiences and perceptions of single-site HF and their suggestions for program enhancement | Seattle, Washington, USA One single-site Housing First residence Meeting location not reported June - December 2013 | 3 staff focus groups, plus 7 programme staff and 4 agency management interviews[N=19] Average age 41 53% female White/European American 89%  Interviews with residents [N=44] Average age 53 18% female 43% White/European American, 20% American Indian/Alaska Native/First Nation, 20% more than one race    ETHOS: 3. Accommodation for the homeless | - Enhancing training and support for staff [understanding single-site approach, roles, power dynamics & crisis de-escalation, cultural humility and respect, self care capacity] - increasing residents' access to meaningful activities [but resourcing & residents' concerns about intoxication of others] -exploring alternate pathways to recovery [not abstinence-based] |
| Cole 2017 | Qualitative: In-depth interviews Focus groups Field observations    Funding source: Urban Ministry Center, Charlotte Mecklenburg Community Foundation, Civic By Design | ACT | What individual  and program factors influence exits from Housing First programs among individuals who have experienced chronic homelessness | Mecklenburg County, South Carolina USA HousingWorks Permanent Supportive Housing Meeting location: Urban Ministry Center or HousingWorks Jan 2016 - June 2016 | In-depth interviews Clients exited HousingWorks (n = 14; age ragne 47 - 67; female 43%; Trans 7%; Black 79%, White 14%) HousingWorks directors (n = 3; age range 25 - 58; female 50%; White 67%, Black 33%)  Focus group 1. Clients living in HousingWorks (n = 12; age range 40 - 64; female 50%; Black 58%; White 25%; Native American 17%) 2. Case managers (n = 6; Age range 42 - 63; female 33%; White 100%)    ETHOS: 3. Accommodation for the homeless | - Relationships: (strained or favourable) with friends, family, romantic partners, case managers and neighbours - Identity: Difficulty of settling in developing new identity and relationships during transition into housing - Drug activity within HousingWorks - Quality of staff: Need for CM support and training |
| Collins 2019 | Qualitative: Semi-structured interviews Administrative data/client progress notes    Funding source: Multiple including the US Housing and Urban Development Section IV, the Sisters of Charity Foundation, Dorothy K O'Neill Foundation | ACT? [Authors also refer to ICM] | 1. To develop a descriptive picture of experiences in a Housing First pilot program 2. To examine client barriers to self-sufficiency and staff efforts to help overcome those barriers | Ohio, USA Two HF programs - one for families, one for young adults  Meeting location: Not reported 2013-2015 | N=9 staff interviews (5 supervisors, 4 case managers) 78% female  N=78 clients with admin data [63 with families, 15 single young adults aged 18-24] 94% female Mean age 28 79% black, 16% white, 5% hispanic 91% with mental health, 19% alcohol abuse, 22% drug abuse diagnosis    ETHOS: 3. Accommodation for the homeless | Themes (staff) - Need to support basic living skills - Difficulty in getting appropriate accommodation/predjudice etc. - Retention\burn out of staff [Need for 'flexible, competent, compassionate and consistent case managers']  Themes (clients) - Need for housing assistance and living skills support from CM (intensive support) |
| de Vet 2017 | Qualitative: Chart review Focus groups [Linked to two RCTs]    Funding source: ZonMw, the Netherlands Organization for Health Research and Development, the Academic Collaborative Center for Shelter and Recovery | CTI [adapted & flexible] | To assess fidelity in two service delivery systems and explore factors influencing model adherence | Netherlands 18 homeless shelters Meeting location: Not reported 2010 - 2013 | Focus groups with CTI workers [N=11] No demographic information  Chart review of random selection of clients exiting centres [N=70]    ETHOS: 3. Accommodation for the homeless | Themes from focus groups: Importance of -Continuity of care -Engaging community support [while still in the shelter] -Working (trusting) relationship between client & care worker - Adaptation of support as client adapts to community living - Organisational support and tools/training for case managers  Themes from chart review: - Little difference in fidelity measure |
| First 1990 | Qualitative: Interviews Data review    Funding source: National Institute for Mental Health, Indiana Department of Mental Health | ICM | 1. What are the barriers to implementation of this case management model with people who are homeless and mentally ill? 2. What factors account for success in client placement? | Indiana, USA Meeting location: Not reported Dates: Not reported | Client service data [n=139]  26% female; mean age 27; 51% black, 40% white, 8% hispanic; 93% mental health needs, 43% substance abuse needs, 30% physical health needs    ETHOS: 1. Living rough: Public spaces/external spaces; 2. Emergency accommodation: Overnight shelters and ‘those at risk of homelessness’ | From client service data, problems are: 1. Linking homeless clients to services - lack of contact intensity/inadequate community resources to meet emergency needs 2. Maintaining client contact following placement in housing - intensive contact/monitoring need |
| Fitzpatrick 2011 | Qualitative: Review of key policy documents Interviews Focus groups Community/local government data analysis    Funding source: Newcastle City Council  Your Homes Newcastle | ICM ACT | 1. How effective are the homelessness prevention activities and services delivered 2. To what extent can it be said that services have established a culture of homelessness prevention? 3. How effectively is homelessness prevention activity led, co-ordinated and managed? 4. Is it possible to demonstrate the financial and social policy value of these initiatives? | Newcastle, UK Meeting location not reported 2008-2011 | Key informant interviews [N=20] Managerial   Focus Groups [N=21]: 17 front line staff, 4 housing services officers    ETHOS: 1. Living rough; 2. Emergency accommodation and ‘those at risk of homelessness’ | Importance of - Strategic partnerships/multi-agency working - Leadership/senior level commitment |
| Fleury 2014 | Mixed methods: Semi-structured interviews Focus groups Questionnaires Meeting/minute observation    Funding source: Mental Health Commission of Canada | ACT ICM | To evaluate the implementation process for the Montreal site of the At Home/Chez Soi project | Montreal, Canada Meeting location: Not reported Oct 2009 - Dec 2010 | N=62 37 professionals [15 managers/team leaders/psychiatrists/researchers; 19 service providers; 3 peer user council reps] Average age 39 84% female  25 service users Average age 47 32% female 84% white 56% mental health issues; 28% physical health problems; 39% substance misuse    ETHOS: 1. Living rough | - Lack of support from provincial authorities/key local resources - Many structures, divergent values, frequent personnel turnover, lacking staff supervision, mis communication - Complex/unyielding project - Issues with financing - Issues with commitment - Positives from effective governing structures, training initiatives and toolkits Overall, the project became unsustainable. |
| Flowers 2014 | Qualitative: Semi-structured interviews    Funding source: Mental Health Commission of Canada | ACT ICM | 1. To provide an in-depth description of lessons learned 2. To help the key players reflect on and understand the underlying causes of the issues they would inevitably have to face | Moncton, Canada Meeting location: Not reported Nov 2009 - March 2010 | N=11 Non-profit sector, government, academics No further information provided    ETHOS: 1. Living rough; 2. Emergency accommodation; 3. Accommodation for homeless  Experience: 1 or more episodes of being homeless  for at least 4 weeks | Themes from Planning Phase: - Importance of leadership -Major concerns around ethics of intervention (services as usual to half of recruits) - Identification of the target population and definitional issues - importance of community engagement/centralization of local coordination and key community players - Concerns around sustainability |
| Francis 2000 | Qualitative: Ethnography - Observation & unstructured interview    Funding source: NR | ICM/ACT Described as ICM but multidisciplinary team | 1) what activities comprise intensive case management,  2) how does the system environment affect their implementation? | USA, Large city in the Southeastern U.S. Meeting location: In vivo (including client residence, park benches, local drop-off center) Dates: Not reported | Observation of all agency activities  Interviews [N=10] Six team members, nurse, outreach worker, project manager, project director    ETHOS: NR (likely 1-3) | Themes: - 'Catch 22' tension between federal and county rules. Eg Access to Community Mental Health Services required an address but housing only available to those receiving treatment or assessed as 'clean' - Need to bend rules to access services for clients - risk of burn out due to frustrations associated with bureaucracy 1) Organisation juxtaposed between two systems created tension 2) Case managers resorted to bending and breaking the rules to provide services to clients 3) Agency staff a torn between clients and the system 4) Client drop-outs are an issue for agency staff. They engage clients on a personal level and often experience burnout 5) Case managers experienced role conflict |
| Garcia 2020 | Qualitative: Focus groups Interviews (no data)    Funding source: Research Incentive Seed Grant Program, University of Utah | Housing First | What is the role of the Rapid Rehousing Programe (RRHP) in supporting the security of families experiencing homelessness? | USA, Utah, Salt Lake County Meeting location: Not reported October - November 2019 | N=31 23 families experiencing homelessness Average age 38 70% white; 26% hispanic 52% in two parent households Average no. of children 1.8 40% with disability  2 landlords & 6 case managers & service providers No demographic information    ETHOS: 2. People in emergency accommodation  ‘Previous experience of homelessness (2, 3 or 4 times)’ | -(In)security in the emergency shelter -kids' behaviour in the shelter vs home -(Importance of) feeling at home -feelings of (in)security at the new home |
| Jost 2014 | Qualitative: Semi-structured interviews    Funding source: NR | SCM [including tenant choice of CM] | 1. How satisfied were tenants with their choices/matches 2. How well-informed were tenants’ decisions 3. To what degree did tenants value the option to choose 4. What were staff members’ experiences with Tenant Choice | New York, USA Interviews in private offices at Tenant Choice site Dates: Not reported, circa 2009/2010 | N=40 31 tenants Median age 50 26% female 68% black, 19% white, 13% hispanic 71% psychiatric diagnosis, 61% history of substance abuse, 23% HIV +ve Median homelessness 5 years, medium time at pilot site 2.5 months  9 staff members Median age 33 11% male 44% white, 33% hispanic, 11% black 89% 5+ years social service experience, 56% with Masters Degree    ETHOS: 3. Accommodation for the homeless | - Pre-existing satisfaction drove tenant choice - Personal qualities of CM were valued by tenants over professional qualifications and work experience;  - The views of tenants and staff differed regarding whether minimal contact versus extended contact leads to better choices and working relationships;  - The option of choice was valued by tenants regardless of whether it led to a change of case manager - Staff concerns (around offering tenant choice) failed to materialise |
| Kietzman 2020 | Qualitative: Semi-structured interview Focus group    Funding source: The Robert Wood Johnson Foundation | ICM | To gauge effectiveness and impact on health service utilization and outcomes in other public service sectors | USA, Los Angeles County Permanent Supportive Housing programmes Meeting location: Not reported 2016 - 2018 | N=85 Interview Key informants (n = 14; direct service proviers, housing and homeless services program adminstrators, multisectoral agency leaers)  Focus groups Clients (n = 42) Providers of permanent supportive housing (n = 29)    ETHOS: 3. Accommodation for the homeless | - Extremely limited housing inventory (availability) - Insufficient workforce with high turnover - (Need for) communication and accountability - Importance of cross-sectoral leadership |
| Kirst 2014 | Qualitative: Semi-structured interviews    Funding source: Mental Health Commission of Canada | Housing First ACT (high needs) ICM (moderate needs) | To explore perspectives on hopes for recovery and the role of housing on these hopes | Toronto, Canada Housing First sites and Treatment as Usual accommodation Meeting location: Not reported March 2010 - June 2011 | N=60 36 from Housing First and 24 from TAU group 30% female Mean age 41 55% identified as in ethnoracial group 100% with mental health needs; 44% high needs, 60% moderate needs    ETHOS: 3. Accommodation for the homeless | - Hopes for recovery. Need for clear visualisation of goals for recovery and importance of housing in this respect; - Concerns around housing. Difficulty in adjusting to housing and feelings of social isolation |
| McCarthy 2007 | Quantitative: Secondary analysis of quasi-experimental data    Funding source: Substance Abuse and Mental Health Services Administration | ICM Moderate-intensity CM Low-intensity CM | To examine the effect of direct and indirect ratings of working alliances on concurrent and longer-term health outcomes | 12 programmes, USA, Connecticut Meeting location: Not reported Dates: Not reported | Homeless female clients with substance disorder and a child (n = 90; average age = 32; avearge no. children = 3; Female 100%; White 44%, Hispanic 34%, African American 21%)  Case managers (n = 15; Female 93%; Average age 36; Latino 47%, White 33%, African American 20%)    ETHOS: Various 1-6 | No overall relationship between working alliance between client and case manager and clinical or functional outcomes. [In racially matched diads, high reported working alliance associated with self-reported physical health] |
| Miller 2001 | Qualitative: Interviews Focus group  Documentation review    Funding source: Department of Housing and Urban Development | ACT Described as ICM but multidisciplinary team | To assess how stakeholders understand the Community Residence programme mission, goals and activities, and the extent to which activities implemented and facilitated are consistent with the mission and goals | New York, USA Focus group held at Family Community Residence 1999-2000 | Interviews Executive level management (including President, CEO, Senior VP of Quality Management, Executive VP of Clinical Operations, VP of Mental Health, Rehab and Support, Board member, n = 8) Programme staff (including Director, Program supervisor, Family Development Specialist, Case managers, Psychologist, Substance Abuse Counselor, Front-Desk Attendances/Counselors, n = 17)  Focus group Current and former clients (15 single parents. All with custody of, or process of regaining custody of children; 100% mental health diagnosis    ETHOS: 3. Accommodation for the homeless | Executive & Staff:  - Lack of funding - Lack of appropriate staff - Accommodation options not fit for purpose - Need for families to move out in given time frame - (Importance of) independence and confidence building - (Risk of) programme fostering dependency - Training needs for residents  Residents:  - Stigma of mental illness - Day to day stress of parenting - Managing mental illness - (Concerns around) custody of/contact with children |
| Montgomery 2017 | Mixed methods: Semistructured interviews Surveys Administrative data (Medical records) Progrom data (VA HOMES)    Funding source: U.S Department of Housing and Urban Development (HUD) | ACT | 1. What are common barriers that prevent Veterans from using HUD-VASH vouchers or making permanent housing difficult 2. What are the reasons that Veterans exit the HUD-VASH program 3. What different types of exits are there, and do reasons for exit differ across exit types 4. What Veteran characteristics or program antecedents predict outcomes 5. What are Veterans’ patterns of services use before, during, and after participation  6. What community factors impact success 7. How do HUD and VA work together to implement HUD-VASH at the community level | Four urban settings (Houston, Los Angeles, Palo Alto, Philadelphia) USA Meeting location: clients home 2008 - 2014 | Survey: N=508 veterans 72.8% stayers >85% male >65% black >45% in receipt of compensation for service-connected disability >30% mental or other health condition   Interviews: N=110 veterans 50.9% stayers   Staff/Case Managers (N?)  Also data from all veterans  (total n = 7383; Stayers n = 2693; Leased-Up Exiters n = 2999; Nolneased Exiters n = 1423) Stayers (Black/African American 60%, White 34%, Hispanic/Latino 7%; Mental or behavioural health condition 34%) Leased-Up Exiters (Black/African American 57%, White 37%, Hispanic/Latino 8%; Mental or behavioural health condition 40%) Nonleased Exiters (Black/African American 45%, White 48%, Hispanic/Latino10%; Mental or behavioural health condition 38%)    ETHOS: Various 1-6 | Participants:  - (Importance of) relationship with case manager/supportive CM - Access to peer support - Cooperative landlords/ability to access suitable housing/lists of available housing - Move-In assistance - Personal ability to meet requirements of programme - Ease of enrolment process to HUD-VASH - Difficulties with programme rules & regulations - High staff turnover  Staff: - Relationships with community partners [including in identifying veterans in need of assistance] - Ability to access community resources and connect veterans to these - Access to staff training - Programme flexibility - Specialist help for employment and peer support - Building trust with veterans - Supporting veteran independence |
| Montgomery 2019 | Qualitative: Focus groups    Funding source: US Department of Veterans Affairs, National Center on Homelessness Among Veterans | ACT SCM | To explore how the HUD-VASH program applies Housing First principles and how the programs were influenced by Housing First principles and specific strategies to implement these principles in a single-site setting. | Five urban communities, USA Meeting location: Not reported May - July 2017 | N=64 HUD-VASH staff Program leaders, case managers, peer support specialists, landlords, property managers, community organisation reps, public housing authority staff    ETHOS: 3. Accommodation for the homeless | - The importance of leveraging the independent yet overlapping tasks of case management and property management to ensure functional – if not geographic – separation of housing and services - Importance of housing choice for clients - Maintaining staff on-site to address Veterans’ needs  - Working with community service organizations to complement the array of service available to residents - Housing single-site programs in mixed-use buildings; facilitating integration into community |
| Newman 2017 | Quantitative: Survey (telephone and online)    Funding source: NR | SCM? | To determine the best practices in emergency shelter delivery to homeless men based on top agency representatives' opinions | Emergency shelters throughout USA 19/21 shelters in urban areas Meeting location: Not reported July 2014 | N = 21 emergency shelter leaders Telephone or online    ETHOS: 2. People in emergency accommodation | Importance of case management Need for security Vital services - those that address basic needs |
| Patel 2013 | Qualitative: Focus groups Formative evaluation (semi-structured interviews and data analysis)    Funding source: NR | ACT | To examine patient characteristics, intervention processes, emergency department (ED) utilisation patterns, staff perceptions on perceived barriers | West Los Angeles, USA Meeting location: Not reported Feb-April 2012 (focus groups) Jan 2013 (formative evaluation)  Single medical centre team | 10 Focus Groups of administrators and patient carers (p.62);  Interviews with 14 key informants 7 administrative, 7 clinical 43% having daily contact with veterans    ETHOS: Various 1-6. Major groupings 1. Living rough; 3. Accommodation for Homeless; 6. With family/friends | Related to case management (from interviews)… Where more than half respondents felt the following reduced ED utilisation amongst homeless veterans (Q5d p. 89):  - Care coordination/case management 71%  - Coordination across programmes 57% - Organisational capacity 57% - (Avoiding) gaps in care ('warm handoffs to connect with services required') 57% |
| Ploeg 2008 | Qualitative: Semi-structured interviews  Focus groups Data from client records    Funding source: Canadian Health Services Research Foundation, Ontario Ministry of Health and Long-term Care, Canadian Institutes of Health Research | SCM? | To describe: (1) how the Homelessness Intervention Programme addressed the needs of elderly people and (2) the factors that influenced the ability of the programme to address client needs | Urban Ontario, Canada 9/10 interviews in agency office Dec 2001 - March 2004 | Interviews: N=10 (1 client, 3 programme staff, 4 service admin, 3 programme funders  Focus groups: N=17 (5 clients [80% female; all 50-60 years old, 4 with mental health & 4 with medical conditions], 4 programme staff, 8 housing providers)  Data from all 129 individuals receiving HIP during study period All 54 years or older; 50% female; 35% medical condition; 26% mental health' 23% substance misuse    ETHOS: 1. Living rough or ‘Homeless or at risk of homelessness’ | Value of a continuous caring relationship with an identified provider: Delivery of seamless service through coordination, integration & information sharing among providers; Housing options available: Income support available: Central finding is continuity of care |
| Ponce 2018 | Qualitative: Focus groups    Funding source: Substance Abuse and Mental Health Services Administration, State of Connecticut Department of Mental Health and Addiction Services | ACT? (team approach but no focus on mental illness) | To explore perceptions of this form of service delivery and the benefits and challenges of the program model from the perspective of those who experienced it. | New Haven, Connecticut, USA Meetings in Agency Dates: No reported | N=17 (5 participants, 6 programme staff, 4 non-PTI case managers in parent agency, 1 landlord, 1 employer)    ETHOS: NR (‘chronic homelessness’) | Importance of  - Relationships (staff-staff and staff-participant) - Staff specialization and provision of individualized services - comprehensiveness of services and facilitation of connections to community resources   - An orientation to outcomes (viewed as positive and negative) - Systems challenges (eg access to housing vouchers, meeting criteria for support) |
| Quinn 2018 | Qualitative: Semi-structured interviews    Funding source: National Institute on Drug Abuse | ICM/SCM? [CM case load 15-25] | To qualitatively examine supportive housing providers’ experiences and challenges with housing chronically homeless individuals and explore opportunities to improve supportive housing systems of care. | Chicago, USA 30 PHI agencies (some Housing First) Meetings in participant offices 2014 | N=65 32 programme administrators and 33 case managers    ETHOS: 3. Accommodation for the homeless | Housing priorities - Importance of residents engaging with services to increase likelihood of success Funding cuts - Increasing needs of residents not matched by funding Co- ordinated entry - Difficulties in ensuring entry to service that best meets individual's needs/'creaming tactics' to enhance apparent success Permanency of housing - leading to slow turnover and lack of suitable housing outside reducing opportunities for independence |
| Richardson 1996 | Mixed methods: Questionnaire Interviews Case review    Funding source: NR | SCM | To detail 'recovery from homelessness' through analysis of descriptive demographic data and correlation of success rates with participant behaviour and intervention context | Eastern Iowa, USA Meeting location:Not reported July 1990 - Dec 1995 | N = 51 interviews with former participants at programme exit No data  N= 217 questionnaires from family heads  100% female Average age 27 35% minorities 32% reporting chronic medical problem; 17% had been incarcerated; 58% experienced violence at home; 21% substance misuse issues    ETHOS: 3. Accommodation for the homeless | Strong relationship between case manager and client Sustained intervention (continuity)   Both correlated with time spent in the programme |
| Roberts 2012 | Mixed methods: Client satisfaction survey Document review Semi-structured interviews with senior management    Funding source: NR [PhD at the University of Rhode Island] | SCM | To explore clients' needs and satisfaction with a range of services offered by the shelter in 2011 compared to data in 2009 (which was followed by an intensive period of change] [In 2011] key informant interviews to identify changes and their perspectives | Providence, Rhode Island, USA Meeting location: Private room in Crossroads facility 2009 | N=100 [client satifaction survey in 2011]  42% female 23% under 30; 6% over 60 48% white, 25% african-american, 19% hispanic  N=4 Crossroads senior management team members [semi-structured interviews]  N=212 [client satisfaction survey in 2009]      ETHOS: 2. People in emergency accommodation | In 2009  - Overall high levels of satisfaction with case managers - CM perceived as respectful and helpful with emergency resources - CM perceived as less helpful with clients making progress on individual goals - Concern about inconsistent treatment of clients by different case managers  Task force recommended that Crossroads revisit staffing patterns, define caseload management model and reassess how cases are assigned. Development of staff training including de-escalation skills.  2011 - Overall high levels of satisfaction with case managers - Clients frustrated by not being able to procure permanent housing and had difficulties with transport - Lack of consistency in application of rules across case managers  [See note about quantitative data suggesting positive response bias in relation to CMs] |
| Rog 1997 | Mixed methods: Survey in 1993 Semi-structured interviews in 1995    Funding source: Robert Wood Johnson Foundation | SCM ICM | To provide an in-depth look at the structure and operation of case management in services-enriched housing. | 9 urban settings, USA Meeting location: Clients home Dates: 1990 - 1995 | Survey: N=98 case managers in 1993 91% female 69% in their 30s/40s 'racial mix' 42% professional certificate/licence 63% ≤ 5 years’ experience  Key Informant interviews (Providers, Agency officials , Advocates, HFP project directors, Key stakeholders) No further detail  Case manager time sample N=57    ETHOS: 1. People living rough; 2. People in emergency accommodation | Themes (imputed from findings/discussion) related to CM case-load: - CM paperwork required limits time available for families; difficult to achieve planned intensity - flexible approach tending to be the norm; some families requiring intensive (eg weekly) support |
| Shepherd 2019 | Qualitative: Interviews    Funding source: Queensland Government | SCM? | To examine the division of labour that emerged between support workers and government health workers within a cross agency supported housing program  To understand the nature of the working relationships between the clinical case managers and the non-clinical support workers involved in delivery of services at the coalface | 19 settings [10 urban]Queensland, Australia Meeting location: Not reported 2011 | N=77 40 government funded mental health case managers; 37 staff [27 support workers & 10 managers] from non-government agencies (NGOs)    ETHOS: NR | - Communication needed to support understanding/maintenance of role boundaries across clinical and non-clinical roles - High case-loads of CMs - Role ambiguity, and support workers feeling their input and feedback were not valued by government health workers. |
| Solomon 1994 | Quantitative: Surveys at 0 and 6 months post jail release (within RCT)    Funding source: National Institute of Mental Health | ACT, ICM or as usual | [Linked to a trial] To examine the relationship between reported receipt of services clients stated they needed and whether clients returned to jail within six months of release. | Large urban centre, USA Meeting location: Not reported Dates: Not reported | N=105 Authors state similar demographics to 200 clients released from jail: 12% female Average age of those known = 35 83% black, 14% white, 3% hispanic 100% serious mental health condition (86% schizophrenia, 11% major affective disorder, 3% other)    ETHOS: 4. People living in institutions | - Greater number of clients receiving ACT returned to jail compared to ICM clients - Clients who returned to jail reported receiving fewer of the services they needed   The only item significantly associated with return to jail was receipt of a training programme to help clients take care of themselves. Clients who were arrested within six months were more likely to say they did not receive this service although it was needed |
| Stanhope 2007 | Qualitative: Focus groups Interviews    Funding source: National Institute of  Mental Health | ACT | How do coercion and consumer-provider relationships operate within the clinical setting of ACT services | Philadelphia, USA Meeting location: Office, or other location Dates: Not reported | Focus groups Homeless clients with severe mental illness (n = 12; average age 46; female 50%; African American 67%, White 33%) Case managers (n = 12; average age 42; female 50%; African American 67%, White 8%, Asian American 8%, Other 8%)  Interviews ACT team (total n = 3 (supervisor n = 1; Case managers n = 2); average age 33; Female 66%; White 100%) Homeless clients with severe mental illness (n = 3; average 34; female 66%; African American 100%)    ETHOS: NR | Relationships 1. Human connection 2. What consumers want from case managers 3. What case managers want from consumers 4. Not wanting too much  Coercion 5. Talking about consequences 6. Verbal contracting  Case managers viewed the relationship as a means to obtain service goals, whereas consumers valued these relationships for their own sake. Shared themes: - reliability - building trust (by small gestures) - providing social support - importance of mental health stability - consumers open to working with CM - providing services the consumer wants |
| Stanhope 2015 | Qualitative: Multiple semi-structured interviews (longitudinal)    Funding source: National Institute of Mental Health | ICM | To explore the ways in which case managers working in a supportive housing program approach treatment and how their approach is influenced by both program requirements and their beliefs about mental illness | New York City, USA Meeting location: Case manager's agency or study office Dates: Not reported | N=24 case managers 46% female 87% Black/African American; 96% batchelor's degree or less; 75% < 3 years employment at agency    ETHOS: 3. Accommodation for the homeless | - Case managers belief on medication adherence as key to success of supportive housing for clients with severe mental illness - Authors conclusion around need for CM training re mental health recovery and integrated health care |
| Stergiopoulos 2012 | Qualitative: Focus groups Interviews [Data collected within an RCT]    Funding source: Mental Health Commission of Canada | ICM | 1. To describe the fidelity of the program 2. To report on program provider and program participant perspectives on the model 3. To identify challenges and facilitators of successful early implementation | Toronto, Canada Meeting location: Client's residence March 2010 - June 2011 | Focus groups: Homeless people with mental illness from ethno-racial groups: 10 intervention and 5 control group participants;   Focus groups: 7 HF ER-ICM programme staff; 4 Housing Team staff; 12 consumer caucus members  Key informant interviews: Principal investigator from research team, Site study coordinator, representative of City of Toronto housing team, Director of HF ER-ICM team, HF ER-ICM team lead  Participant interviews: 25 intervention and 12 control group participants  No data for interviewees. Overall HF ER-ICM participants (N=204) 34% female Mean age 39 54% Black, 10% South Asian, 11% mixed, 7% East Asian; 6% East Asian 100% mental health diagnosis, 25% current substance dependence, 15% current alcohol abuse, 29% moderate/high suicidality    ETHOS: 1. People living rough (primarily, 91%) | In relation to Housing First: - (Importance of) participant choice and separation of housing and services -Requirement for additional training in harm reduction and motivational interviewing, and expanding links with brokerages services (particularly employment/education/substance abuse & health services) -Service environment welcoming to racialized communities and advocacy for system-level changes -Appreciation of CM support within client-driven holistic framework -Attempts to hire staff reflecting cultural and linguistic diversity of clients |
| Toombs 2021 | Qualitative: Interviews Focus group [Data collected within an effectiveness study]    Funding source: NR | CTI | To assess the feasibility of continued implemen- tation of the HOP-C North program with Indigenous youth through qualitative interviews with program staff and participants | Thunder Bay, Ontario, Canada Meeting location: Not reported Dates: Not reported | Participants (N=15 for initial interview; 8 for follow-up) Of the 15: 73% female Mean age 19 60% indigenous, 40% mixed/other heritage 73% heterosexual 47% reporting visit to hospital for mental health concern  Staff (N=14) in interviews/focus group    ETHOS: 3. Accommodation for the homeless | 1. Clinical effectiveness 2. Enhanced independence 3. Increased trust in organizational staff 4. Participant goal setting 5. Staff perceptions of participant outcomes (hope, employment, educational, caring, hygiene) 6. Program accessibility 7. Voluntary participation 8. Emphasis on building individual and organizational relationships 9. Relevant cultural programming  Themes (from 8 client follow up interviews and 14 staff): - valuing program flexibility - emphasis on relationships and trust - relevance of cultural adaptation/programming - postering participant autonomy/voluntary participation - adaptive approach to programme implementation |
| Tsai 2014 | Quantitative and Qualitative: Survey including two open ended questions    Funding source: Department of Veterans Affairs | ICM | To evaluate satisfaction with a novel group-intensive peer support (GIPS) model of case management for the HUD-VASH programme. | Single site, Connecticut, USA Meeting location: Community, clients home December 2010 - February 2011 | N=95 Clients attending groups as part of the GIPS model and those who did not.  Group attenders (N=73) 11% female; Mean age 52; 47% black, 43% white; Average 4.8 lifetime years homeless; 64% once to several times per week case manager contact  Non group attenders (N=22) 23% female; Mean age 52; 55% whilte, 36% black. Average 3.2 lifetime years homeless; 14% once to several times per week case manager contact    ETHOS: 3. Accommodation for the homeless | Liked best - Staff or staff characteristics (highest satisfaction score from both groups) - Social relationships/peer support/groups - Housing/apartment/end of homelessness - Information resources  Liked least - Meeting times, frequency, or locations of groups - Disruptive veterans/lack of peer support or participation |
| Vitopoulos 2018 | Qualitative: Field notes Interviews    Funding source: Ontario Ministry of Children and Youth Services and the Toronto Centre for Addition and Mental Health Foundation | CTI | To describe the process of design, development, and implementation of a complex, multi-component, tertiary prevention intervention aimed at stabilizing pathways out of homelessness (aimed at youth within 12 months of being housed) | Canada, Ontario, Toronto Meeting location: Not reported Dates: Not reported | N=10 staff interviews (service leaders, case managers, psychologists, peer support workers, creative arts programme specialists); Also field notes    ETHOS: 3. Accommodation for the homeless | Value of case management (?check) - Difficulty of integrating with pre-existing services to youth - Value of peer workers - Importance of mental health support - Importance of team meetings and engagement/collaboration across services |
| Zerger 2016 | Qualitative:  Semi-structured interviews Focus groups    Funding source: Health Canada, Mental Health Commission of Canada | ICM ACT | (1) what non-structural factors contribute to undue delays and relocations during implementation of a HF program? and 2) How do service providers and participants experience and respond to these situations? | Canada, Toronto Meeting location: Consumer housing unit July 2012 - November 2012 | N=48 23 staff (15 case managers, three housing workers, five team managers)  25 consumers 20 housed and 5 never-housed 32% female Average age 42 20% white Canadian, 16% black Canadian, 12% South Asian 100% mental illness    ETHOS: 1. Living rough | Themes relating to housing delays and transfers: -Communication and collaboration amongst consumers and service providers [Noted the importance of the 'therapeutic alliance' between case manager and consumer] -consumer-driven preference and ambivalence -provider prioritisation of consumer choice over immediate housing access; shared understanding of consumer choice is a facilitator [Noted the value of consumer participation in the housing search] - Structured communications |

## 5 Appendix 5:  Excluded intervention studies

| **Authors** | **Year** | **Exclusion reason** |
| --- | --- | --- |
| D. Allen; E. Feinberg; H. Mitchell | 2014 | No comparison group |
| J. Baker; J. Travers; P. Buschman; et al. | 2018 | No comparison group |
| J. F. Bell; A. Krupski; J. M. Joesch; et al. | 2015 | Wrong population (not homeless) |
| M. Bertelsen; P. Jeppesen; L. | 2008 | Wrong population (only small % homeless) |
| N. A. Bitter; D. P. Roeg; C. V. Nieuwenhuizen; et al. | 2015 | Wrong population (not homeless) |
| M. Brown; L. Klebek; G. Chodzen; et al. | 2018 | Comparison group not matched |
| M. Brown; M. Rowe; A. Cunningham; et al. | 2018 | No comparison group |
| D. R. Buchanan | 2007 | Superseded by Buchanan 2009 |
| A. M. Cauce; C. J. Morgan; V. Wagner; et al. | 1994 | No comparison group |
| B. Chan; S. T. Edwards; M. Devoe; et al, | 2018 | Wrong study type - trial protocol |
| M. J. Chinman; R. Rosenheck; J. A. Lam | 2000a | No comparison group |
| M. J. Chinman; R. Rosenheck; J. A. Lam; et al. | 2000b | No comparison group |
| C. Clark; C. C. Guenther; J. N. Mitchell | 2016 | No comparison group |
| C. Clark; A. R. Rich | 2003 | Both groups same case management intervention |
| G. N. Clarke; H. A. Herinckx; R. F. Kinney; et al. | 2000 | Wrong population (not homeless) |
| P. W. Corrigan; D. J. Kraus; S. A. Pickett; et al. | 2017 | Not a case managment intervention |
| K. B. Cox; C. A. Malte; A. J. Saxon | 2017 | No relevant outcomes (service use measurement linked to Malte 2017) |
| L. Dixon; E. Kernan; N. Krauss; et al. | 1997 | No outcomes reported |
| R. E. Drake; G. J. McHugo; R. E. Clark; et al. | 1998 | Wrong population (not homeless) |
| G. Duwe | 2012 | Wrong population (ca 75% housed) |
| G. Duwe | 2013 | Wrong population(ca 75% housed) |
| C. J. Felton; P. Stastny; D. L. Shern; et al. | 1995 | Comparison group not randomised and not matched |
| P. J. Fowler; M. Schoeny | 2015 | Wrong population (vast majority housed albeit inadequate housing) |
| D. Goeman; J. Howard; R. Ogrin | 2019 | No comparison group |
| P. Goering; S. Veldhuizen; G. B. Nelson; A. et al. | 2016 | No relevant outcomes (fidelity rating scale assessment) |
| R. J. Gordon; R. A. Rosenheck; R. A. Zweig; et al. | 2012 | No comparison group |
| M. Grace; P. R. Gill | 2016 | No comparison group |
| S. K. Harris; C. L. Samples; P. M. Keenan; et al. | 2003 | Wrong population (not homeless) |
| D. Hoffman; R. Rosenheck | 2001 | No comparison group |
| M. Horvitz-Lennon; D. L. Zhou; S. L. T. Normand; et al. | 2011 | No comparison group |
| C. I. Hultman; K. J. Conrad; A. R. Pope; et al. | 1995 | Superceded by Conrad 1998 |
| S. B. Hunter; A. Scherling; M. Felician; et al. | 2020 | No comparison group |
| Isrctn | 2018 | No full text available |
| W. J. Kasprow; R. A. Rosenheck | 2007 | No comparison group |
| M. W. Kirby; G. N. Braucht; E. Brown; et al. | 1999 | No outcomes reported |
| A. R. Klein | 1997 | Wrong population (not homeless) |
| W. D. Klinkenberg; R. J. Calsyn; G. A. Morse | 1998 | No comparison group |
| J. Leopold; S. Adeeyo; M. Cohen; et al. | 2016 | No comparison group |
| J. B. Milby; J. E. Schumacher; D. Wallace; et al. | 2010 | Not a case management intervention |
| D. T. Moore; R. A. Rosenheck | 2017 | Comparison group not randomised or matched |
| G. A. Morse; M. M. York; N. Dell; et al. | 2020 | No comparison group |
| Nct | 2007 | No relevant outcomes |
| Nct | 2014 | Not a case management intervention (CTI training package for managers) |
| M. Nordentoft; J. Øhlenschlæger; A. Thorup; et al. | 2010 | Wrong population (low % homeless) |
| P. O'Campo; S. W. Hwang; A. Gozdzik; et al. | 2017 | No relevant outcomes (study attrition only) |
| D. K. Padgett; L. Gulcur; S. Tsemberis | 2010 | Comparison group not matched |
| K. J. Pierce | 1999 | Superceded by Morse 2006 |
| A. R. Pope; K. J. Conrad; W. Baxter; et al. | 1993 | Superceded by Conrad 1998 |
| J. M. Reingle Gonzalez; M. S. Businelle; et al. | 2018 | Wrong study type - trial protocol |
| H. Rolston; J. Geyer; G. Locke; et al. | 2013 | Wrong population (at risk but 90% housed at baseline) |
| A. B. Rothbard; S. Y. Min; E. Kuno; et al. | 2004 | No comparison group |
| N. Slesnick; G. Erdem | 2012 | No comparison group |
| N. Slesnick; M. J. Kang; A. E. Bonomi; et al. | 2008 | No comparison group |
| R. J. Smith; J. L. Jennings; A. Cimino | 2010 | Wrong population (not homeless) |
| M. R. Sosin; M. Bruni; M. Reidy | 1996 | No full text available |
| M. R. Sosin; J. Schwingen; J. Yamaguchi | 1993 | Wrong population (low % homeless) |
| G. J. Stahler; T. F. Shipley, Jr.; D. Bartelt; et al. | 1996 | No full text available |
| S. Tsemberis | 1999 | comp group unmatched and not controlled for in analysis. |
| S. Veldhuizen; C. E. Adair; C. Methot; et al. | 2015 | No relevant outomes (attrition only reported) |
| D. A. Wasylenki; P. N. Goering; D. Lemire; et al. | 1993 | No comparison group |
